# CIGESMED for divers: Establishing a citizen science initiative for the mapping and monitoring of coralligenous assemblages in the Mediterranean Sea

**DOI:** 10.3897/BDJ.4.e8692

**Published:** 2016-11-01

**Authors:** Vasilis Gerovasileiou, Thanos Dailianis, Emmanouela Panteri, Nikitas Michalakis, Giulia Gatti, Maria Sini, Charalampos Dimitriadis, Yiannis Issaris, Maria Salomidi, Irene Filiopoulou, Alper Doğan, Laure Thierry de Ville d’Avray, Romain David, Melih Ertan Ҫinar, Drosos Koutsoubas, Jean-Pierre Féral, Christos Arvanitidis

**Affiliations:** ‡ Institute of Marine Biology, Biotechnology and Aquaculture, Hellenic Centre for Marine Research, Heraklion, Greece; § Mediterranean Institute of Biodiversity and marine and terrestrial Ecology (IMBE), Aix Marseille Université/CNRS/IRD/Université d’Avignon, Station Marine d’Endoume, Marseille, France; | Department of Marine Sciences, School of Environment, University of the Aegean, Mytilene, Greece; ¶ National Marine Park of Zakynthos, Zakynthos, Greece; # Institute of Oceanography, Hellenic Centre for Marine Research, Anavyssos, Attiki, Greece; ¤ Department of Hydrobiology, Faculty of Fisheries, Ege University, Bornova, Izmir, Turkey

**Keywords:** Coralligenous assemblages, Coralligenous outcrops, coralline reefs, bioherms, biodiversity hotspot, monitoring, citizen science, SCUBA diving, Mediterranean Sea

## Abstract

**Background:**

Over the last decade, inventorying and monitoring of marine biodiversity has significantly benefited from the active engagement of volunteers. Although several Citizen Science projects concern tropical reef ecosystems worldwide, none of the existing initiatives has yet specifically focused on their Mediterranean equivalents. Mediterranean coralline reefs, known as “coralligenous”, are bioherms primarily built by calcifying rhodophytes on hard substrates under dim-light conditions; they are considered hotspots of biodiversity and are extremely popular among divers due to their complex structure, conspicuous biological wealth and high aesthetic value. Nevertheless, data on their distribution, structure and conservation status is lacking for several Mediterranean areas while they are vulnerable to an increasing number of threats.

**New information:**

In the framework of CIGESMED SeasEra (ERAnet) project a specialized Citizen Science project was launched, aiming to engage enthusiast divers in the study and monitoring of Mediterranean coralligenous assemblages through the gathering of basic information regarding their spatial occurrence, assemblage structure and associated pressures or threats. For its active implementation, a data collection protocol and a multilingual website were developed, comprising an educational module and a data submission platform. Georeferenced data reporting focuses on: (a) basic topographic and abiotic features for the preliminary description of each site, and the creation of data series for sites receiving multiple visits; (b) presence and relative abundance of typical conspicuous species, as well as (c) existence of pressures and imminent threats, for the characterization and assessment of coralligenous assemblages. A variety of tools is provided to facilitate end users, while divers have the choice to report additional information and are encouraged to upload their photographs. The long-term goal is the development of an active community of amateur observers providing widespread and ecologically significant data on coralligenous assemblages.

## Introduction

Active involvement of volunteers has greatly assisted inventorying and monitoring of marine biodiversity, over the last decade (Thiel et al. 2014). Notable Mediterranean examples of Citizen Science (CS) initiatives focusing on marine biodiversity concern the study and monitoring of vulnerable species (Goffredo et al. 2004, Bramanti et al. 2011), gelatinous plankton aggregations (Boero et al. 2013, Boero et al. 2009), fish assemblages (Arvanitidis et al. 2011, Azzurro et al. 2012, Bulleri and Benedetti-Cecchi 2014), non-indigenous species (Zenetos et al. 2013, Bodilis et al. 2013), or a combination of several diversity features (e.g. Seawatchers). Although a plethora of CS projects deal with tropical coral reefs (e.g. Hodgson 2001, Marshall et al. 2012, Branchini et al. 2014) at a global scale, none has focused on their Mediterranean counterparts.

Mediterranean coralline reefs, widely known as "coralligenous" (from the french term "coralligène", *sensu* Marion 1883), are bioherms built by calcifying rhodophytes on hard substrates under dim-light conditions. They are considered hotspots of biodiversity, harbouring rich assemblages and valuable biological resources (Ballesteros 2006). Coralligenous beds and their assemblages are extremely popular among SCUBA divers due to their complex structure, conspicuous biological wealth and high aesthetic value. Nevertheless, data on their distribution, structure and conservation status is lacking for several Mediterranean areas (e.g. southern and eastern Mediterranean regions) while they are vulnerable to an increasing number of threats (Giakoumi et al. 2013, Martin et al. 2014, Gatti et al. 2015).

The CIGESMED SeasEra project aims to enhance understanding on the links between natural and anthropogenic pressures and ecosystem functioning to define and maintain the “Good Environmental Status” (GES) of the Mediterranean Sea, through the integrated study of coralligenous assemblages. In the framework of CIGEDMED SeasEra project, a specialized CS initiative was launched aiming to engage enthusiast divers in the study of this Mediterranean habitat in order to obtain information regarding its spatial distribution, enable a primary characterization of the basic structure of its assemblages, and monitor potential pressures and threats to the habitat. The initial development of this pilot CS project was based on previous experience of the IMBBC (Institute of Marine Biology, Biotechnology and Aquaculture, HCMR) research group and CIGESMED partners on citizen science pilots design and implementation, as well as on the human, hardware and software resources of the LifeWatchGreece Research Infrastructure, a landmark in the European Strategy Forum on Research Infrastructures (ESFRI) roadmap. This paper aims to describe the background, basic principles and structure of the specialized CS initiative, along with the developed infrastructure, tools and guidelines that will support the ongoing collection of data on a Mediterranean scale.

## Materials and Methods

### Preparatory phase and methodological concept

Past experience from CS projects showed that it is relatively easy to design a CS approach and address volunteers but hard to ensure continuous production and flow of useful and reliable data (Arvanitidis et al. 2011). In retrospect, a key aspect in the design of this pilot project was the collection of data that are: (a) relatively easy to acquire and report, based on the overall, underlying principle "keep it as simple as possible", and (b) reliable and fail-proof. In order to develop a CS protocol that would fulfill the objectives of CIGESMED project, we first created a list of the main types of data that can plausibly be gathered by citizen scientists, based on feedback from scientists that are actively involved in the study of coralligenous assemblages. The complexity and difficulties in the proper definition and description of this specific habitat were also considered. Once available, such information is critical for the ecological assessment and management of these communities. During the consultation and design process, two main groups of information requirements were specified:

***A. Identification of new sites hosting coralligenous assemblages***

This is particularly important given the considerable paucity of spatial information regarding the presence of coralligenous bioherms in the eastern and southern Mediterranean regions, and the high cost of conducting extensive surveys to gather relevant data, since this habitat type usually develops in relatively deep waters (i.e. average of 20 m or even deeper in the eastern Mediterranean).

***B. Spatial recording of pressures and imminent threats***

Such baseline information is key in the design of effective management plans for the conservation of coralligenous assemblages in different parts of the Mediterranean.

### Identifying the profile of citizen scientists

Citizen science approaches maximize their potential when appropriately designed and implemented to address specific scientific goals (Bonney et al. 2014). The main aim of *CIGESMED for divers* CS initiative is to create a network of dedicated coralligenous observers spanning over the Mediterranean Sea. By creating a network of bilateral communication, scientists can address specific scientific / conservation issues that may arise in different areas (e.g. assessing changes in the structure of communities, or the extent of a sporadic mass mortality event) and gather valuable qualitative and semi-quantitative information with minimum cost. The majority of CS initiatives aim to maximize citizen engagement. However, 58% of the marine-related CS projects concentrate in easily accessible habitats such as beaches, estuaries, reefs and seagrass beds, which are found in waters shallower than 10 m (Thiel et al. 2014). Due to the specific diving time and depth limitations of recreational SCUBA diving activities, only 28% of the marine-related CS projects consider depths between 10 and 40 m, while another 14% focuses in deeper waters (>40 m) (Thiel et al. 2014).

Coralligenous bioherms usually develop in circalittoral waters, with their upper distribution limit ranging from less than 20 m depth in the western Mediterranean regions to more than 30 m in the eastern basin, although exceptions can be found in both areas depending on the prevailing environmental conditions (Ballesteros 2006, Sini et al. 2015). Given the aforementioned constraints, the natural complexity and distribution patterns of this habitat type, the CIGESMED CS project could not be based on the massive engagement of citizens-fun divers alone. Instead the project’s main target group is "dedicated divers", i.e. dive enthusiasts who dive at depths greater than 20 m, and exhibit a particular interest for underwater life and the conservation of their diving spots (e.g. local diving associations, naturalists and underwater photographers). According to the preliminary research and previous experience of CIGESMED partners, many divers fitting to this profile are active participants in existing marine life fora and networks (e.g. DORIS, BioObs), and are positively inclined towards using a platform that allows systematic sharing of their observations. Keeping these considerations aside, the designed CS module is openly addressing all amateur divers who are able and willing to contribute information on the occurrence of coralligenous bioherms, alongside specific information on conspicuous coralligenous species, existing pressures and threats, at any place they carry out their diving activities. In addition, the online platform could serve as an online repository for existing unpublished relevant data from various sources.

### Website development and mobile applications

For the active implementation of the designed CS protocol, a portal supporting multilingual content was developed. It is currently available in 6 languages: English, French, Greek, Italian, Turkish, and Spanish. For the website development all available options were considered and Drupal® was selected as the platform that could optimally satisfy the demanding requirements of the designed website. Flexibility, stability and an API framework that can accommodate almost all conceivable functionality were the key characteristics for choosing the specific Framework - CMS (Content Management System). The website uses the Bootstrap 3.x, the most popular HTML, CSS and JS framework for development of complex dynamic websites and web applications, providing an easy way to adapt a responsive web design layout to the final template. All technologies involved in the development process are Open Source, based on the well-tested LAMP Platform (acronym for Linux, Apache, MySQL and PHP) and most used in WWW. For map display functionality Leaflet library and the Openlayers 3.x were used combined with the Google Maps API v.3.

An advanced user management system was developed, with three different groups of registered users: (a) *divers*, who can create, edit and delete content about observations, (b) *country managers*, being able to verify new user registrations, and (c) *website administrators*. Based on permissions set for those groups, each registered user has access to various features of the website. The demand of a complex observation registration form was covered by providing the user with the option to choose between different form displays when the user creates or edits an observation.

A mobile application, that was originally developed in the framework of the LifeWatchGreece infrastructure, was modified and extended to support the present CS initiative. For this reason, a specialized "Citizen Science" sub-application was developed by utilizing Unity3D Platform and C# scripting language.

### Field trials and preliminary data gathering

Field trials for the implementation and optimization of the developed CS protocol took place in three countries: France (Marseille), Greece (National Marine Park of Zakynthos) and Turkey (Izmir). These included briefings to volunteer divers from local dive centres and associations, experimental dives for data collection followed by interviews, questionnaires and discussions, so as to come up with a more simplified list of requirements that clearly address the CIGESMED project objectives and make the data collection and reporting procedures as easy as possible for the participants. During these trials, the idea of developing an educational CS module to ensure a basic understanding of coralligenous bioherms and their associated communities also emerged, and was subsequently constructed.

Preliminary data gathering was performed by the researchers and divers involved in CIGESMED project, thus allowing the assessment of coralligenous sites in four regions: Western Mediterranean, Ionian Sea, Aegean Sea and Levantine Sea.

## Results

The developed CS methodological protocol and *CIGESMED for divers* website (http://cs.cigesmed.eu/) comprise:

- An educational module with simplified information regarding coralligenous assemblages, in order to ensure a basic understanding of these habitats by the diving community, answers to frequently asked questions that were identified during the field trials, and detailed guidelines for *in situ* data collection in five languages; English (Suppl. material 1), French (Suppl. material 2), Greek (Suppl. material 3), Italian (Suppl. material 4), and Turkish (Suppl. material 5).
- A multilingual data submission infrastructure, using an online web platform, where one can readily download the data-recording dive slates and subsequently upload the recorded information after each dive; the printable dive slates include visual guides and fill-in forms in English (Suppl. material 6), French (Suppl. material 7), Greek (Suppl. material 8), Italian (Suppl. material 9), and Turkish (Suppl. material 10) while the web version is also available in Spanish.

**Figure 1.**
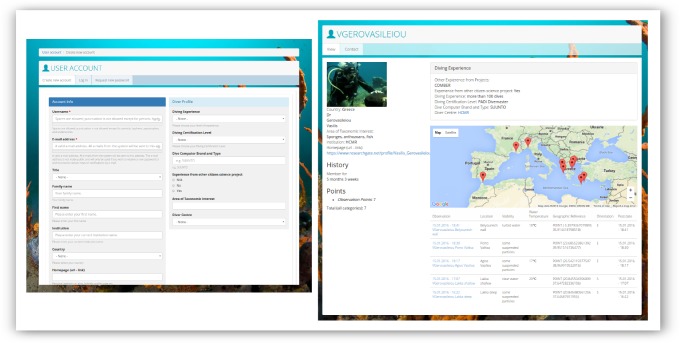
User account registration form of *CIGESMED for divers* website.

The website requires registration from the user in order to be able to submit, view and review data. During the initial registration process, users are requested to submit basic personal data (e.g. name, country of residence, affiliation) and information on their diving profile (i.e. diving experience and certification level), dive computer brand and type (for standardizing temperature data records), past experience from any other CS projects, area of taxonomic interest in cases of professional scientists and enthusiast naturalists, and dive centre (Fig. 1). Users are also able to choose among two data entry form options: (a) Standard taxonomic, that is ranking taxa in a standard phylogenetic order for educational/scientific reasons, or (b) a guided version, following the arrangement on the printed slate version, where different taxa are ranked in a way optimized to facilitate reporting *in situ*, according to experience gained from the field trials.

**Figure 2.**
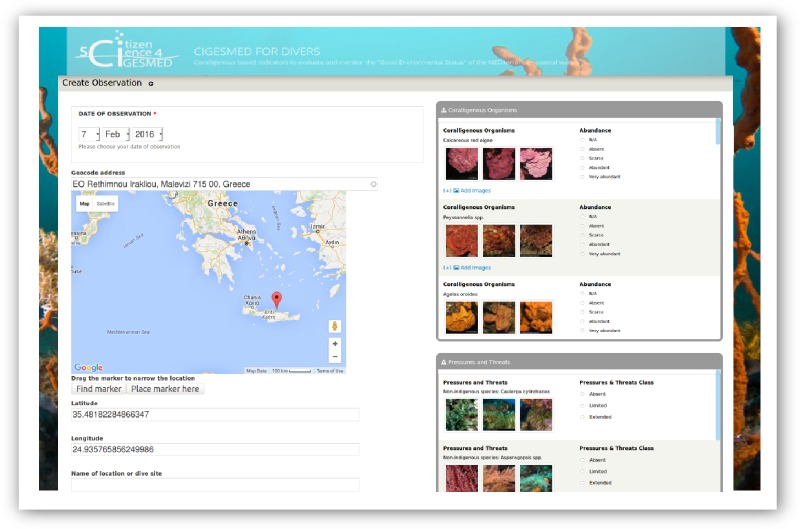
Observation form in the data submission platform of the *CIGESMED for divers* website.

The main data submission infrastructure (dive slate and web version) provides fields for reporting information relevant to the characterization of the site and the assessment of its coralligenous assemblages, organized in two sections: (a) basic data and parameters, and (b) ecological observations (Fig. 2). In the web version it is clearly stated that the information fields requested within those two sections are optional, i.e. at each recording event the participant is free to submit whichever data he/she was able to collect during the dive or he/she is willing to share. There are three compulsory fields, namely "Date of observation", "Geographic Reference" and "Observation depth (m)", marked with an asterisk (*). In several cases, images and explanatory texts are provided for certain fields (e.g. Thermocline’s Depth: At what depth did you feel a sharp decrease of temperature?) and their observation ranks. Online data submission is aided with tick boxes, selections from drop-down menus, free text fields, and an interactive map (Leaflet.js and Google Maps).

### Basic data and parameters

This section integrates typical geographic and topographic data along with several easily assessed abiotic environmental parameters which can allow a preliminary description of the site, as well as the development of data series for sites receiving multiple visits (Table 1). The location of the dive is reported in the online observation form through an interactive map which allows the user either to visually recognize the site and place a waypoint, or manually enter coordinates. Observation ranks of the topographic features are presented with symbols taken from the CIGESMED protocol for monitoring coralligenous (David et al. 2014). A command line process for underwater data reporting is provided in the guidelines.

### Ecological observations: reporting biodiversity and threats

This section of the CS platform focuses on: (a) the presence and relative abundance of typical conspicuous species of coralligenous assemblages and (b) the recording of pressures and imminent threats. A total of 23 taxa belonging to 7 taxonomic groups were included in the CS protocol (Table 2). The list is restricted to the minimum number of species possible, so as not to overwhelm citizen scientists with a large amount of information. The scope of this taxon list was not to be as comprehensive as possible but rather to include certain taxa that are characteristic of the various coralligenous facies, as well as a number of rare, protected and commercial species the reporting of which is scientifically interesting. Confirmation of the presence of coralligenous assemblages and their rough characterization were the main priorities considered. The taxa comprising the list are conspicuous and properly selected to assure: (a) easy identification underwater, (b) common presence in approachable coralligenous formations, (c) indication of a true coralligenous assemblage, and (d) potential presence in different regions of the Mediterranean basin, taking into account biogeographical heterogeneity. Therefore, species that may be characteristic of coralligenous bioherms but are difficult to spot or identify *in situ* by amateurs were omitted in favour of more conspicuous ones. On the other hand, the inclusion of a free-text field for reporting organisms not included in the basic list, allows more advanced users to upload additional records according to their best knowledge level. Information on the appropriate underwater recording of species, as well as their importance for the marine ecosystem are presented in the multilingual guidelines in a comprehensive manner.

**Figure 3.**
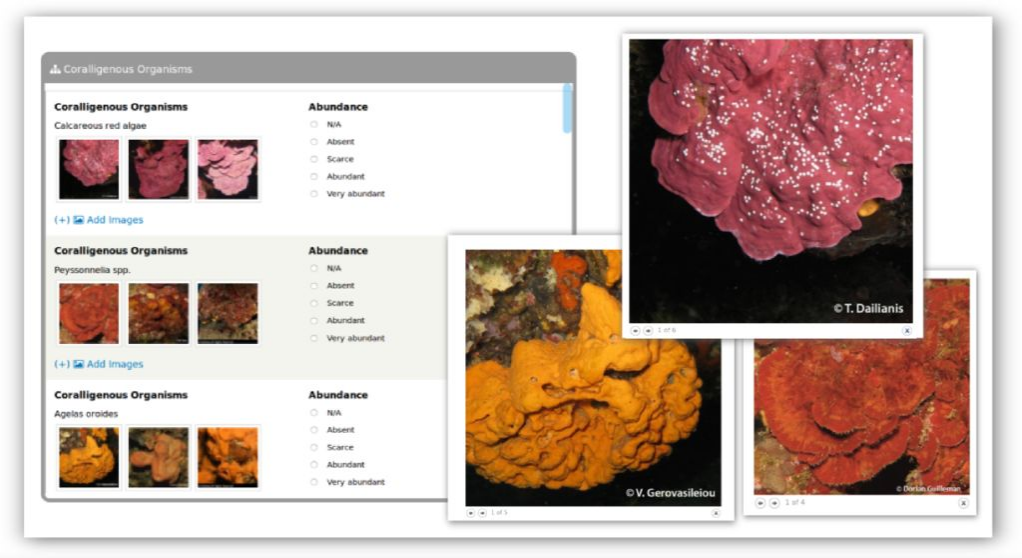
List of typical species of Mediterranean coralligenous assemblages on the *CIGESMED for divers* website, linked to galleries with representative photographs. Users have the ability to select pre-defined abundance classes and upload their own images for every listed taxon.

Typical species/taxa are presented in a list, accompanied by professional grade *in situ* photographs of the organisms from different Mediterranean areas, and linked to galleries providing additional photographic material (Fig. 3), covering as many viewing angles, morphologic and colour variations of the taxa as possible (ca. 100 images were assembled for this purpose). Semi-quantitative assessment of abundance is requested to be provided in size classes (N/A: Not available, Absent, Scarce, Abundant, Very abundant). Furthermore, the option of uploading photographs (i.e. Add Images) is available to the users and is encouraged as the recommended practice, since it provides the means to validate the diver’s observations. These photos are automatically added to the website’s Gallery following curation by the CS research team to avoid misidentifications.

**Figure 4.**
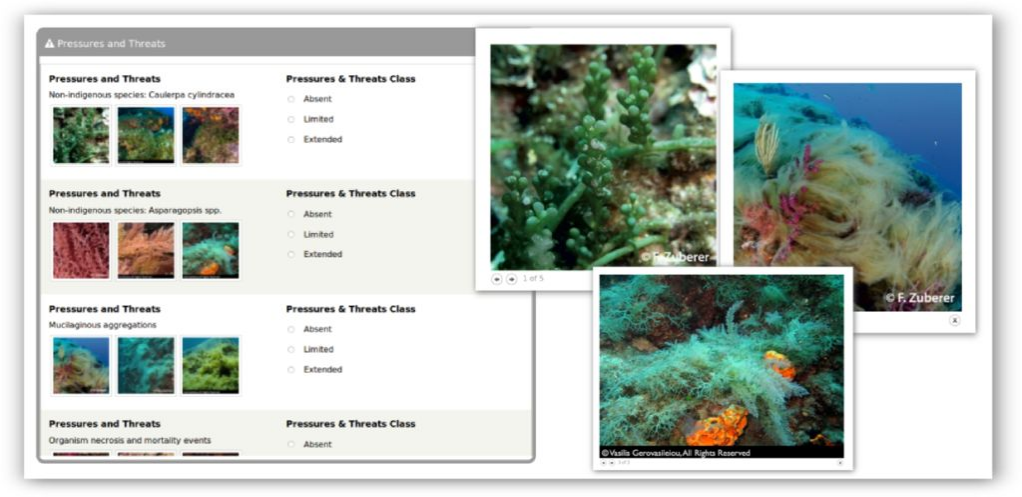
Reporting the intensity of pressures and threats on the *CIGESMED for divers* website.

A list of 10 types of pressures and threats commonly affecting coralligenous assemblages was included to the protocol (Table 3, Fig. 4), providing a basis for their reporting. For each category, citizen scientists have the option to quantify its intensity using 3 observation ranks (i.e. absent, limited, and extended). As is the case for species reporting, users also have the possibility to report other threat categories (i.e. Other Pressures & Threats) and any other type of observations (i.e. Other observations) in the respective free text fields, in order to allow commenting or providing any additional information.

### Data sharing policy and activities

**Figure 5.**
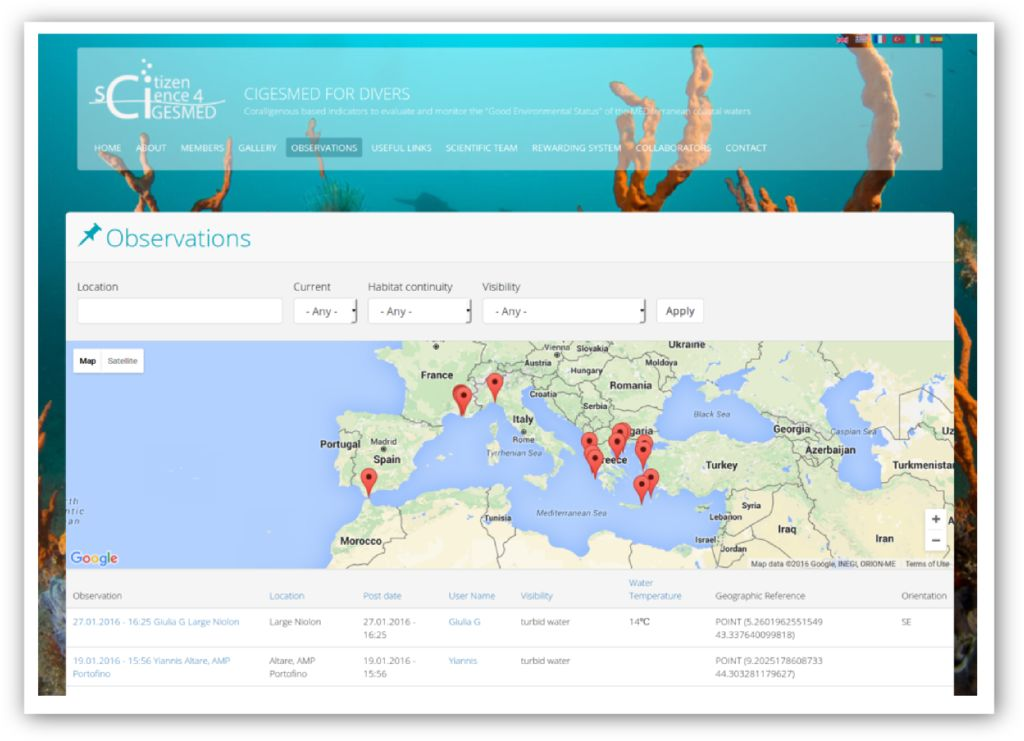
Accumulative map of uploaded observations for Mediterranean coralligenous assemblages on the *CIGESMED for divers* website.

All currently available data reports of coralligenous formations, their conspicuous species and threats are visibly marked on the accumulative map of the CS website (Fig. 5). Submitted data are open to public view and pass through a quality control process by the CIGESMED CS team before being reposited to global biodiversity databases (e.g. GBIF). As the website is social media-friendly, it provides out-of-the-box widgets allowing users to easily share observations or pages through popular social media (e.g. Twitter, Facebook, and LinkedIn).

### Enhancing motivation of participants

Active participation to *CIGESMED for divers*, as most citizen science initiatives, inherently rewards users with the notion of contribution to the exploration and the conservation of biodiversity, producing useful information out of a recreational activity, while at the same time increasing their knowledge of the marine environment. In addition to these motives, an online rewarding system is supported by the website, accrediting users according to their contribution level (number of observations submitted). As the number of observations is directly linked to diver’s experience and knowledge of the marine realm, 5 levels were assigned to male (*Theoi Halioi*) and female (*Oceanids* and *Nereides*) ancient Greek sea deities (Atsma 2011), linking Mediterranean natural and cultural heritage:

- 3 to 5 observations → Proteus/Nereid
- 6 to 9 observations → Glaucus/Naiad
- 10 to 14 observations → Nereus/Doris
- 15 to 29 observations → Oceanus/Tethys
- 30 observations and more → Poseidon/Amphitrite

The rewarding system can potentially encourage fans of online applications and social media but will also function as a potential metric for identifying enthusiast divers and local collaborators interested in supporting localized monitoring activities. In the online platform, all observations and uploaded photographs are linked to the user’s name and constitute intellectual property of the contributor, whilst free for use under a Creative Commons Attribution License (CC-BY). All participants and contributors, including dive and nature associations and clubs are accredited and, in certain cases, could be considered as co-authors in relevant scientific and mass media publications (see acknowledgements and author contributions sections).

### Smartphone applications

**Figure 6.**
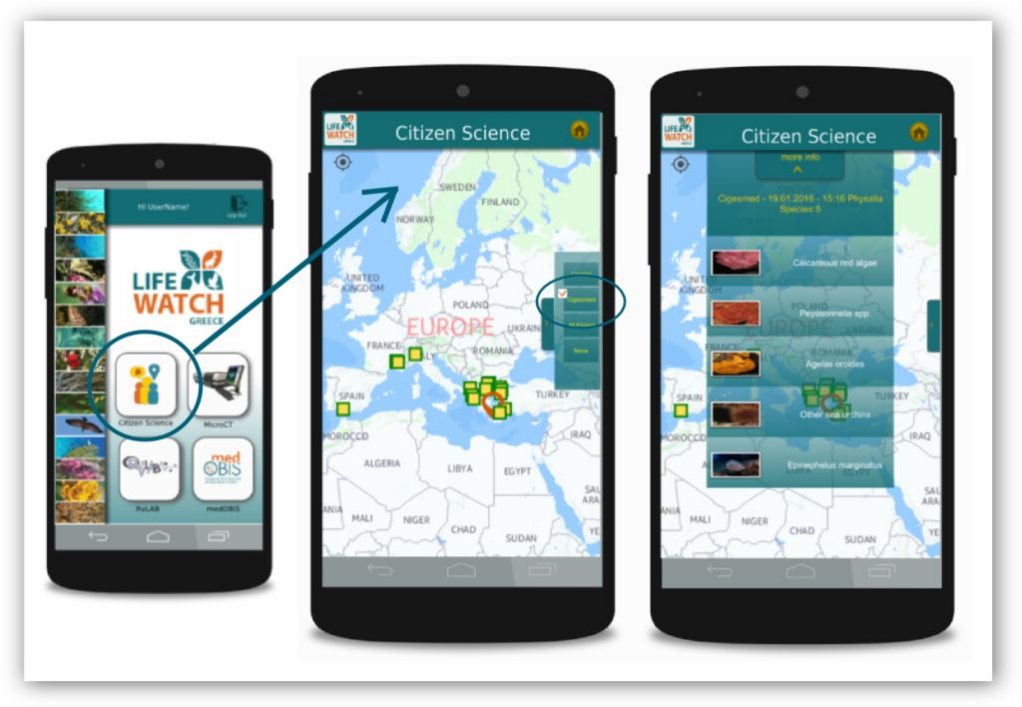
Interface of the smartphone application showcasing information regarding Mediterranean coralligenous assemblages and species obtained from *CIGESMED for divers* website. This is a “Citizen Science” mobile sub-application of the LifeWatchGreece infrastructure.

**Figure 7.**
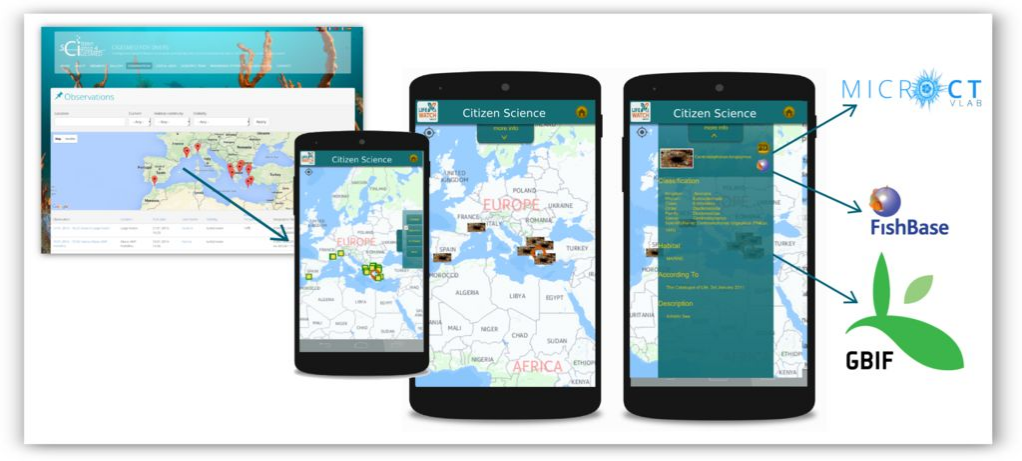
Interface of the smartphone application displaying the exchange of information between different applications of LifeWatchGreece infrastructure and global biodiversity databases.

The "Citizen Science" sub-application of the LifeWatchGreece mobile app aggregates data from different CS initiatives in order to support a simple and quick view process, assisting sharing of the obtained information. It is available for Android platforms (Mobile/Tablet) and receives data in JSON format from *CIGESMED for divers* website in real time. The application showcases a simplified version of the observations submitted to the "CIGESMED for divers" website on a map. Users are given the option to select and view details for each observation, such as the list of reported species (Fig. 6). By selecting a species, users can view its distribution, based on the registered data, as well as taxonomic and ecological information from global biodiversity databases like GBIF and FishBase (Fig. 7). Furthermore, species records are connected to the Micro-CT_vlab_sub-application of LifeWatchGreece mobile app where users can explore relevant Micro-CT datasets (i.e. series of images using X-Ray micro-computed tomography) through images, videos and 3D models that can be virtually manipulated, dissected and rotated using online tools (see Keklikoglou et al. 2016, this special collection).

## Discussion

The engagement of citizens in marine research is particularly important, since professional scientific activities are often restricted by available financial and human resources (Thiel et al. 2014). Moreover, research projects often follow a confined implementation time schedule, after which research focus shifts to different subjects. The present CS initiative aims to maximize the geographical and temporal capacity of CIGESMED project. Although the core research activities of CIGESMED are localized in France, Greece and Turkey, the flexible and multilingual character of the CS protocol and website infrastructure enable their implementation on a Pan-Mediterranean scale. Our long-term goal is to establish a user-friendly and scientifically meaningful tool for the mapping, ecological status assessment and monitoring of coralligenous assemblages across the Mediterranean.

The objectives of this CS initiative extend beyond single-point data collection by providing a way to monitor coralligenous assemblages and assess potential degradation through the creation of long-term data series in sites receiving multiple visits (e.g. Marine Protected Areas). The inclusion in the protocol of several rare, protected, and commercial species (e.g. the over-exploited red coral), could further assist in their monitoring and conservation. This is particularly important in countries where research funds dedicated to environmental assessment and monitoring of the marine environment are low or non-existent; under such circumstances the contribution of skilled citizen scientists could critically enable the persistent collection of qualitative or semi-quantitative data (Bramanti et al. 2011).

The increasingly important role that active volunteers can play in reporting of the presence and spread of marine allochthonous species has been recently pointed out (Scyphers et al. 2014, Zenetos et al. 2013), along with the necessity for visible centralized data submission and dissemination platforms that can drive citizen participation. In this context, an array of established citizen-monitored sites can prove valuable to swift alien biodiversity records, especially if their online infrastructure is directly linked to relevant data aggregation channels (e.g. ELNAIS).

Citizen-science approaches are generally acknowledged as a main pathway to assist the progress towards the upscaling of ecological data to the global level (Edgar et al. 2016). In this context, LifeWatchGreece research infrastructure (ESFRI) provides the means, expertise and the continuous technical support for the maintenance and development of the electronic platform behind the pilot *CIGESMED for divers* CS project for the coralligenous habitats in the Mediterranean. It also provides the electronic infrastructure needed for: (a) the data management in order to be harvested by the international aggregators, such as OBIS and GBIF; (b) the appropriate electronic infrastructure in order to analyze the data and interpret the results, in the form of the virtual laboratory RvLab (Varsos et al. 2016, this special collection).

Sharing data is one of the main objectives of CIGESMED. However, data assembled by non-experts should go through quality control before being used in scientific analyses or reposited to global biodiversity databases (Arvanitidis et al. 2011, Thiel et al. 2014). The list of organisms included in the present CS protocol include conspicuous species that are relatively easy to correctly identify. These include species that serve as ecosystem engineers and define coralligenous assemblages, such as coralline algae, sponges and gorgonians, as well as motile fauna that is also typical of this habitat type (e.g. *Anthias anthias*). The provision of detailed guidelines, easy-to-follow directions, high quality photos and simplified dive-slate versions of the CS protocol offer essential tools for data standardization and minimization of observer bias. Additionally, the online data submission platform encourages divers to upload their photos for each species record, thus facilitating quality control. Each observation is associated to the user’s name, giving the opportunity to the CS research team to access the profile features of the diver (e.g. participation in other CS projects, diving experience, taxonomic expertise for scientific divers and naturalists) or even to contact him/her directly in exceptional cases (e.g. outstanding records, reporting of severe habitat degradation). For this reason, we have established groups of "country managers" among the involved researchers, acting as focal points responsible for providing support to users, managing and evaluating observations from different geographic regions, performing quality control and contacting divers in their language when needed.

Following screening and quality control, the data collected by the *CIGESMED for divers* project will be further used to: (a) calculate the values of indices for the assessment of the "good environmental status" (GES) of the habitat, as required by the Marine Strategy Framework Directive (MSFD). For this purpose, only indices requiring a minimum of information will be used, such as those proposed under the Descriptor 6 of the MSFD (sea-floor integrity) (Rice et al. 2012); (b) the calculation of the values of the Essential Biodiversity Variables (EBVs) (Pereira et al. 2013) that can be applied to coralligenous habitats. Such variables can be defined at the scale of community richness and diversity.

During the field trials and discussions with participants, their associations and diving clubs, as well as national MPA authorities, it became evident that a considerable proportion of divers, especially in the eastern Mediterranean, were not aware of the existence and importance of coralligenous assemblages despite the fact that these formed their preferred diving spots. The educational module of the project (i.e. guidelines and website) combined with the experience gained from the participation of divers directly raises public awareness. Experience from the field trials and past CS projects showed that knowledge exchange between citizen scientists and researchers promotes trust and facilitates application of marine conservation schemes (Arvanitidis et al. 2011, Thiel et al. 2014). Participation to monitoring CS projects can itself have a positive effect on recreational participants’ knowledge level and environmental awareness (Branchini et al. 2014). Establishing communication with users is also important for the optimization of the protocol and reporting of possible bugs in the website.

Charismatic, flagship species (e.g. gorgonians, the dusky grouper) that thrive in coralligenous bioherms can raise the interest of recreational divers and greatly increase their awareness for the habitat as a whole, or their willingness to invest time for data recording. For example, in a recent citizen science project regarding the presence of red coral along the coasts of Italy, Bramanti et al. (2011) reported that 80% of the participants selected their diving location based on the presence of red coral. Furthermore, many volunteers dedicated several dives to estimate colony density and extent of mortality, an indication of the willingness to devote valuable diving time to activities related to conservation.

A recent review study by Thiel et al. (2014) on the role of citizen scientists in marine research showed that the motives of participating people vary, with personal satisfaction and public recognition being the most frequent ones (85% of cases) along with knowledge gaining (52%). In the present CS project these two motives effectively define the profile of the targeted citizen scientists – enthusiast divers – and are directly addressed through the design of the developed protocol, data sharing and data publication policies of CIGESMED. To this end, all participants will be fully acknowledged and in certain cases included as co-authors in resulting communications to scientific or general media. Divers with a high level of input, according to the online rewarding system, could further contribute as local collaborators supporting joined missions and monitoring activities.

A series of joint activities organized by the CIGESMED CS researchers, with selected motivated diving associations and MPA management authorities have been scheduled in Greece (e.g. Korinthiakos Gulf and National Marine Park of Zakynthos), France (e.g. Atelier Bleu - CPIE Côte Provençale, Ailes Sportives Airbus Helicopters, Septentrion Environnement), and Turkey (e.g. Derin and Doğa Diving Center, Engin Sea and Nature Sports Center). Those include photo-sampling of permanent quadrats, recording of environmental data (e.g. through the deployment of loggers) and recording of threats and impacts on a scheduled and systematic basis. Joint missions will further include training of the involved divers and demonstrating the proposed activities using the developed CS protocol and e-infrastructure. Furthermore, online photo-contests are to be periodically scheduled and high-quality photographs uploaded by users will be screened on the homepage of the website or could be used in future scientific and educational publications, always acknowledging the photographer. Combined with the *CIGESMED for divers* CS protocol, these activities, are expected to enhance the creation of long-term data series, increase public awareness and establish communication and knowledge exchange on a more trust-worth and permanent basis.

## Supplementary Material

Supplementary material 1 CIGESMED for divers – Citizen Science for CIGESMED Data type: Guidelines
Brief description: Guidelines for the implementation of CIGESMED Citizen Science protocol for the study and monitoring of Mediterranean coralligenous assemblages.
File: oo_82405.pdf Gatti G., Thierry de Ville d’Avray L., David R., Dimitriadis C., Gerovasileiou V., Dailianis T., Sini M., Salomidi M., Dogan A., Issaris Y., Cinar M.E., Koutsoubas D., Arvanitidis C., Feral J-P.

Supplementary material 2 CIGESMED pour les plongeurs – Les sciences participatives pour CIGESMED Data type: Guidelines
Brief description: Guidelines for the implementation of CIGESMED Citizen Science protocol for the study and monitoring of Mediterranean coralligenous assemblages.
File: oo_82406.pdf Gatti G., Thierry de Ville d’Avray L., David R., Dimitriadis C., Gerovasileiou V., Dailianis T., Sini M., Salomidi M., Dogan A., Issaris Y., Cinar M.E., Koutsoubas D., Arvanitidis C., Feral J-P.

Supplementary material 3 CIGESMED για δύτες, πολίτες-επιστήμονες για το πρόγραμμα παρακολούθησης των κοραλλιγενών οικοτόπων Data type: Guidelines
Brief description: Guidelines for the implementation of CIGESMED Citizen Science protocol for the study and monitoring of Mediterranean coralligenous assemblages.
File: oo_82408.pdf Gatti G., Thierry de Ville d’Avray L., David R., Dimitriadis C., Gerovasileiou V., Dailianis T., Sini M., Salomidi M., Dogan A., Issaris Y., Cinar M.E., Koutsoubas D., Arvanitidis C., Feral J-P.

Supplementary material 4 CIGESMED per i subacquei - Citizen science per CIGESMED Data type: Guidelines
Brief description: Guidelines for the implementation of CIGESMED Citizen Science protocol for the study and monitoring of Mediterranean coralligenous assemblages.
File: oo_82410.pdf Gatti G., Thierry de Ville d’Avray L., David R., Dimitriadis C., Gerovasileiou V., Dailianis T., Sini M., Salomidi M., Dogan A., Issaris Y., Cinar M.E., Koutsoubas D., Arvanitidis C., Feral J-P.

Supplementary material 5 Dalgıçlar için CIGESMED – CIGESMED için Vatandaş Bilimi Data type: Guidelines
Brief description: Guidelines for the implementation of CIGESMED Citizen Science protocol for the study and monitoring of Mediterranean coralligenous assemblages.
File: oo_82411.pdf Gatti G., Thierry de Ville d’Avray L., David R., Dimitriadis C., Gerovasileiou V., Dailianis T., Sini M., Salomidi M., Dogan A., Issaris Y., Cinar M.E., Koutsoubas D., Arvanitidis C., Feral J-P.

Supplementary material 6 CIGESMED for divers – Citizen Science for CIGESMED Data type: Slates
Brief description: Printed fill-in forms for underwater observation for the implementation of CIGESMED Citizen Science protocol for the study and monitoring of Mediterranean coralligenous assemblages.
File: oo_82412.pdf Gatti G., David R., Dimitriadis C., Doğan A.

Supplementary material 7 CIGESMED pour les plongeurs – Les sciences participatives pour CIGESMED Data type: Slates
Brief description: Printed fill-in forms for underwater observation for the implementation of CIGESMED Citizen Science protocol for the study and monitoring of Mediterranean coralligenous assemblages.
File: oo_82413.pdf Gatti G., David R., Dimitriadis C., Doğan A.

Supplementary material 8 CIGESMED για δύτες, πολίτες-επιστήμονες για το πρόγραμμα παρακολούθησης των κοραλλιγενών οικοτόπων Data type: Slates
Brief description: Printed fill-in forms for underwater observation for the implementation of CIGESMED Citizen Science protocol for the study and monitoring of Mediterranean coralligenous assemblages.
File: oo_82419.pdf Gatti G., David R., Dimitriadis C., Doğan A.

Supplementary material 9 CIGESMED per i subacquei - Citizen science per CIGESMED Data type: Slates
Brief description: Printed fill-in forms for underwater observation for the implementation of CIGESMED Citizen Science protocol for the study and monitoring of Mediterranean coralligenous assemblages.
File: oo_82420.pdf Gatti G., David R., Dimitriadis C., Doğan A.

Supplementary material 10 Dalgıçlar için CIGESMED – CIGESMED için Vatandaş Bilimi Data type: Slates
Brief description: Printed fill-in forms for underwater observation for the implementation of CIGESMED Citizen Science protocol for the study and monitoring of Mediterranean coralligenous assemblages.
File: oo_82417.pdf Gatti G., David R., Dimitriadis C., Doğan A.

## Figures and Tables

**Table 1.** Basic information requested in the data submission platform of the on the *CIGESMED for divers* protocol (* compulsory field).

**Observation fields**	**Remarks, explanatory texts and observation ranks**
Date of observation*	Day/Month/Year
Geographic reference*	Coordinates in Decimal Degrees (based on Google WGS 84 Web Mercator coordinate system) and/or selection on interactive map
Name of location or dive site	If there is any
Water temperature at predefined depths (e.g. 0, 10, 20, 30, 40 m)	Temperatures in Celsius degrees (^o^C)
Thermocline’s depth	At what depth did you feel a sharp decrease of temperature?
Water temperature at the observation depth	Temperature in Celsius degrees (^o^C)
Maximum dive depth	In meters (m)
Observation depth*	In meters (m); this field is required
Current	Observation ranks: none, weak, strong
Visibility	Observation ranks: clear water, some suspended particles, turbid water
Vertical extent of the habitat	Min and max depth of observed coralligenous formations
Horizontal extent of the habitat	Observation ranks: less than 5 m, 5-10 m, 10-20 m, more than 20 m
Habitat continuity	Observation ranks: isolated patch, discontinuous, continuous
Slope	Observation ranks: vertical, sloping, horizontal, overhanging
Rugosity	Observation ranks: tiny, small, medium, large
Orientation of the coralligenous site	Observation ranks: N, NE, E, SE, S, SW, W, NW

**Table 2.** List of coralligenous biota examined in the current CS initiative (* protected species).

** RHODOPHYTA **
Calcareous red algae (e.g. *Lithophyllum* spp. and *Mesophyllum* spp.)
*Peyssonnelia* spp.
** PORIFERA **
*Agelas oroides*
*Axinella* spp. *
*Cliona* spp.
** ANTHOZOA **
*Eunicella cavolini* *
Eunicella singularis*
*Paramuricea clavata* *
*Leptogorgia sarmentosa*
Savalia savaglia*
Scleractinians (e.g. *Leptopsammia pruvoti*, *Polycyanthus muellerae*, *Madracis pharensis*, *Hoplangia durotrix*, and *Phyllangia americana mouchezii*) *
*Corallium rubrum* *
** CRUSTACEA **
*Homarus gammarus*
*Palinurus elephas*
*Scyllarides latus* *
** BRYOZOA **
*Myriapora truncata*
Other erect bryozoans (e.g. *Adeonella* spp., *Smittina* spp., *Pentapora fascialis*, *Reteporella* spp.)
** ECHINODERMATA **
*Centrostephanus longispinus* *
Other sea urchins (e.g. *Arbacia lixula*, *Echinus melo*, *Paracentrotus lividus*, *Sphaerechinus granularis*)
** PISCES **
*Anthias anthias*
*Epinephelus marginatus* *
*Scorpaena* spp.
Shark egg cases (e.g. *Scyliorhinus canicula*)

**Table 3.** List of pressures and threats included in the protocol

Non-indigenous species: *Caulerpa cylindracea*
Non-indigenous species: *Asparagopsis* spp.
Mucilaginous aggregations
Organism necrosis and mortality events
Sedimentation on coralligenous biota
Diver recklessness
Fishing gear
Litter
Anchoring
Urban waste sources nearby
Other pressures & threats (free text)

## Project

CIGESMED: Coralligenous based Indicators to evaluate and monitor the "Good Environmental Status" of the MEDiterranean coastal waters (coordination IMBE, CNRS, France)

## Project

CIGESMED: Coralligenous based Indicators to evaluate and monitor the "Good Environmental Status" of the MEDiterranean coastal waters (coordination IMBE, CNRS, France)
