# Supplementary material for: CIGESMED for divers: Establishing a citizen science initiative for the mapping and monitoring of coralligenous assemblages in the Mediterranean Sea
Source: Biodivers Data J. 2016 Nov 1;(4):e8692. doi: 10.3897/BDJ.4.e8692 (PMC5136673; doi:10.3897/BDJ.4.e8692)
Supplement: Supplementary material 1 — CIGESMED for divers – Citizen Science for CIGESMED [file biodiversity_data_journal-4-e8692-s001.pdf]

http://cs.cigesmed.eu

www.cigesmed.eu

CIGESMED for divers – Citizen Science for CIGESMED

What is "coralligenous"?

What is commonly called coralligenous is, actually, a complex seascape (i.e. a submerged landscape) developing in dim light conditions, which is present exclusively in the Mediterranean Sea. Coralligenous habitat is mainly created by calcified red algae, which are constructing reef-like structures for thousands of years over marine rocky bottoms. In this respect coralligenous formations are considered to be the Mediterranean equivalent of the tropical coral reefs. At the same time, some other organisms- which are called 'eroders' - such as sponges of the genus *Cliona* or sea urchins - pierce and consume the calcareous substrate and thus contribute to increase the structural complexity of the coralligenous formations. This calcareous substrates also support several long-living species (sponges, gorgonians, bryozoans, corals), which are contributing to the structuring of the coralligenous habitat. Finally, a large number of invertebrates (such as nudibranchs, crustaceans, ascidians, echinoderms, molluscs) and fishes are associated with coralligenous habitats, a fact which further contributes toward the characterization of the coralligenous habitat as a unique biodiversity hotspot.

Why do we observe coralligenous habitats?

They are unique habitats worldwide and among the richest and most beautiful seascapes to observe during scuba diving. Thanks to their complexity, they shelter a great number of ecologically, aesthetically and commercially valuable species, some of which are also protected by National and International Laws. Coralligenous habitats are often threatened by the human behaviour. Intense anchoring, irresponsible diving, (over)fishing, litter dumping, alongside with sea surface warming (due to climatic change) and alien species invasions, are among the main threats which can induce negative effects on the health status of coralligenous habitats.

By participating into the *CIGESMED for divers* – Citizen Science for CIGESMED project you contribute to the exploration and the conservation of the coralligenous habitats and the marine environment, while at the same you can increase your knowledge about the marine biodiversity.

What do we observe?

1. <u>Some general information about the site</u>

Water temperature at observation depth:

The **temperature of the water** is an essential parameter for species survival and it is important to have such information **from the depth of the observation**.

At what depth you met colder water?	m / never

This depth corresponds to the lower limit of the thermocline, the zone in which an abrupt decline of the water **temperature** is present (transition from shallow (warmer) to deeper (colder) waters). Sometimes, this limit can be found deeper than it is normally expected due to the occurrence of unusual heat waves. As a result, organisms usually living in colder conditions come in contact with warmer water. If this phenomenon persists, it can cause partial or extensive mortality of several species and particularly of gorgonians and sponges.
Please fill in the boxes by indicating the water temperature at the observation depth and the lower limit of the thermocline (At what depth you met colder water?) in metres or mark the option 'never' if you did not meet the thermocline at all.

Observation depth:	Current	Visibility
	None ☐ Weak ☐ Strong☐	Clear water ☐ Some particles ☐ Turbid ☐

The **depth** is an essential factor in determining the distribution of marine species and the composition of coralligenous habitats. Please fill in the depth (in metres) where the observation was carried out.

At the depth of the observation, information about current intensity and visibility integrate the general description of the site. For the **current**, a multiple choice among three categories is proposed: **none**, **weak**, **strong**. Concerning the **visibility**, again, three classes are considered: **clear water**, **turbid water** and an intermediate category called "**some** (suspended) **particles**".

Please, tick the box corresponding to the observed current intensity and visibility.

2. <u>Site and habitat characteristics</u>

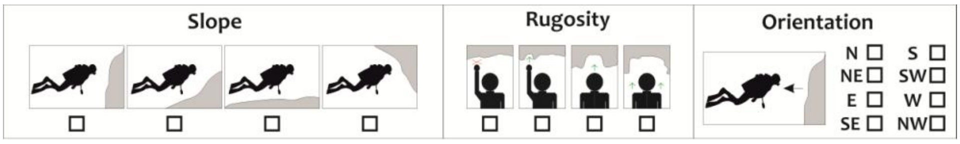

Substrate slope, rugosity and orientation, along with the depth, are among the major factors in determining the composition of coralligenous habitats.

Organisms are exposed to different light intensity and sediment deposition according to the **slope** of the substrate where they are found on. Four categories of substrate slope can be considered: **vertical**, **sloping**, **horizontal** and **overhanging**.

Rugosity corresponds to rocky bottom's irregularity: the more irregular the substrate is, the more complex it is. A practical way to categorize rugosity on the basis of anatomic references can be as

follows. Crevices and holes which are: 1) **too small to allow diver's fist to enter therein**, or 2) **large enough to allow** diver's **fist**, 3) **head** or 4) **shoulders** to enter. In most cases, it is difficult to find only one kind of crevice: in such a case, the larger size should be chosen, because it is assumed that it may contain smaller crevices.

The **orientation** is the direction that the substrate is facing to. In order to measure it, it is necessary to face the wall and to mark the direction indicated by the imaginary arrow coming out from the wall. Obviously, horizontal substrates do not have any orientation.

Please tick the box which corresponds to the slope, to the larger crevices and to the substrate orientation that you observed.

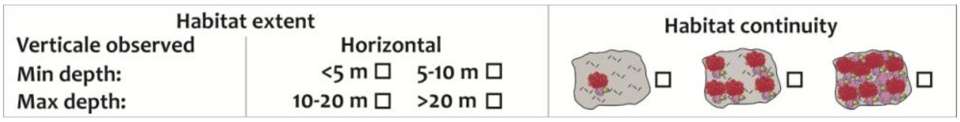

The whole vertical extent of the coralligenous habitat cannot be observed in all cases, since it may often largely exceed the maximum depth that you reached during your dive. For this reason, the field "**observed vertical extent of the habitat**" refers to the minimum and maximum depth (in metres) at which you observed the coralligenus habitat and not to the maximum depth that coralligenous habitats actually reach. In other words, you can estimate the depth reached by the coralligenous habitat below you, or you can simply use the maximum depth that you reached during your dive. The abovementioned rationale applies also for the case of the minimum depth.

The **horizontal extent of the habitat** refers to the horizontal area of the wall that the coralligenus habitat is covering at the site that you are observing. A multiple choice among distance intervals is proposed: **<5 m**, **5-10 m**, **10-20 m** or **>20 m**. Please check the box corresponding to the observed extent.

The **habitat continuity** describes whether coralligenous is limited to a single isolated patch (surrounded by sediment or bare rock for example), if it is discontinuous (only few uncovered areas) or continuous (no remarkable interruptions) (figures are ordered from the left to the right side of the box, respectively). Please tick the box corresponding to the observed situation.

3. Pressures affecting the habitat

All pressures, both of human or natural origin, can threaten species survival and habitat stability. In some cases it is difficult to prevent or to restore the negative effects of the pressures (e.g. the case of invasive species). However, the monitoring of the pressures can assist in their management and in the reduction of the negative effects that they induce (e.g. controlled anchoring, limited fishing activity, responsible recreational diving, etc.).

An **estimation of the impact of the pressures** based on your perception can be obtained during your dive. Hence, you can evaluate the impact of each pressure (that are presented below), according to the following classes: **0 = absent; + = limited; ++ = extended.**

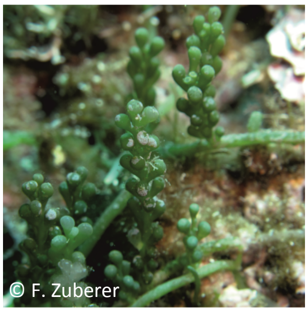

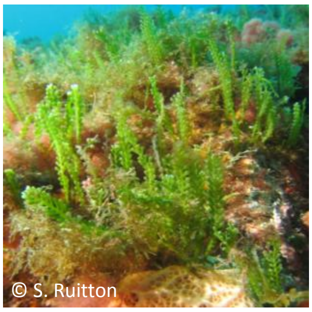

Caulerpa cylindracea

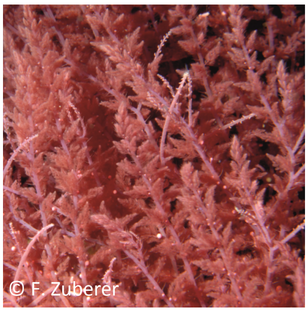

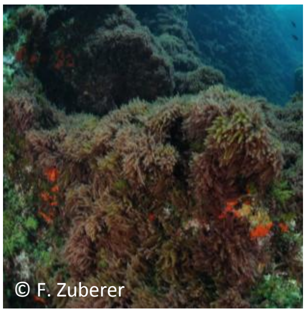

Asparagopsis spp.

The green alga *C. cylindracea* and the algae of the genus *Asparagopsis* have two communal characteristics: they are **non-indigenous**, which means that they are not of Mediterranean origin (tropical species that were introduced and established in the Mediterranean Sea); and they are **invasive**, which means that they can spread everywhere and outcompete the indigenous organisms. Since local biodiversity and habitat stability are threatened by such species, it is crucial to monitor their presence and abundance (rough estimation).

More photos: *C. cylindracea*, *Asparagopsis* spp.

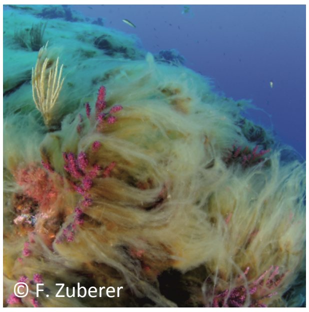

Mucilaginous aggregates

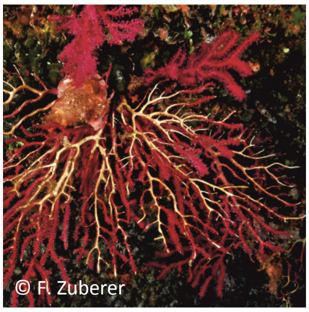

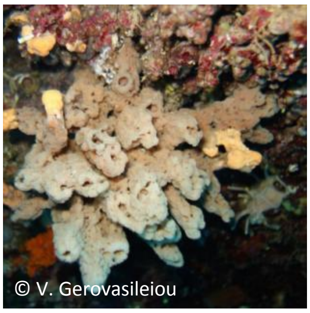

Necrosis/mortality events

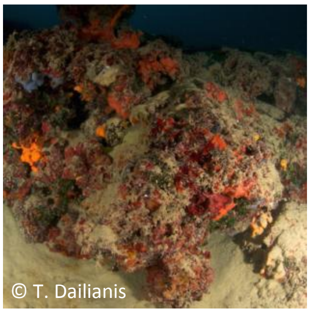

Sedimentation

Mucilaginous aggregates are composed by microscopic algae, which produce some substances responsible for their characteristic yellowish filamentous-like appearance. When some particular conditions of temperature and water circulation establish, mucilaginous aggregates may develop tremendously, totally covering the bottom and literally suffocating benthic organisms.

The presence of mucilaginous aggregates, along with the presence of exceptionally high water temperature for long periods, can cause partial or total **necrosis** (the death of a living tissue) of the

organisms, or even mass **mortality events** of entire populations (all the organisms belonging to the same species).

Intense **sedimentation** may be due to natural causes, for example heavy rainfalls, or to human activities, such as constructions, mining and treated water dumping in coastal areas. Whatever the origin, the excessive deposition of sediment over rocky substrates leads to organisms' burial and habitat degradation.

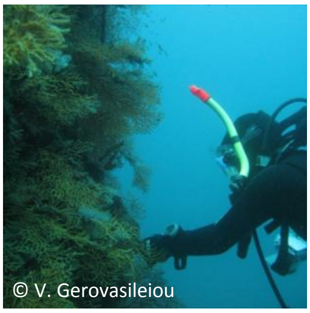

Divers recklessness

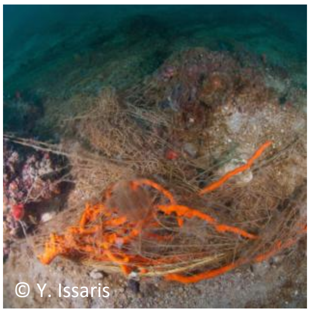

Fishing gear

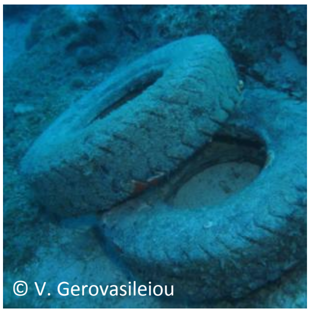

Litter

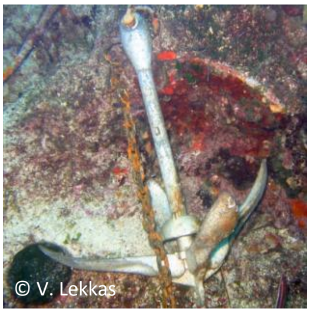

Anchoring

Litter of different nature, abandoned **fishing gears** and **anchoring** can locally cause serious damage to coralligenous habitats.

Also **divers' recklessness** may have negative consequences on coralligenous habitats. Inattentive divers may damage the organisms by touching them with their body or fins. Among others, the erect calcified bryozoans are particularly sensitive to mechanical damages: for this reason they are considered as indicators of the diving frequency and intensity that an area hosts. Looking at the cliff toe, the presence of broken individuals or entire bryozoans' and gorgonians' colonies suggests that the site is frequented by inattentive divers.

4. The species

The following list includes a limited number of species which were selected according to: 1) the role that they play in coralligenous habitats (e.g. builders, eroders, seascape structuring), 2) their regularity of appearance in these habitats (typical organisms of coralligenus habitats) and 3) their protection status (protected by National or International Laws). Information about their presence and abundance (rough estimation) enables for the description of the habitats and the assessment of their health status.

In order to **estimate the abundance** of each species (or group of species), the following classes should be considered: **0 = absent; + = scarce; ++ = abundant; +++ = very abundant**. Trust your judgement!

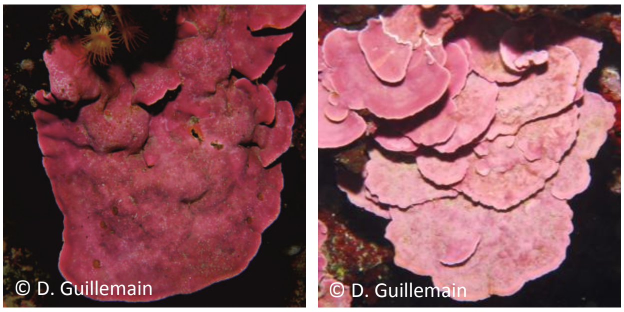

Calcareous red algae

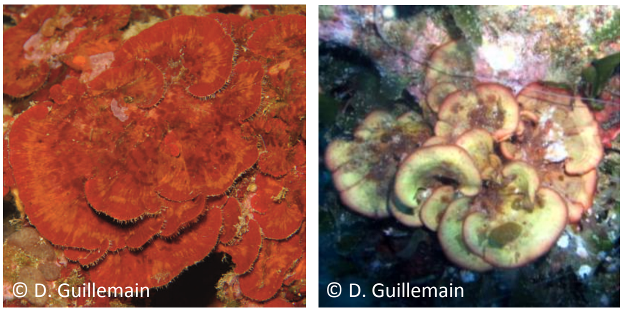

Peyssonnelia spp.

These red algae are the main representatives of coralligenous flora. **Calcareous red algae**, *Lithophyllum stictaeforme/cabiochiae* and *Mesophyllum expansum* in particular, are the main builders of the calcareous substrate. The algae belonging to the genus ***Peyssonnelia*** include both calcified (*Peyssonnelia rosa-marina*) and non-calcified (*Peyssonnelia squamaria*) species.

More photos: *L. stictaeforme/cabiochiae*, *M. expansum*, *P. rosa-marina*, *P. squamaria*.

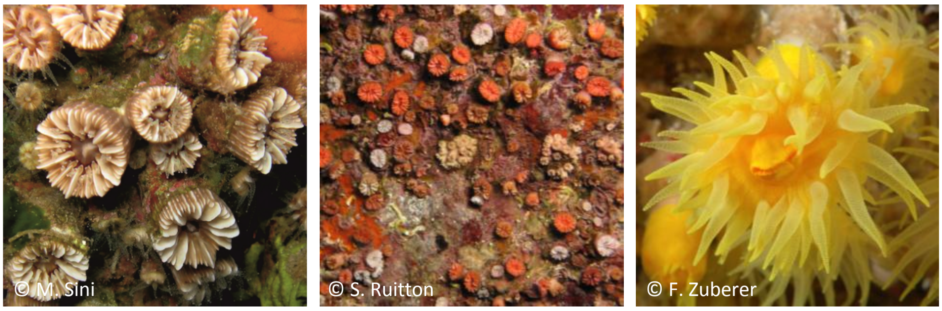

Scleractinians

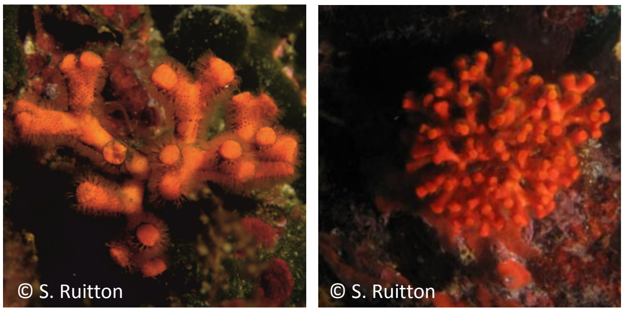

Myriapora truncata

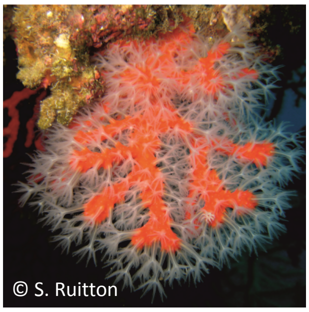

Corallium rubrum

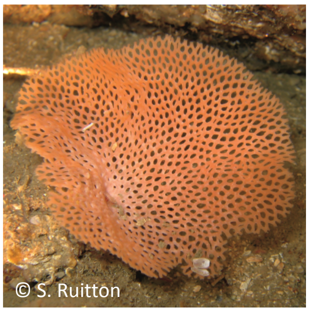

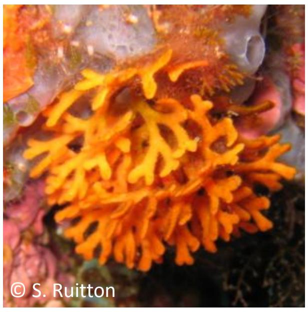

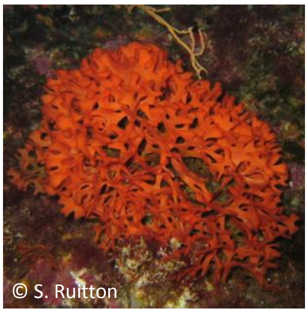

Other bryozoans

Scleractinians and bryozoans (***Myriapora truncata*** and **others**), producing a calcareous skeleton, are the secondary builders of coralligenous calcareous substrate. Bushy bryozoans are brittle and they can easily be damaged in case of mechanical impact: this is the reason why they are considered indicators of diving frequency on a particular place. Other bryozoans but *M. truncata* are also used as indicators of pollution, since they are sensitive to this pressure.
Not to be confounded with *M. truncata*, the red coral, despite its name, is not really a coral, but it is the only gorgonian that produce a calcareous skeleton. Thus, it can be considered among the secondary builders of coralligenous substrate. ***Corallium rubrum*** was recently included in the IUCN Red List of Threatened Species as an endangered species.

More photos: Scleractinians - *Caryophyllia (Caryophyllia) smithii*, *Caryophyllia inornata*, *Hoplangia durotrix*, *Leptopsammia pruvoti*, *Madracis pharensis*, *Phyllangia mouchezii*, *Polycyathus muellerae*. *M. truncata*. *C. rubrum*. Other bryozoans - *Adeonella calveti*, *Pentapora fascialis*, *Smittina cervicornis*.

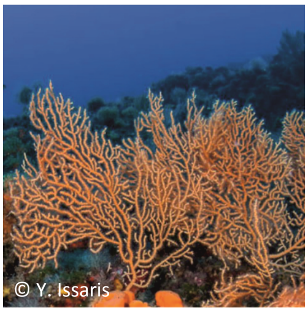

Eunicella cavolini

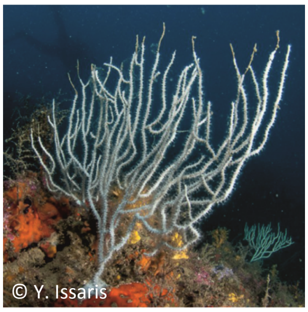

Eunicella singularis

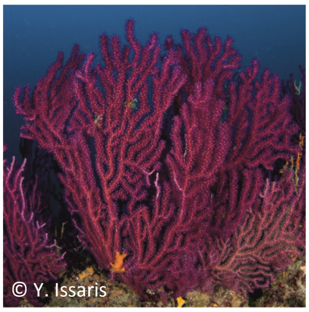

Paramuricea clavata

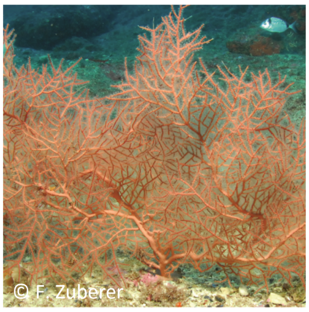

Leptogorgia sarmentosa

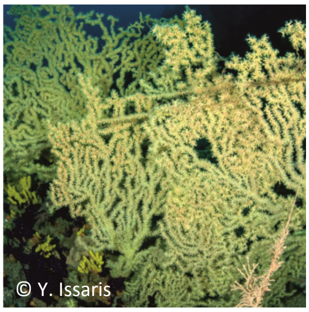

Savalia savaglia

Among the organisms that structure coralligenous seascapes, these tree-shaped species are definitely the most remarkable ones. Yellow (***Eunicella cavolini***), white (***Eunicella singularis***) and purple gorgonians (***Paramuricea clavata***) are the most common. ***Leptogorgia sarmentosa***, the orange gorgonian, prefers turbid waters, so it is considered as an indicator of turbidity.
Although ***Savalia savaglia***, the bushy anemone, looks like gorgonian species, it does not belong to this group of organisms. Actually, it is more closely related to anemones and corals, whereas it is rare and it can be found in deep water. Reporting of this species is important to assess its distribution.

Recently, *P. clavata* was included in the IUCN Red List of Threatened Species as a vulnerable species, and *E. cavolini*, *E. singularis* and *S. savaglia* as near threatened species.

More photos: *E. cavolini*, *E. singularis*, *P. clavata*, *L. sarmentosa*, *S. savaglia*.

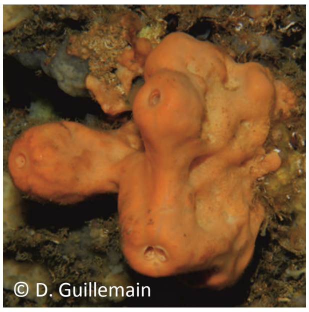

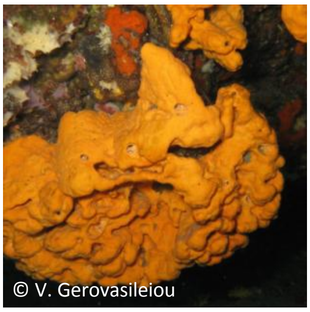

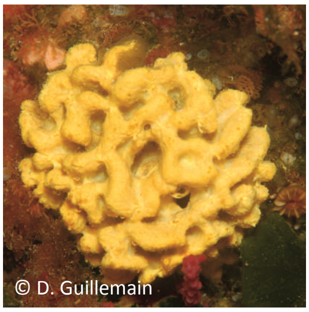

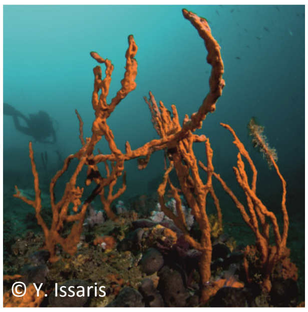

Agelas oroides *Axinella* spp.

The number of sponges that can be observed in coralligenous reef is huge. Thus, representation of this group of species was restricted to the most widespread ones across the Mediterranean Sea: ***Agelas oroides*** and the species of the genus ***Axinella*** (*Axinella damicornis, A. verrucosa, A. polypoides* (protected)).

More photos: *A. oroides*, *A. damicornis*, *A. verrucosa*, *A. polypoides*.

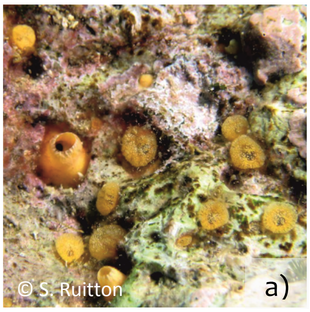

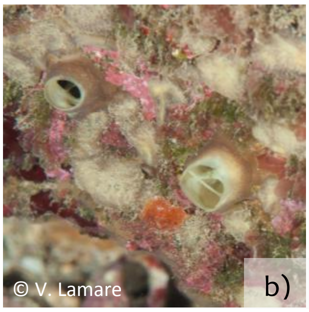

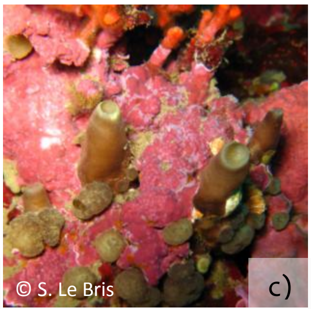

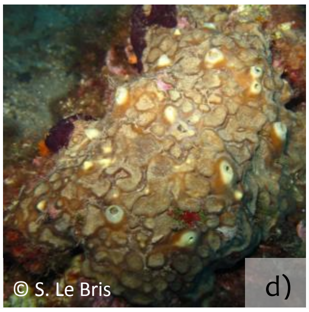

Cliona spp.

The sponges of the genus ***Cliona*** are unique, because they perforate calcareous rocks. For this reason they are the main eroders of coralligenous calcareous substrate. During the first stages of their life, they manifest in the form of papillae [Figure a) and b)] or little "chimney" [Figure c)]; then they grow up as mounds [Figure d)].

More photos: *C. celata*, *C. shmidtii*, *C. viridis*.

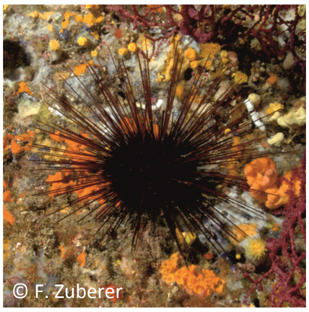

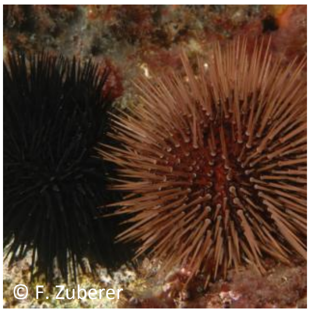

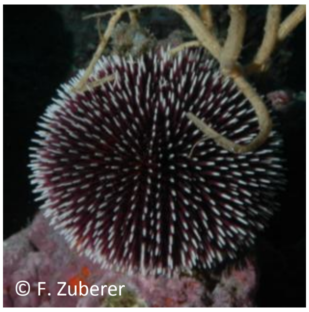

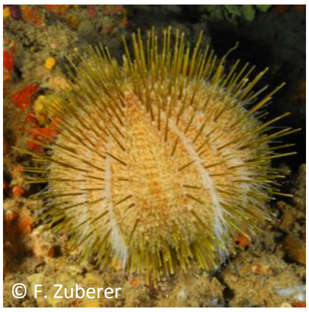

Centrostephanus longispinus Other sea urchins

Sea urchins graze coralligenous calcified red algae, thus contributing to the erosion of the calcareous substrate. ***Centrostephanus longispinus*** (the longspined sea urchin) is one of the most active grazers and is also a protected species. The **other sea urchins** (such as *Echinus melo*, *Gracilechinus acutus*, *Sphaerechinus granularis, Stylocidaris affinis, Paracentrotus lividus, Arbacia lixula*) are also contributing to coralligenous substrate erosion to a lower, however, degree.

More photos: *C. longispinus*, *E. melo*, *G. acutus*, *S. granularis*, *S. affinis*, *P. lividus*, *A. lixula*.

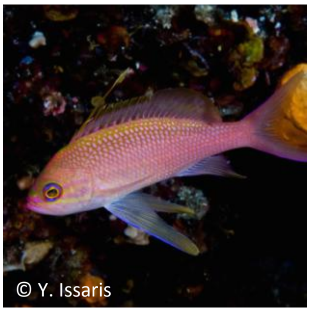

Anthias anthias

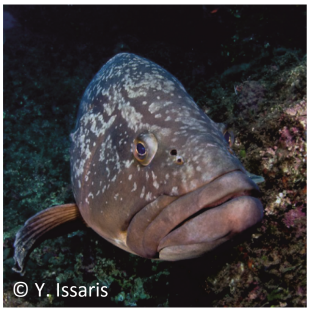

Epinephelus marginatus

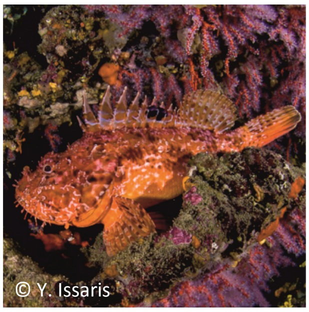

Scorpaena spp.

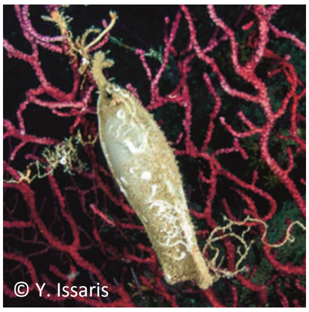

Shark eggs

Swallowtail sea-perches (***Anthias anthias***), dusky groupers (***Epinephelus marginatus***) and scorpionfishes (***Scorpaena* spp.**) are fishes that usually live in coralligenous habitats. Over the past decades, *E. marginatus* populations have experienced a dramatic reduction due to overfishing and are, therefore, protected by Law. The species of the genus *Scorpaena* are currently important elements of local fishery. **Sharks** and rays often deposit their **eggs** on gorgonians branches, using coralligenous habitat as a nursery ground.

More photos: *A. anthias*, *E. marginatus*, *Scorpaena maderensis*, *S. notata*, *S. porcus*, *S. scrofa*. Shark eggs.

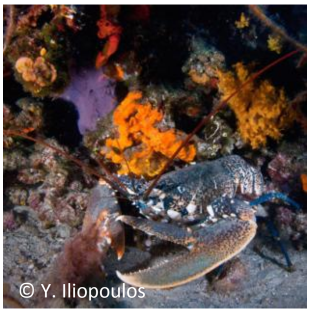

Homarus gammarus

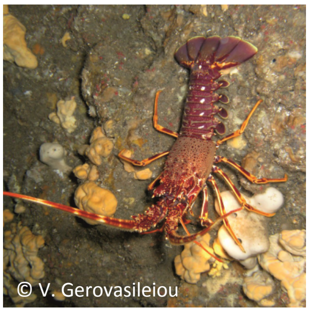

Palinurus elephas

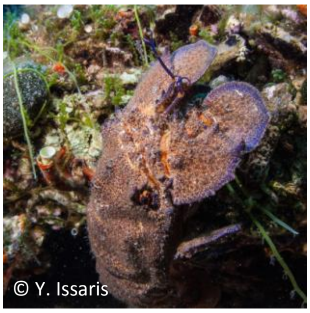

Scyllarides latus

These crustaceans are among the most commercially important inhabitants of coralligenous habitats, especially for their culinary value. For this reason, European lobsters (***Homarus gammarus***), spiny lobsters (***Palinurus elephas***) and locust lobsters (***Scyllarides latus***) harvesting is regulated.

More photos: *H. gammarus*, *P. elephas*, *S. latus*.

Its diving time now, what do I have to do?

An underwater tablet is provided to mark your observations, with a simple pencil. A filling order should be respected: from the top to the bottom and from left to right of the tablet. This is because the tablet is designed following some practical expedients that optimize the observation. For instance, the order of the species in the tablet is based on their size and therefore to the easiness that can be spotted: big and well visible species go first, gradually followed by smaller species that require a closer look to the substrate, or even species that are living in crevices. On the other hand, water temperature at the depth of the observation is located at the end of the tablet in order to give time to the diving computer to provide an accurate measurement.

No field is mandatory, but is strongly recommended not to forget to mark the depth of the observation.

ESSENTIAL EQUIPMENT: TABLET, SNAP-HOOK, TORCH, DIVING COMPUTER, COMPASS

Optional equipment: GPS, Underwater camera

Step by step application of the Protocol:

1. Make sure that pencils are operative (a backup pencil may also come in handy).
2. Mark date and name of the diving site (provide GPS coordinates if possible).
3. During the descent, mark the depth at which you met colder water, if you noticed it.
4. Once you reach the depth of your choice, choose the area of your observation: it could be a limited surface (not smaller than the width of your opened arms in length and width) or a small itinerary at constant depth. You can feel free to do whatever you want!
5. Mark: observation depth, current, visibility, observed depth range, horizontal extent, habitat continuity, slope, rugosity and orientation.
6. Observe if there are some invasive species or other pressures, and mark the class of abundance for each one.
7. Observe the species and assess their class of abundance.
8. Mark water temperature at the depth of the observation.
9. Back home: upload your data and eventual photographs in the website (http://cs.cigesmed.eu).
   Whether your diving computer provides for temperature data along the whole dive, you can also fill in the water temperature at predefined depths (every 10 m).
   When data uploading is completed successfully, please take a photo of the tablet and keep it as a back up. Then, you can erase the tablet with a simple rubber and use it again as many times as you want!

cs.cigesmed.eu

Name

Site

Date

At what depth you met colder water? m / never

Observation depth:

Current

None ☐ Weak ☐ Strong ☐

Visibility

Clear water ☐ Some particles ☐ Turbid ☐

Habitat extent

Verticale observed

Min depth:

Max depth:

Horizontal

<5 m ☐ 5-10 m ☐

10-20 m ☐ >20 m ☐

Habitat continuity

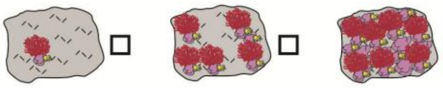

☐ ☐ ☐

Slope

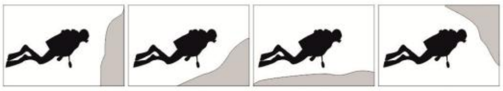

☐ ☐ ☐ ☐

Rugosity

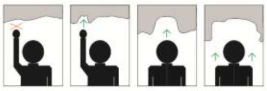

☐ ☐ ☐ ☐

Orientation

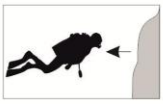

N ☐ S ☐

NE ☐ SW ☐

E ☐ W ☐

SE ☐ NW ☐

Pressures

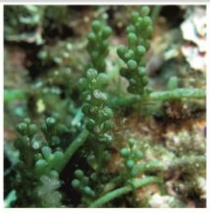

Caulerpa cylindracea

0 ☐ + ☐ ++ ☐

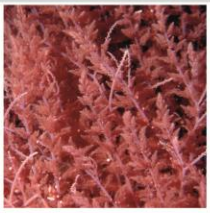

Asparagopsis spp.

0 ☐ + ☐ ++ ☐

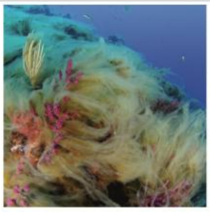

Mucilaginous aggregates

0 ☐ + ☐ ++ ☐

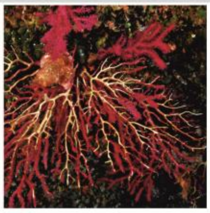

Necrosis/ mortality events

0 ☐ + ☐ ++ ☐

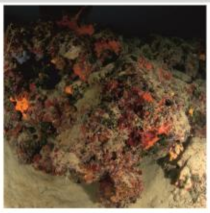

Sedimentation

0 ☐ + ☐ ++ ☐

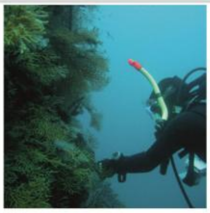

Divers recklessness marks

0 ☐ + ☐ ++ ☐

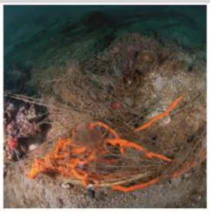

Fishing gears

0 ☐ + ☐ ++ ☐

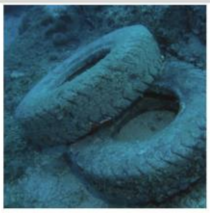

Litter

0 ☐ + ☐ ++ ☐

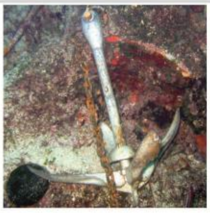

Anchoring

0 ☐ + ☐ ++ ☐

0 = absent

\+ = limited

++ = extended

Did you observe anything else?

Species

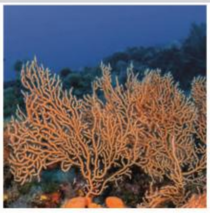

Eunicella cavolini

0 + ++ +++ ☐ ☐ ☐ ☐

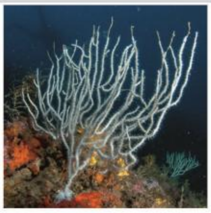

Eunicella singularis

0 + ++ +++ ☐ ☐ ☐ ☐

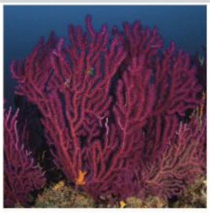

Paramuricea clavata

0 + ++ +++ ☐ ☐ ☐ ☐

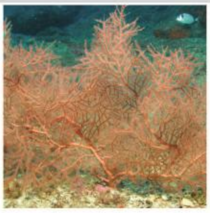

Leptogorgia sarmentosa

0 + ++ +++ ☐ ☐ ☐ ☐

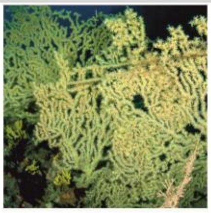

Savalia savaglia

0 + ++ +++ ☐ ☐ ☐ ☐

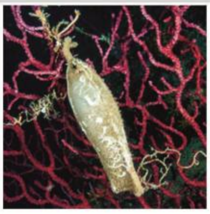

Shark eggs

0 + ++ +++ ☐ ☐ ☐ ☐

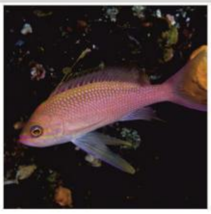

Anthias anthias

0 + ++ +++ ☐ ☐ ☐ ☐

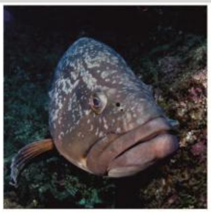

Epinephelus marginatus

0 + ++ +++ ☐ ☐ ☐ ☐

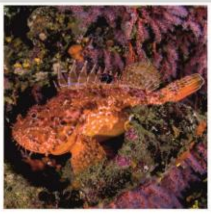

Scorpaena spp.

0 + ++ +++ ☐ ☐ ☐ ☐

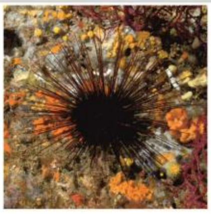

Centrostephanus longispinus

0 + ++ +++ ☐ ☐ ☐ ☐

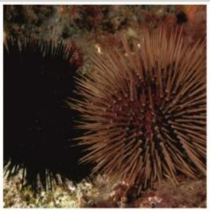

Other sea urchins

0 + ++ +++ ☐ ☐ ☐ ☐

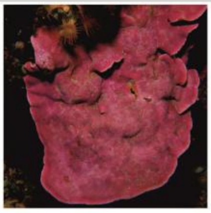

Calcareous red algae

0 + ++ +++ ☐ ☐ ☐ ☐

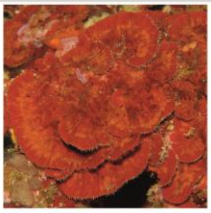

Peyssonnelia spp.

0 + ++ +++ ☐ ☐ ☐ ☐

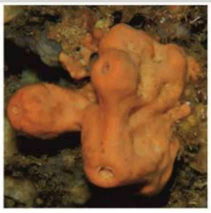

Agelas oroides

0 + ++ +++ ☐ ☐ ☐ ☐

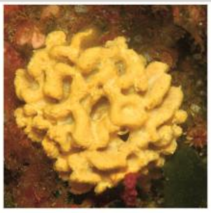

Axinella spp.

0 + ++ +++ ☐ ☐ ☐ ☐

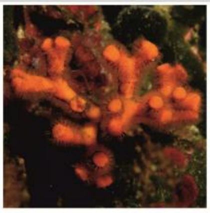

Myriapora truncata

0 + ++ +++ ☐ ☐ ☐ ☐

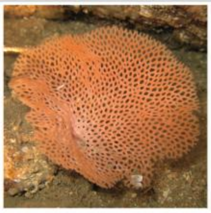

Other bryozoans

0 + ++ +++ ☐ ☐ ☐ ☐

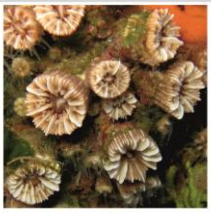

Scleractinians

0 + ++ +++ ☐ ☐ ☐ ☐

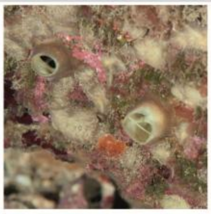

Cliona spp.

0 + ++ +++ ☐ ☐ ☐ ☐

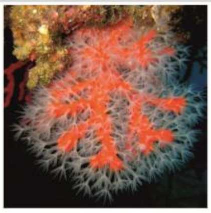

Corallium rubrum

0 + ++ +++ ☐ ☐ ☐ ☐

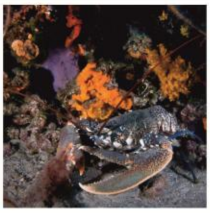

Homarus gammarus

0 + ++ +++ ☐ ☐ ☐ ☐

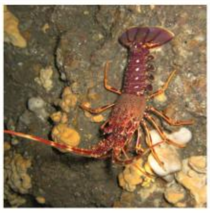

Palinurus elephas

0 + ++ +++ ☐ ☐ ☐ ☐

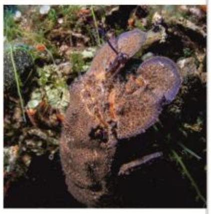

Scyllarides latus

0 + ++ +++ ☐ ☐ ☐ ☐

Water temperature at observation depth:

Citizen Science 4 CIGESMED

0 = absent + = scarce ++ = abundant +++ = very abundant

Guidelines to be cited as: Gatti G., Thierry de Ville d'Avray L., David R., Dimitriadis C., Gerovasileiou V., Dailianis T., Sini M., Salomidi M., Dogan A., Issaris Y., Çinar M.E., Koutsoubas D., Arvanitidis C., Feral J-P. 2015. CIGESMED for divers - Citizen Science for CIGESMED. SeasEra project (E.U. FP7 ERA-NET). 12 p.
