# Supplementary material for: CIGESMED for divers: Establishing a citizen science initiative for the mapping and monitoring of coralligenous assemblages in the Mediterranean Sea
Source: Biodivers Data J. 2016 Nov 1;(4):e8692. doi: 10.3897/BDJ.4.e8692 (PMC5136673; doi:10.3897/BDJ.4.e8692)
Supplement: Supplementary material 2 — CIGESMED pour les plongeurs – Les sciences participatives pour CIGESMED [file biodiversity_data_journal-4-e8692-s002.pdf]

http://cs.cigesmed.eu/fr www.cigesmed.eu

CIGESMED pour les plongeurs – Les Sciences Participatives pour CIGESMED

Le «coralligène», qu'est-ce que c'est ?

Le coralligène est un paysage sous-marin qui se développe dans des conditions de luminosité réduite et que l'on peut observer uniquement dans la mer Méditerranée. Créé par des algues rouges calcifiées qui s'accumulent depuis des millénaires sur les fonds marins rocheux, les récifs coralligènes sont comparables aux récifs coralliens tropicaux. A l'action constructrice des algues est opposée celle des erodeurs, avec comme exemple les éponges perforantes du genre *Cliona* et les oursins qui creusent le substrat calcaire et contribuent à augmenter sa complexité. Ces substrats calcaires soutiennent de nombreuses espèces (éponges, gorgones, bryozoaires, coraux) à longue durée de vie qui contribuent à la structuration des habitats coralligènes. Enfin, un grand nombre d'invertébrés (comme les nudibranches, les crustacés, les ascidies, les échinodermes, les mollusques) et des poissons trouvent leur abri idéal dans ces habitats coralligènes et participent ainsi à en faire de véritables trésors de biodiversité.

Pourquoi observer le coralligène ?

Parce que c'est un habitat unique au monde et aussi l'un des plus riches et spectaculaires qu'un plongeur peut rencontrer sous l'eau. Sa complexité abrite de nombreuses espèces d'une grande valeur écologique, esthétique et commerciale, dont certaines sont protégées. Une telle richesse de biodiversité est aujourd'hui menacée par tous les excès et les mauvaises pratiques de l'espèce humaine : mouillage sauvage, plongée irresponsable, (sur)pêche, abandon et déchargement d'ordures urbaines, etc. Autant de facteurs qui s'additionnent aux effets du réchauffement des eaux et à l'arrivée d'espèces invasives.

En participant au projet *CIGESMED for divers – Citizen Science for CIGESMED*, vous contribuez à l'exploration et la conservation des habitats coralligènes et du milieu marin, tout en ayant l'occasion d'améliorer vos connaissances sur la biodiversité marine.

Qu’est-ce qu’on observe ?

1. <u>Quelques informations générales sur le site</u>

**Température de l’eau à la profondeur de l’observation :**

La **température de l’eau** est un paramètre essentiel pour la survie des espèces, donc c’est important de la connaitre **à la profondeur de** votre **observation**.

**A partir de quelle profondeur avez-vous rencontré l ’eau la plus froide ?**	**m / jamais**

Cette profondeur correspond à la limite inférieure de la thermocline, la zone de transition thermique entre les eaux superficielles (plus chaudes) et les eaux plus profondes (plus froides). En période de canicule cette limite peut descendre plus en profondeur, donc les organismes entrent en contact avec de l’eau plus chaude que celle à laquelle ils sont habitués en temps normal. Si cette situation persiste, cela peut conduire à des épisodes de mortalité partielle ou massive de nombreuses espèces, notamment des gorgones et des éponges.

Dans la case dédiée, indiquez la température de l’eau à la profondeur de l’observation et la limite inférieure de la thermocline (**A partir de quelle profondeur avez-vous rencontré l’eau la plus froide ?**) en mètres, ou cochez l’option « jamais » si vous n’avez pas rencontré la thermocline.

Profondeur de l ’observation :	Courant	Visibilité
	Aucun☐ Faible☐ Fort☐	Eau Claire☐ Quelques particules☐ Trouble☐

Associer une **profondeur** à l’observation est primordial car c’est un facteur majeur quant à la composition et la distribution des habitats coralligènes. Notez la profondeur à laquelle vous avez effectué votre observation.

A une même profondeur, des informations par rapport à l’intensité du courant et à la visibilité complètent le cadre général des caractéristiques du site d’observation.

Pour le **courant**, il y a le choix entre trois catégories : **aucun**, **faible** ou **fort**.

Concernant la **visibilité** le choix se fait aussi entre trois classes : **eau claire** (vous avez une visibilité des plusieurs mètres), eau **trouble** (visibilité inferieure à 1-2 m) ou une catégorie intermédiaire appelée « **quelques particules** » en suspension.

Cochez la case correspondante à l’intensité de courant et à la visibilité observées.

2. <u>La configuration du site et de l’habitat</u>

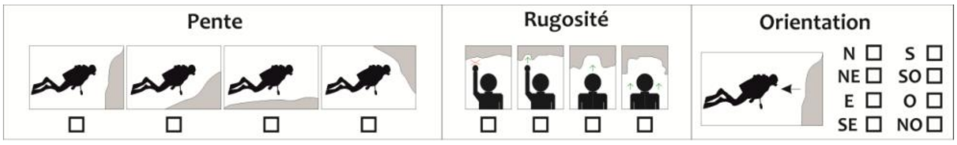

La pente, la rugosité et l’orientation du substrat sont, avec la profondeur, des facteurs qui ont une influence certaine sur la composition des habitats coralligènes.

Selon la **pente** du substrat, par exemple, les organismes peuvent être plus ou moins exposés à la lumière et à la sédimentation. Nous pouvons considérer quatre catégories de pente: « **verticale** », « **inclinée** », « **horizontale** » et « **surplomb** ».

La **rugosité** correspond à l'irrégularité de la roche : plus le substrat est irrégulier, plus grande sera la complexité de l'habitat. Une mesure simple de rugosité est basée sur des repères corporels : les anfractuosités peuvent être **trop petites pour pouvoir faire rentrer un poing**, ou **assez grandes pour faire passer un poing**, la **tête** ou même les **épaules**. Dans la majeure partie des cas, il n'y a pas qu'une seule taille des trous : il faut alors choisir la plus large, car il est très probable qu'elle contienne des anfractuosités plus petites.

L'**orientation** correspond à la direction vers laquelle le substrat « regarde ». Pour mesurer l'orientation il faut se mettre face à la paroi avec une boussole et relever la direction indiquée par la flèche imaginaire qui sort, perpendiculairement, de la paroi. Il est évident qu'un substrat horizontal ne peut pas avoir une orientation.

Cochez la case correspondante à la pente, à la taille des plus grands trous et à l'orientation observées.

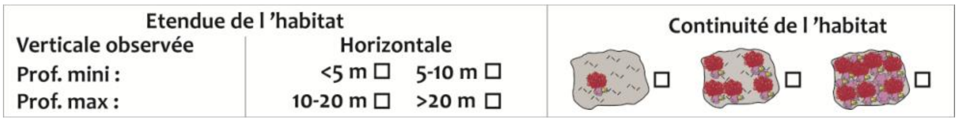

L'étendue verticale totale de l'habitat ne peut pas être observée en général, parce que la profondeur maximale dépasse souvent la profondeur accessible aux plongeurs. C'est pour cela qu'on parle d' « **étendue verticale <u>observée</u> de l'habitat** », qui se limite aux profondeurs maximale et minimale (en mètres) auxquelles on a observé l'habitat coralligène. Donc, vous pouvez estimer la profondeur atteinte par le coralligène en dessous de vous, ou vous pouvez simplement utiliser la profondeur maximale de votre plongée.

L' **étendue horizontale de l'habitat** fait référence à l'étendue horizontale de la paroi rocheuse couverte par le coralligène, dans le site d'observation. Il y a le choix entre des intervalles de distances : <5 m, 5-10 m, 10-20 m ou >20 m. Cochez la case correspondante à l'étendue horizontale de l'habitat observée.

La **continuité de l'habitat** indique si le coralligène se limite à une tache isolée entourée par une matrice (sédiment, roche nue) (voir dessin de gauche), s'il est discontinu (quelques endroits non recouverts, voir le dessin central) ou s'il est continu (sans interruptions remarquables) (voir dessin de droite). Cochez la case correspondante à la situation observée.

3. Les pressions qui agissent sur l'habitat

L'origine des pressions peut être naturelle ou humaine, mais le point commun est que toutes menacent la survie des espèces et la stabilité de l'habitat. Et même si dans certains cas, il est difficile d'intervenir pour améliorer la situation (comme pour les espèces invasives, par exemple), le suivi des pressions donne des informations essentielles pour pouvoir prendre des décisions et ainsi réduire certaines menaces (par ex. ancrage contrôlé, pêche limitée, plongée surveillée, etc.).

Pendant la plongée, vous pouvez **estimer l'impact des pressions** éventuelles. Pour chaque pression, présentée ci-dessous, vous pouvez donc choisir entre les niveau suivant : **0 = impact absent** ; **+ = impact limité** ; **++ = impact étendu**.

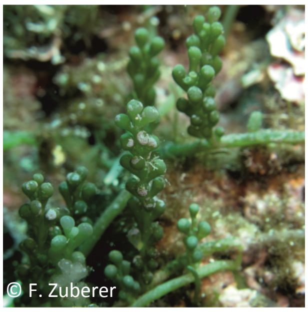

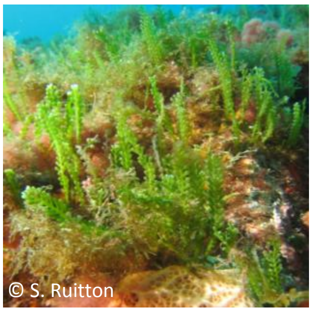

Caulerpa cylindracea

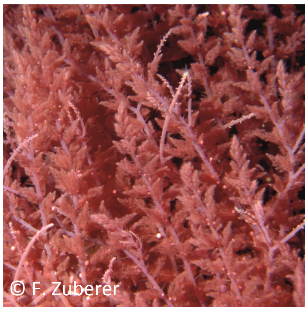

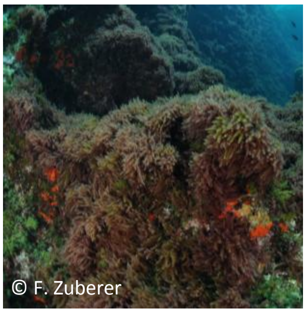

Asparagopsis spp.

L'algue verte *C. cylindracea* et les algues du genre *Asparagopsis* ont en commun deux caractéristiques. Premièrement elles sont **aliènes** en Méditerranée, c'est-à-dire qu'elles ne sont pas originaires de notre mer. Ces espèces sont tropicales, et elles ont trouvé un environnement idéal pour leur développement grâce au réchauffement des eaux marines superficielles. Deuxièmement elles sont **invasives**, c'est-à-dire qu'elles ont tendance à occuper l'espace disponible en étouffant tout ce qu'elles trouvent sur leur chemin. Etant une menace pour la biodiversité locale et pour la stabilité des habitats, il est important de connaitre la localisation et d'estimer l'abondance de ces espèces.

Plus de photos : *C. cylindracea*, *Asparagopsis* spp.

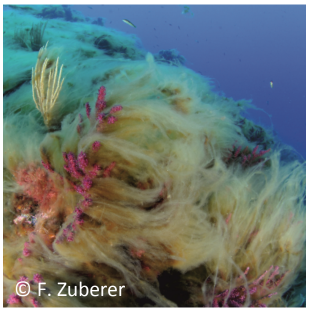

Présence de mucilage

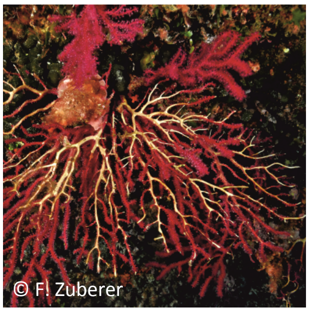

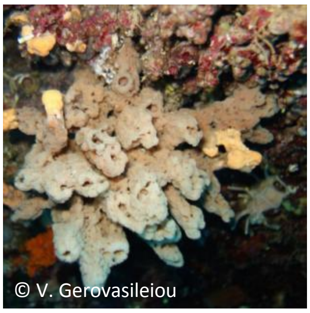

Nécrose / mortalité

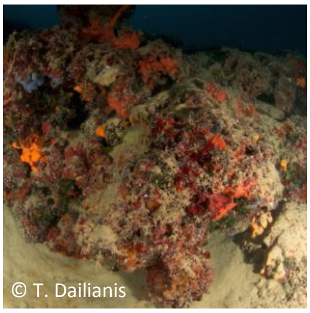

Sédimentation

Le **mucilage** est un agrégat des différentes algues microscopiques qui produisent des substances gélatineuses dont l'aspect est jaunâtre et filamenteux. Dans certaines conditions de température et de circulation de l'eau, le mucilage connaît une telle croissance qu'il recouvre les fonds marins et étouffe ainsi tous les organismes.

Le mucilage et des températures exceptionnellement hautes pendant une longue durée provoquent la **nécrose** (la mort des tissus) partielle ou totale des organismes et, dans le pire des cas, des **évènements de mortalité** massives des populations (l'ensemble des individus d'une même espèce).

Autre menace : la **sédimentation** intense. Elle peut être due à des évènements naturels, par ex. des pluies abondantes, ou à des activités humaines, soit ponctuelles (travaux côtiers) soit fixes (rejets d'égouts). Quelle que soit son origine, la sédimentation excessive sur les substrats rocheux entraîne l'ensevelissement des organismes et la dégradation de l'habitat.

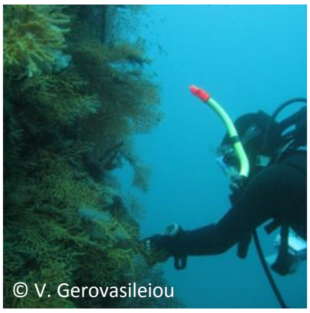

Dégâts imputables aux plongeurs

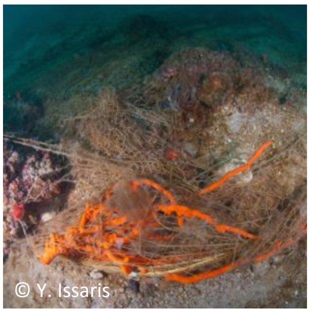

Engins de pêche

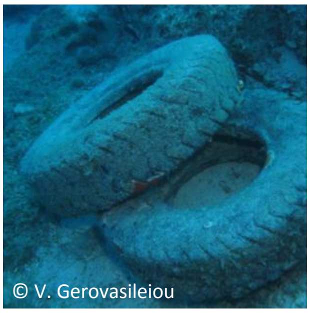

Déchets

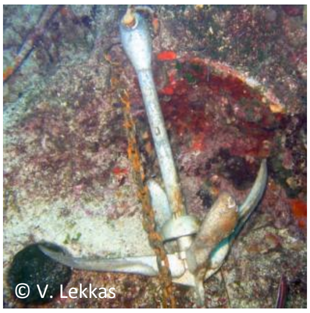

Marques d'ancrage/ancres

Les **déchets** de différentes natures, les **engins de pêche** abandonnés et l'**ancrage** peuvent provoquer localement des dommages considérables sur les habitats coralligènes.

Le comportement irresponsable de la part de **plongeurs** entraine les mêmes conséquences. Les plongeurs inattentifs qui touchent les organismes avec leur corps ou leur matériel, risquent de les endommager. Entre autres, les bryozoaires, organismes calcifiés érigés, sont particulièrement sensibles aux dommages mécaniques, et ils sont souvent utilisés comme indicateurs de fréquentation en plongée. Si en regardant au pied de la falaise on aperçoit de nombreux morceaux ou des colonies entières de bryozoaires et des gorgonaires, on peut en déduire que le site est fréquenté par des plongeurs irresponsables.

4. Les espèces

Dans la liste ci-dessous, nous avons sélectionné un nombre limité d'espèces qui sont importantes de par le rôle qu'elles jouent dans l'habitat (par exemple constructeurs, erodeurs, structurant du paysage), et/ou parce qu'elles peuvent être indicatrices d'un impact ou d'une situation particulière, ou encore parce qu'elles sont protégées (lois nationales et internationales) ou simplement typiques des habitats coralligènes. Les informations relatives à leur présence et à leur abondance approximative permettent de décrire l'habitat et d'en estimer l'état de santé.

Pour l'**estimation de l'abondance**, pour chaque espèce (ou groupe d'espèces) vous pouvez choisir entre les classes suivantes : **0 = absente ; + = peu abondante ; ++ = abondante ; +++ = très abondant**. Ayez confiance en votre jugement!

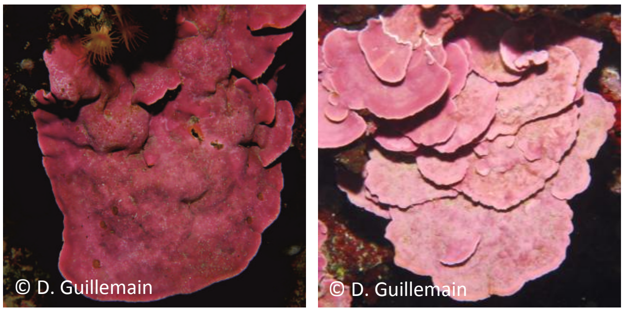

Algues rouges calcaires

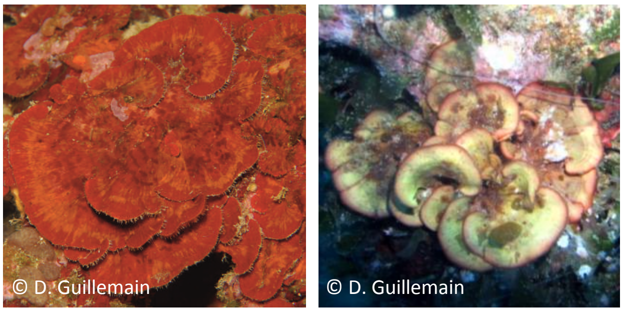

Peyssonnelia spp.

Ces algues rouges sont les organismes végétaux les plus représentés dans les habitats coralligènes.
Les **algues rouges calcaires**, notamment *Lithophyllum stictaeforme/cabiochiae* et *Mesophyllum expansum*, sont les principaux constructeurs du substrat calcaire. Les algues du genre ***Peyssonnelia*** peuvent compter des espèces calcifiées (*Peyssonnelia rosa-marina*) ou non calcifiées (*Peyssonnelia squamaria*).

Plus de photos : *L. stictaeforme/cabiochiae*, *M. expansum*, *P. rosa-marina*, *P. squamaria*.

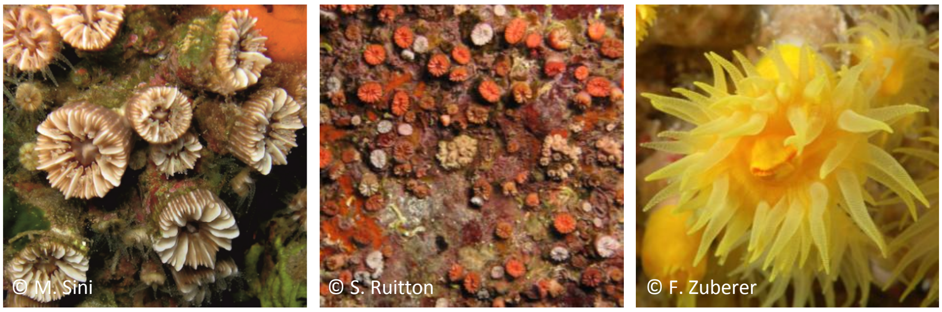

Scléractiniaires

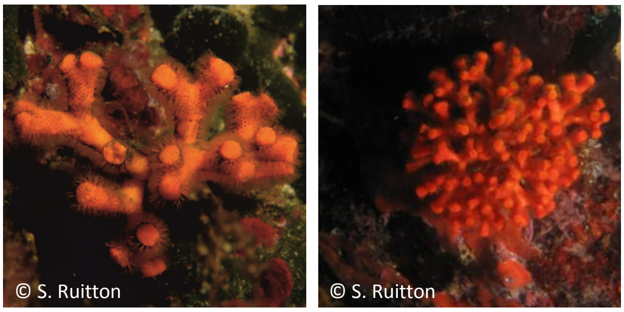

Myriapora truncata

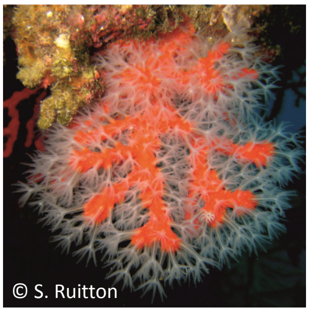

Corallium rubrum

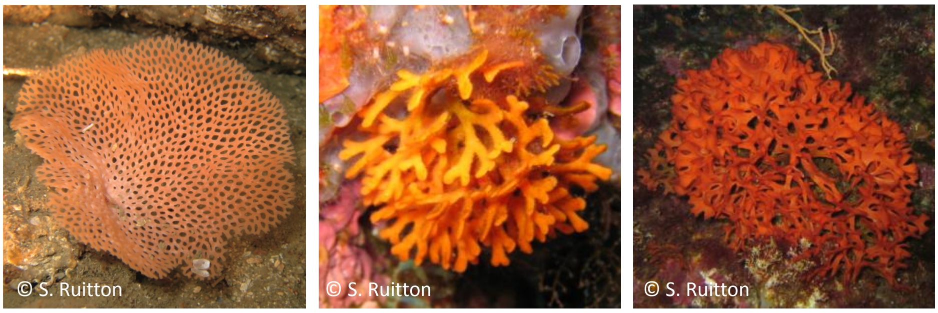

Autres bryozoaires

Les **scléractiniaires** et les bryozoaires (***Myriapora truncata*** et **autres**), avec leur squelette calcaire, sont les constructeurs secondaires du substrat calcaire du coralligène. Les bryozoaires arbustifs sont fragiles et peuvent facilement être cassés lors d'un impact mécanique : pour cette raison, ils sont utilisés comme indicateurs d'impact des plongeurs avec une attitude inattentive. Les bryozoaires autres que *M. truncata*, sont aussi considérés indicateurs de pollution de l'eau, vue leur sensibilité à cette pression. Attention! Ne pas confondre *M. truncata*, qu'on appelle communément le "faux corail", avec le corail rouge, ***Corallium rubrum***. En effet, le corail rouge, malgré son nom, n'est pas vraiment un corail, mais c'est le seul gorgonaire qui produise un squelette calcaire. Ainsi, il est considéré parmi les constructeurs secondaires du substrat coralligène. Récemment, *C. rubrum* a été classé parmi les espèces « en danger » dans la Liste Rouge de l'UICN.

Plus de photos : Scléractiniaires - *Caryophyllia (Caryophyllia) smithii*, *Caryophyllia inornata*, *Hoplangia durotrix*, *Leptopsammia pruvoti*, *Madracis pharensis*, *Phyllangia mouchezii*, *Polycyathus muellerae*. *M. truncata*. *C. rubrum*. Autres bryozoaires - *Adeonella calveti*, *Pentapora fascialis*, *Smittina cervicornis*.

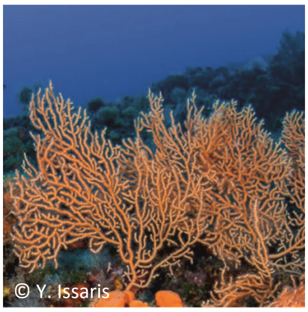

Eunicella cavolini

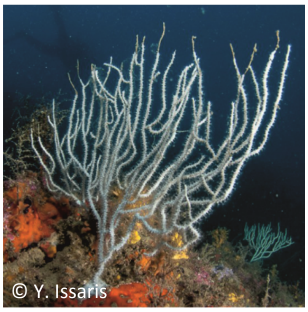

Eunicella singularis

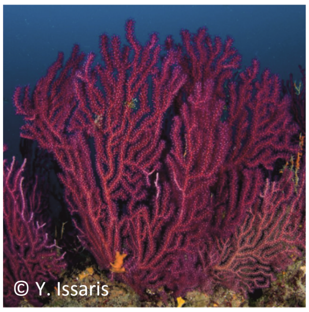

Paramuricea clavata

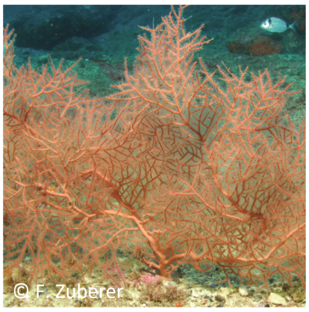

Leptogorgia sarmentosa

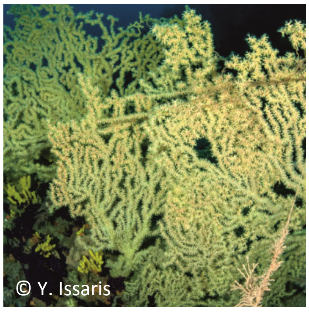

Savalia savaglia

Entre les éléments structurant du paysage coralligène, ces espèces arborescentes sont surement les plus remarquables. Les gorgones jaunes (***Eunicella cavolini***), blanches (***Eunicella singularis***) et rouges (***Paramuricea clavata***) sont les plus communes. ***Leptogorgia sarmentosa***, la gorgone orange, préfère les eaux plus troubles et elle est donc considérée comme indicatrice de turbidité. ***Savalia savaglia***, le faux corail noir, n'est pas une gorgone, même s'il en imite l'aspect, mais est plus proche des anémones et coraux ; en étant une espèce assez rare (et profonde), toute signalisation de sa présence est importante pour estimer sa distribution, qui est mal connue dans certaines zones de la Méditerranée. *P. clavata* a été récemment classée espèce « vulnérable », et *E. cavolini*, *E. singularis* et *S. savaglia* ont été classées parmi les espèces « quasi menacées » dans la Liste Rouge de l'UICN.

Plus de photos : *E. cavolini*, *E. singularis*, *P. clavata*, *L. sarmentosa*, *S. savaglia*.

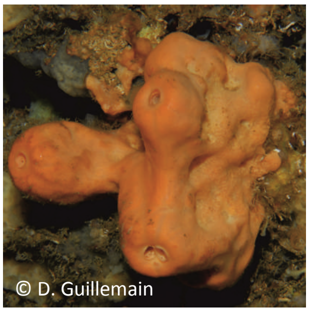

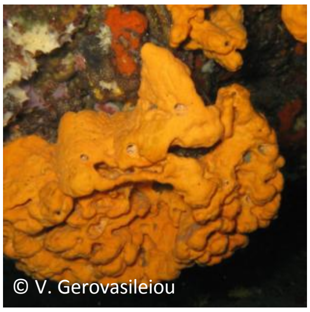

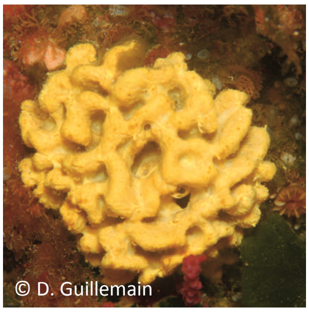

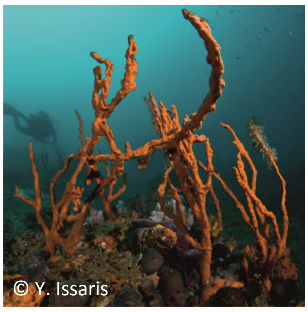

Agelas oroides *Axinella* spp.

Le nombre d'espèces d'éponges observables dans les habitats coralligènes est difficile à calculer. Nous avons donc choisi les espèces les plus répandues pour représenter cette catégorie : ***Agelas oroides*** et les espèces du genre ***Axinella*** (*Axinella damicornis, A. verrucosa, A. polypoides* (protégé)).

Plus de photos : *A. oroides*, *A. damicornis*, *A. verrucosa*, *A. polypoides*.

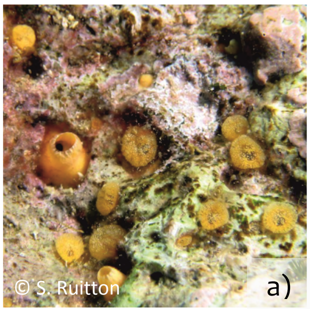

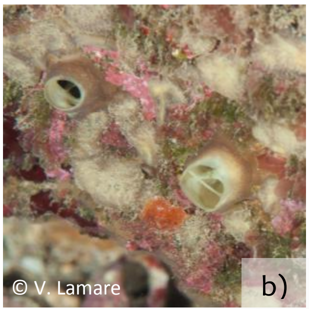

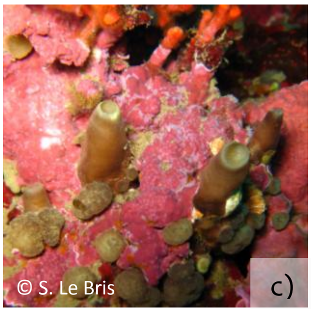

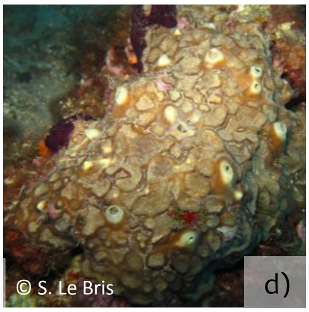

Cliona spp.

Les éponges du genre ***Cliona*** ont la particularité d'être perforantes : elles creusent la roche calcaire et pour cela, elles sont les principales erodeurs du substrat calcaire du coralligène. Dans les premières étapes de leur vie, elles apparaissent sous forme de papilles [photos a) et b)] ou petits « chemins » [photo c)] pour ensuite arriver à une forme massive dans une étape plus avancée [photo d)].

Plus de photos : *C. celata*, *C. shmidtii*, *C. viridis*.

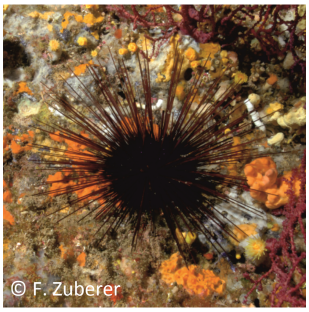

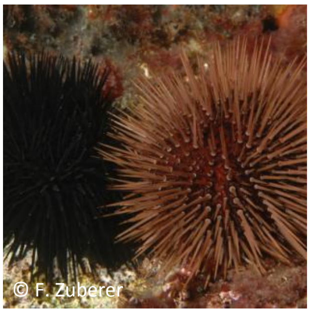

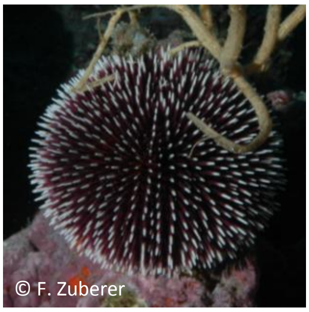

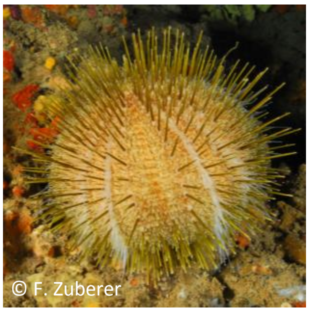

Centrostephanus longispinus Autres oursins

Les oursins broutent les algues rouges calcifiées et contribuent activement à l'érosion du substrat calcaire. ***Centrostephanus longispinus*** (l'oursin diadème), l'un des plus actifs, est aussi une espèce protégée. Les **autres oursins** (notamment *Echinus melo*, *Gracilechinus acutus*, *Sphaerechinus granularis*, *Stylocidaris affinis*, *Paracentrotus lividus*, *Arbacia lixula*) participent dans une moindre mesure à l'érosion du substrat du coralligène.

Plus de photos : *C. longispinus*, *E. melo*, *G. acutus*, *S. granularis*, *S. affinis*, *P. lividus*, *A. lixula*.

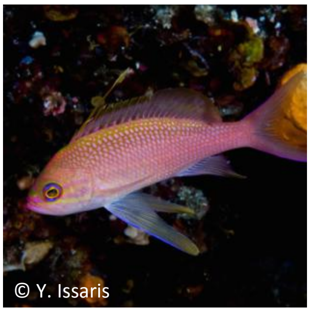

Anthias anthias

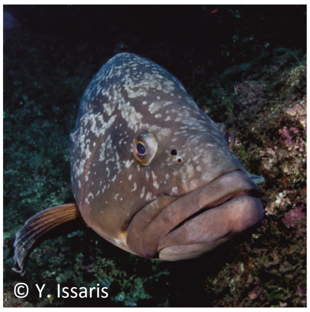

Epinephelus marginatus

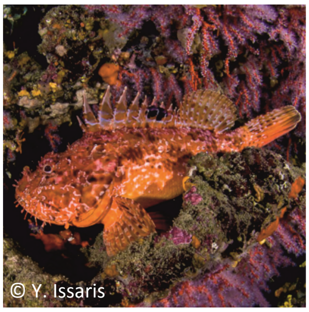

Scorpaena spp.

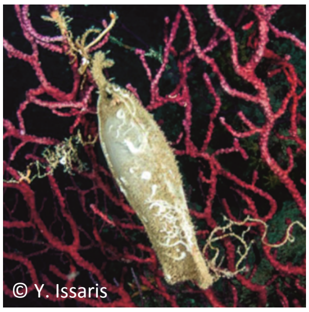

Œufs de sélaciens

Barbiers (***Anthias anthias***), mérous (***Epinephelus marginatus***), rascasses et chapons (quatre espèces différentes qui appartiennent au genre ***Scorpaena***) sont des poissons qui choisissent leur abri habituel dans les habitats coralligènes. *E. marginatus* est une espèce qui a vécu une dramatique réduction à cause de la surpêche mais qui est maintenant protégée. Les espèces du genre *Scorpaena* sont une composante importante de la petite pêche locale actuelle. Requins et raies déposent leurs œufs sur les branches des gorgonaires, donc ils utilisent l'habitat coralligène come une nurserie.

Plus de photos: *A. anthias*, *E. marginatus*, *Scorpaena maderensis*, *S. notata*, *S. porcus*, *S. scrofa*. Œufs de sélaciens.

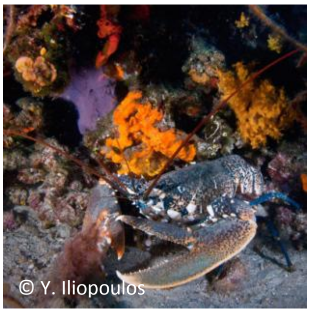

Homarus gammarus

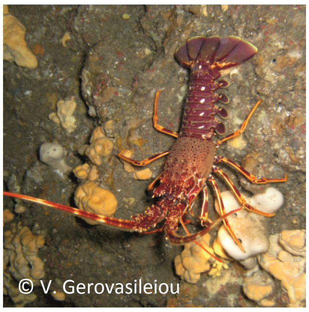

Palinurus elephas

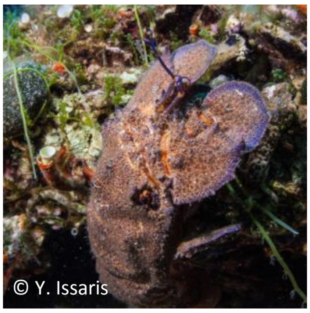

Scyllarides latus

Ces crustacés font partie des habitants les plus recherchés des habitats coralligènes, typiquement pour leur valeur culinaire. Pour cette raison, la récolte de homards (***Homarus gammarus***), langoustes (***Palinurus elephas***) et cigales (***Scyllarides latus***) est réglementé. La cigale est aussi totalement protégée en France.

Plus de photos : *H. gammarus*, *P. elephas*, *S. latus*.

Enfin, comment s'y prendre pour appliquer le protocole ?

Le protocole d’observation prévoit l’utilisation d’une tablette (voir après) sur laquelle vous pouvez inscrire vos observations avec un crayon à papier. La tablette doit être remplie dans l’ordre : de gauche à droite et de haut vers le bas. Cette configuration permet d’éviter les oublis et la classification se fait selon la visibilité des espèces : les premières correspondent aux plus visibles en arrivant sur le site d’observation, puis au fur et à mesure qu’on se rapproche de la paroi, on peut remarquer les espèces les plus petites ou cachées dans les trous, qui nécessitent d’être plus près de la paroi. De façon similaire, la température de l’eau est demandée à la fin de l’observation, pour laisser à l’ordinateur subaquatique le temps nécessaire à relever la température réelle.

Aucun champs n’est obligatoire, bien entendu, mais il est fortement recommandé de ne pas oublier de relever la profondeur de l’observation.

Outils nécessaires : tablette, lampe, ordinateur de plongée, boussole
Outils optionnels : GPS, appareil photographique

Schéma d’application du protocole :

1. Assurez-vous que les crayons soient opérationnels (un crayon en plus peut toujours être outil).
2. Relevez la date et le nom du site de plongée (fournissez les coordonnées GPS si possible)
3. Pendant la descente, pensez à relever la profondeur à laquelle l’eau devient plus froide, si vous la remarquez.
4. Une fois arrivé à la profondeur souhaitée, choisissez la surface d’observation : vous pouvez choisir d’observer une surface limitée (de la largeur minimale de vos deux bras ouverts) ou le long d’un petit parcours en maintenant toujours la même profondeur. Vous pouvez faire ce que vous voulez !
5. Inscrivez : profondeur de l’observation, courant, visibilité, étendue verticale observée, étendue horizontale, continuité de l’habitat, pente, rugosité et orientation.
6. Contrôlez s’il y a des espèces invasives et d’autres pressions, estimez et marquez le niveau de chaque pression.
7. Observez les espèces présentes et relevez leur abondance.
8. Relevez la température de l’eau à la profondeur de l’observation.
9. De retour chez vous : saisissez vos données (et éventuellement vos photos) sur le site internet (http://cs.cigesmed.eu). Si votre ordinateur de plongée enregistre les données relatives à la température de l’eau tout au long de la plongée, vous pouvez renseigner les valeurs de la température tous le 10 m de profondeur.
   Lorsque vous avez terminé, prenez une photo de la plaquette et conservez-la. Cela pourra éventuellement être utile un jour ! Enfin, vous pouvez effacer la plaquette avec une gomme et la réutiliser autant de fois que vous le souhaitez !

BRAVO et MERCI pour votre contribution !

cs.cigesmed.eu/fr

Nom

Site

Date

A partir de quelle profondeur avez-vous rencontré l 'eau la plus froide ? m / jamais

Profondeur de l 'observation :	Courant	Visibilité
	Aucun ☐ Faible ☐ Fort ☐	Eau Claire ☐ Quelques particules ☐ Trouble ☐

Etendue de l 'habitat

Verticale observée

Prof. mini :

Prof. max :

Horizontale

<5 m ☐ 5-10 m ☐

10-20 m ☐ >20 m ☐

Continuité de l 'habitat

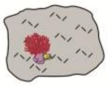 ☐ 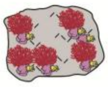 ☐ 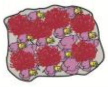 ☐

Pente

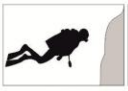 ☐ 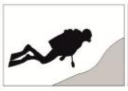 ☐ 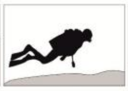 ☐ 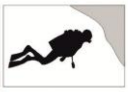 ☐

Rugosité

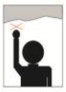 ☐ 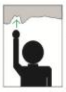 ☐ 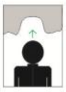 ☐ 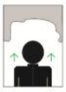 ☐

Orientation

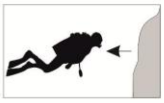

N ☐ S ☐

NE ☐ SO ☐

E ☐ O ☐

SE ☐ NO ☐

Pressions

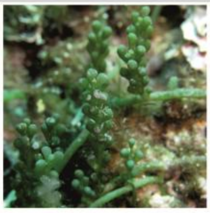

Caulerpa cylindracea

0 ☐ + ☐ ++ ☐

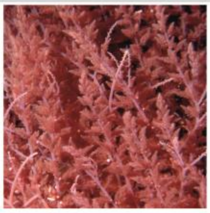

Asparagopsis spp.

0 ☐ + ☐ ++ ☐

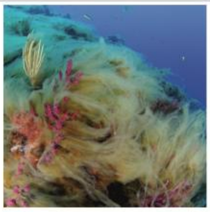

Présence de mucilage

0 ☐ + ☐ ++ ☐

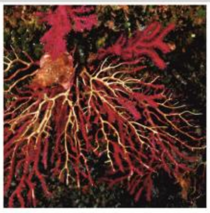

Nécrose / mortalité

0 ☐ + ☐ ++ ☐

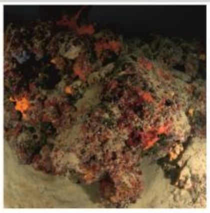

Sédimentation

0 ☐ + ☐ ++ ☐

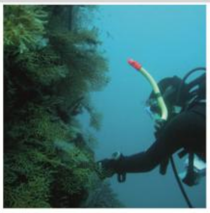

Dégâts imputables aux plongeurs

0 ☐ + ☐ ++ ☐

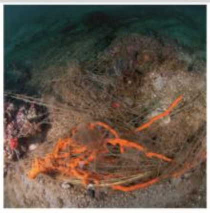

Engins de pêche

0 ☐ + ☐ ++ ☐

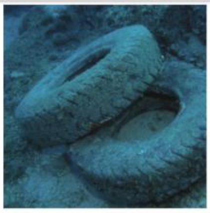

Déchets

0 ☐ + ☐ ++ ☐

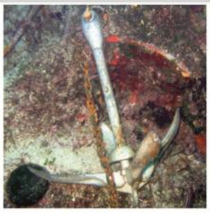

Marques d'ancrage/ ancres

0 ☐ + ☐ ++ ☐

0 = absent

\+ = limité

++ = étendu

Avez-vous observé autre chose ?

Espèces

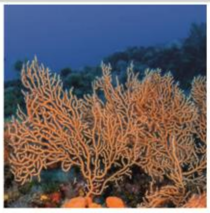
Eunicella cavolini
0 ☐ + ☐ ++ ☐ +++ ☐

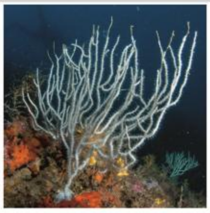
Eunicella singularis
0 ☐ + ☐ ++ ☐ +++ ☐

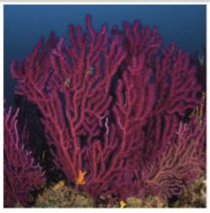
Paramuricea clavata
0 ☐ + ☐ ++ ☐ +++ ☐

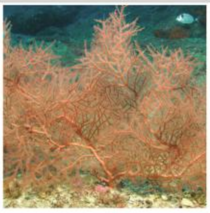
Leptogorgia sarmentosa
0 ☐ + ☐ ++ ☐ +++ ☐

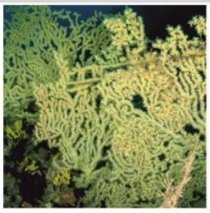
Savalia savaglia
0 ☐ + ☐ ++ ☐ +++ ☐

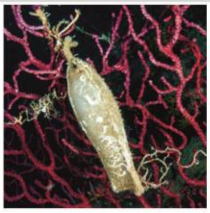
Œufs de sélaciens
0 ☐ + ☐ ++ ☐ +++ ☐

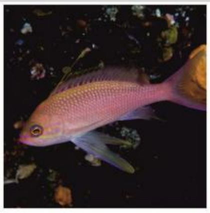
Anthias anthias
0 ☐ + ☐ ++ ☐ +++ ☐

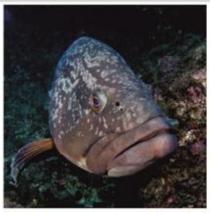
Epinephelus marginatus
0 ☐ + ☐ ++ ☐ +++ ☐

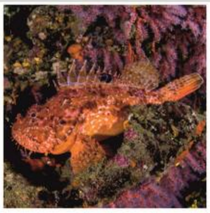
Scorpaena spp.
0 ☐ + ☐ ++ ☐ +++ ☐

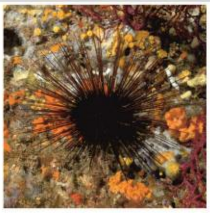
Centrostephanus longispinus
0 ☐ + ☐ ++ ☐ +++ ☐

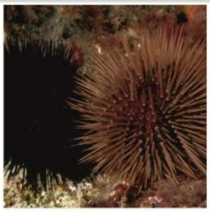
Autres oursins
0 ☐ + ☐ ++ ☐ +++ ☐

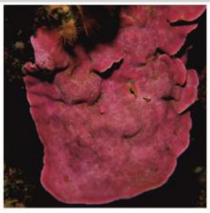
Algues rouges calcaires
0 ☐ + ☐ ++ ☐ +++ ☐

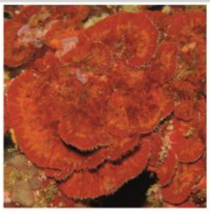
Peyssonnelia spp.
0 ☐ + ☐ ++ ☐ +++ ☐

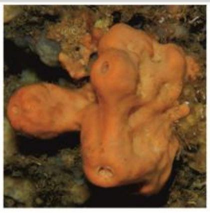
Agelas oroides
0 ☐ + ☐ ++ ☐ +++ ☐

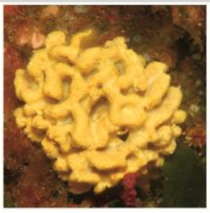
Axinella spp.
0 ☐ + ☐ ++ ☐ +++ ☐

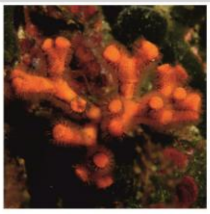
Myriapora truncata
0 ☐ + ☐ ++ ☐ +++ ☐

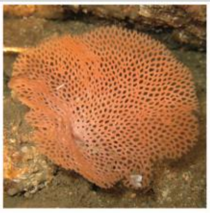
Autres bryozoaires
0 ☐ + ☐ ++ ☐ +++ ☐

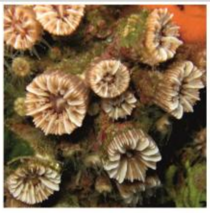
Scléractiniaires
0 ☐ + ☐ ++ ☐ +++ ☐

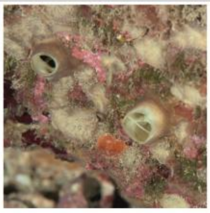
Cliona spp.
0 ☐ + ☐ ++ ☐ +++ ☐

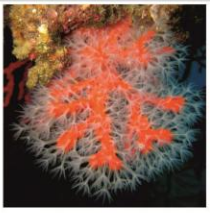
Corallium rubrum
0 ☐ + ☐ ++ ☐ +++ ☐

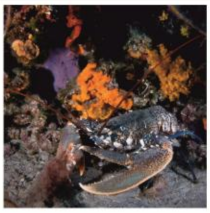
Homarus gammarus
0 ☐ + ☐ ++ ☐ +++ ☐

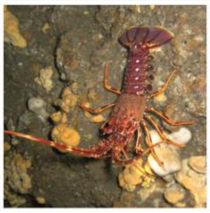
Palinurus elephas
0 ☐ + ☐ ++ ☐ +++ ☐

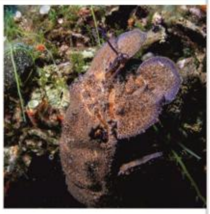
Scyllarides latus
0 ☐ + ☐ ++ ☐ +++ ☐

Température de l'eau à la profondeur de l'observation :

0 = absent + = peu abondant ++ = abondant +++ = très abondant

Pour citer ce document : Gatti G., Thierry de Ville d'Avray L., David R., Dimitriadis C., Gerovasileiou V., Dailianis T., Sini M., Salomidi M., Dogan A., Issaris Y., Çinar M.E., Koutsoubas D., Arvanitidis C., Feral J-P. 2015. CIGESMED pour les plongeurs – Les Sciences Participatives pour CIGESMED. SeasEra project (E.U. FP7 ERA-NET). 12 p.
