# Supplementary material for: CIGESMED for divers: Establishing a citizen science initiative for the mapping and monitoring of coralligenous assemblages in the Mediterranean Sea
Source: Biodivers Data J. 2016 Nov 1;(4):e8692. doi: 10.3897/BDJ.4.e8692 (PMC5136673; doi:10.3897/BDJ.4.e8692)
Supplement: Supplementary material 3 — CIGESMED για δύτες, πολίτες-επιστήμονες για το πρόγραμμα παρακολούθησης των κοραλλιγενών οικοτόπων [file biodiversity_data_journal-4-e8692-s003.pdf]

http://cs.cigesmed.eu www.cigesmed.eu

CIGESMED για Δύτες, Πολίτες-Επιστήμονες για το πρόγραμμα παρακολούθησης των κοραλλιγενών οικοτόπων

Τι είναι οι ‘κοραλλιγενείς’ οικότοποι;

Οι κοραλλιγενείς οικότοποι, αποτελούν ένα ιδιαίτερο υποβρύχιο τοπίο, που απαντά αποκλειστικά στη θάλασσα της Μεσογείου. Αναπτύσσεται πάνω σε σκιερούς βραχώδεις πυθμένες, κυρίως από ενασβεστωμένα ροδοφύκη των τάξεων Corallinales και Peyssonneliales, οι αλλεπάλληλες αποθέσεις των οποίων μπορούν σε βάθος χρόνου να σχηματίσουν πολυδιάστατες υποβρύχιες δομές και υφάλους. Πλήθος άλλων ειδών-βιοκατασκευαστών συμμετέχουν σε αυτή τη βιογενή διεργασία οικοδομώντας (π.χ. γοργονίες, σκληρακτίνια, σπόγγοι, βρυόζωα, πολύχαιτοι) ή αποδομώντας («βιοδιαβρωτές», π.χ. σπόγγοι του γένους *Cliona* που διατρυπούν, ή αχινοί που θρυμματίζουν το ασβεστώδες υπόστρωμα), αυξάνοντας διαρκώς τη δομική πολυπλοκότητα των κοραλλιγενών σχηματισμών. Ακριβώς λόγω της πολυπλοκότητας τους, οι δομές αυτές αποτελούν σημαντικό καταφύγιο για μεγάλο αριθμό ασπονδύλων (π.χ. καρκινοειδή, εχινόδερμα, μαλάκια, ασκίδια) και ψαριών, γεγονός που τις καθιστά πυρήνες θαλάσσιας βιοποικιλότητας. Κατά κανόνα, οι κοραλλιγενείς οικότοποι χαρακτηρίζονται από υψηλή αισθητική αξία αλλά και υψηλή ευπάθεια λόγω των εξαιρετικά αργών ρυθμών αύξησης των επιμέρους ειδών τους.

Γιατί μελετούμε τους κοραλλιγενείς οικοτόπους;

Οι θαλάσσιοι αυτοί οικότοποι είναι μοναδικοί σε παγκόσμιο επίπεδο και κατατάσσονται μεταξύ των ομορφότερων και πλουσιότερων σε ζωή θαλάσσιων τοπίων που μπορεί κανείς να προσεγγίσει με αυτόνομη κατάδυση. Λόγω της δομικής πολυπλοκότητας που παρουσιάζουν, φιλοξενούν πλήθος ειδών μεγάλης οικολογικής, αισθητικής και εμπορικής αξίας, ορισμένα από τα οποία προστατεύονται από την Εθνική αλλά και τη Διεθνή νομοθεσία. Οι κοραλλιγενείς οικότοποι συχνά απειλούνται από τις ανθρώπινες δραστηριότητες. Η ανεξέλεγκτη αγκυροβολία, η κατάδυση με έλλειψη περιβαλλοντικής ευαισθητοποίησης, η υπεραλίευση και τα απορρίμματα, σε συνδυασμό με την εισβολή αλλόχθονων ειδών και την αύξηση της θερμοκρασίας της θάλασσας (λόγω κλιματικής αλλαγής), αποτελούν τις κυριότερες απειλές που μπορεί να υποβαθμίσουν σημαντικά την κατάσταση των κοραλλιγενών οικοτόπων.

Με τη συμμετοχή σας στη δράση 'CIGESMED για δύτες, πολίτες-επιστήμονες' μπορείτε να συνεισφέρετε ενεργά στην εξερεύνηση και προστασία των κοραλλιγενών οικοτόπων και του θαλάσσιου περιβάλλοντος γενικότερα, εμπλουτίζοντας παράλληλα τη γνώση σας σχετικά με τη θαλάσσια βιοποικιλότητα.

Τι παρατηρούμε / καταγράφουμε;

1. Γενικές πληροφορίες για την τοποθεσία

**Θερμοκρασία νερού στο βάθος παρατήρησης:**

Δεδομένου ότι η θερμοκρασία αποτελεί σημαντική παράμετρο για την επιβίωση των θαλάσσιων ειδών, παρακαλούμε να σημειώσετε τη **θερμοκρασία του νερού στο βάθος της παρατήρησής σας.**

**Σε ποιο βάθος αισθανθήκατε σημαντική πτώση της θερμοκρασίας?**	**m / ποτέ**

Σημειώστε το βάθος στο οποίο αισθανθήκατε την πιο έντονη πτώση της θερμοκρασίας. Συνήθως πρόκειται για το κατώτερο όριο του θερμοκλινούς, στη μεταβατική ζώνη μεταξύ των ρηχών (θερμότερων) και βαθύτερων (ψυχρότερων) υδάτων. Μερικές φορές, το όριο αυτό μπορεί να βρίσκεται βαθύτερα από ότι συνήθως αναμένεται, εξαιτίας της παρουσίας θερμότερων ρευμάτων. Το γεγονός αυτό έχει ως αποτέλεσμα οι οργανισμοί που ζουν κανονικά σε ψυχρότερες συνθήκες να έρχονται σε επαφή με θερμά ύδατα. Αν το φαινόμενο αυτό έχει μεγάλη χρονική διάρκεια, μπορεί να προκαλέσει μερικό ή μαζικό θάνατο των πλέον ευαίσθητων ειδών και ιδιαίτερα των γοργονιών και των σπόγγων. Παρακαλούμε συμπληρώστε στο σχετικό πεδίο του πίνακα το βάθος (σε μέτρα) του ορίου εμφάνισης του θερμοκλινούς ή επιλέξτε την επιλογή 'ποτέ' αν δεν συναντήσατε θερμοκλινές.

**Βάθος παρατήρησης:**	**Ένταση του ρεύματος**	**Ορατότητα**
	**Απουσία ☐ Ήπιο☐ Δυνατό☐**	**Διαυγές νερό☐ Μερικά σωματίδια☐ Θολά ☐**

Το **βάθος** αποτελεί έναν σημαντικό παράγοντα που καθορίζει τη σύνθεση και κατανομή των κοραλλιγενών κοινοτήτων. Παρακαλούμε σημειώστε το βάθος (σε μέτρα) στο οποίο πραγματοποιείται η καταγραφή σας.

Οι πληροφορίες για την **ένταση του ρεύματος** και την **ορατότητα** στη στήλη του νερού (διαύγεια υδάτων) συμπληρώνουν τη γενική περιγραφή της τοποθεσίας όπου πραγματοποιείται η καταγραφή, άρα θα πρέπει να καταγράφονται στο ίδιο βάθος. Σχετικά με την **ένταση του ρεύματος** μπορείτε να επιλέξετε μια από τις παρακάτω κατηγορίες: **απουσία ρεύματος, ήπιο ρεύμα** ή **δυνατό ρεύμα**. Σχετικά με την **ορατότητα** μπορείτε να επιλέξετε μια από τις παρακάτω κατηγορίες: **Διαυγές νερό**, **μερικά σωματίδια,** ή **θολό νερό**.

Παρακαλούμε συμπληρώστε στο σχετικό πεδίο του πίνακα την ένταση του ρεύματος και την ορατότητα στο σημείο παρατήρησης.

2. <u>Χαρακτηριστικά τοποθεσίας και κοραλλιγενούς κοινότητας</u>

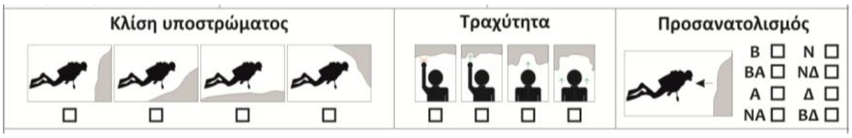

Η κλίση, η τραχύτητα και ο προσανατολισμός του υποστρώματος σε συνδυασμό με το βάθος αποτελούν παράγοντες που καθορίζουν τη σύνθεση των κοραλλιγενών κοινοτήτων.

Οι οργανισμοί υπόκεινται σε διαφορετικές συνθήκες φωτεινότητας και απόθεση ιζήματος ανάλογα με την **κλίση του υποστρώματος** στο οποίο ζουν. Το πρωτόκολλο παρέχει τέσσερις σχετικές κατηγορίες από τις οποίες μπορείτε να επιλέξετε (από αριστερά προς τα δεξιά της σχετικής εικόνας του πρωτοκόλλου**): 'κάθετο', 'με κλίση', 'οριζόντιο'** ή **'σε προεξοχή / με αρνητική κλίση'** υπόστρωμα. Παρακαλούμε σημειώστε στη σχετική εικόνα του πρωτοκόλλου την κλίση που αντιστοιχεί στην τοποθεσία παρατήρησης.

Η **τραχύτητα** αφορά στο πόσο ανώμαλος είναι ο βραχώδης πυθμένας. Όσο μεγαλύτερη τραχύτητα παρουσιάζει το υπόστρωμα, τόσο μεγαλύτερη είναι και η δομική του πολυπλοκότητα. Παρακάτω, παρουσιάζεται ένας πρακτικός τρόπος για να κατηγοριοποιηθεί η τραχύτητα σύμφωνα με τη μορφολογία του υποστρώματος (από αριστερά προς τα δεξιά της σχετικής εικόνας του πρωτοκόλλου). Οι σχισμές και οι τρύπες του υποστρώματος είναι: (1) **πολύ μικρές για να χωρέσουν τη γροθιά του δύτη**, (2) **αρκετά μεγάλες για να χωρέσουν τη γροθιά**, (3) **το κεφάλι** ή (4) **τους ώμους** του δύτη. Πολλές φορές είναι αρκετά δύσκολο να βρεθεί μόνο μια από τις παραπάνω κατηγορίες τραχύτητας σε μια τοποθεσία λόγω ταυτόχρονης παρουσίας περισσότερων της μιας κατηγοριών. Σε αυτή την περίπτωση, θα πρέπει να επιλέγεται η περιγραφή (κατηγορία) της συνηθέστερης κατάστασης, σε ότι αφορά την κλίση και την τραχύτητα, στη συνολική επιφάνεια των σχηματισμών που βρίσκονται στην τοποθεσία που παρατηρείτε. Παρακαλούμε σημειώστε στο πρωτόκολλο την κατηγορία τραχύτητας που περιγράφει αντιπροσωπευτικότερα την τοποθεσία που παρατηρείτε.

Ο **προσανατολισμός** περιγράφει τη διεύθυνση (γωνία ως προς το Βορρά) του υποστρώματος. Για την καταγραφή του προσανατολισμού του βραχώδους βυθού θα πρέπει να τοποθετηθείτε ακριβώς απέναντί του (αντικριστά) και να πάρετε διόπτευση στοχεύοντας με πυξίδα προς αυτόν. Προφανώς, τα οριζόντια υποστρώματα δεν έχουν προσανατολισμό. Σε περίπτωση που ο προσανατολισμός του υποστρώματος στην περιοχή παρατήρησης μεταβάλλεται, παρακαλούμε σημειώστε στο πρωτόκολλο τον προσανατολισμό που περιγράφει αντιπροσωπευτικότερα την τοποθεσία που παρατηρείτε.

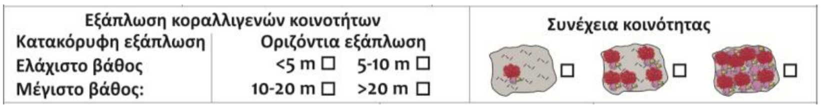

Η συνολική κατακόρυφη εξάπλωση των κοραλλιγενών κοινοτήτων είναι πολλές φορές δύσκολο να παρατηρηθεί αφού μπορεί να ξεπερνά κατά πολύ το μέγιστο βάθος της κατάδυσης σας. Για τον λόγο αυτό, το πεδίο του πρωτοκόλλου **'Εύρος βάθους παρατήρησης'** αναφέρεται στο ελάχιστο και το μέγιστο βάθος (σε μέτρα) στο οποίο παρατηρήσατε κοραλλιγενείς κοινότητες. Με άλλα λόγια, μπορείτε είτε να καταγράψετε το μέγιστο βάθος μέχρι το οποίο εξαπλώνονται οι κοραλλιγενείς κοινότητες (το οποίο μπορεί να είναι και μεγαλύτερο από το μέγιστο βάθος της κατάδυσης σας στην τοποθεσία παρατήρησης) είτε απλά να σημειώσετε το μέγιστο βάθος της κατάδυσης σας στην τοποθεσία παρατήρησης.

Η **οριζόντια εξάπλωση** αναφέρεται στην οριζόντια έκταση (σε μέτρα) που καταλαμβάνουν οι κοραλλιγενείς κοινότητες στην τοποθεσία που παρατηρείτε. Μπορείτε να επιλέξετε μεταξύ των παρακάτω κατηγοριών έκτασης: **<5 μ, 5-10 μ, 10-20 μ** ή **>20 μ.**

Η **συνέχεια της κοινότητας** έχει ως στόχο να περιγράψει εάν η κοραλλιγενής κοινότητα (1) εξαπλώνεται σε ένα μεμονωμένο τμήμα του υποστρώματος που περιβάλλεται από άλλους τύπους κοινοτήτων, γυμνό βράχο ή ίζημα, (2) παρουσιάζει ασυνέχειες, δηλαδή η εξάπλωση της κοινότητας διακόπτεται σποραδικά από άλλους τύπους κοινοτήτων, γυμνό βράχο ή ίζημα, ή (3) είναι συνεχής χωρίς αξιοσημείωτες ασυνέχειες. Συμπληρώστε από αριστερά προς τα δεξιά στο σχετικό πεδίο του πρωτοκόλλου.

3. Πιέσεις στις κοραλλιγενείς κοινότητες

Όλες οι ανθρωπογενείς ή φυσικές πιέσεις μπορούν να θέσουν σε κίνδυνο την επιβίωση των ειδών αλλά και τη σταθερότητα των κοραλλιγενών κοινοτήτων. Σε ορισμένες περιπτώσεις είναι δύσκολο να αποτραπούν ή να αποκατασταθούν οι αρνητικές επιδράσεις των πιέσεων (π.χ. εισβολικά είδη). Ωστόσο, η παρακολούθηση/καταγραφή των πιέσεων μπορεί να βοηθήσει στη διαχείριση τους αλλά και στη μείωση των αρνητικών συνεπειών που μπορούν να επιφέρουν (π.χ. ελεγχόμενη αγκυροβολία, περιορισμός αλιευτικής πίεσης, υπεύθυνη κατάδυση αναψυχής).

Κατά τη διάρκεια της κατάδυσής σας, μπορείτε να εκτιμήσετε τις **πιέσεις** σύμφωνα με την κρίση σας. Έτσι, μπορείτε να εκτιμήσετε την ένταση των πιέσεων που παρουσιάζονται παρακάτω σύμφωνα με τις εξής κατηγορίες: **0 = απούσα, + = σπάνια, ++ = άφθονη.**

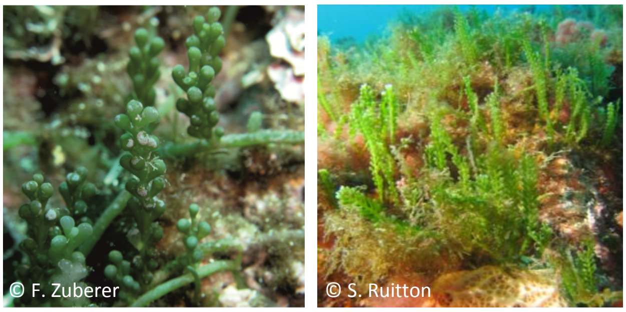

Caulerpa cylindracea

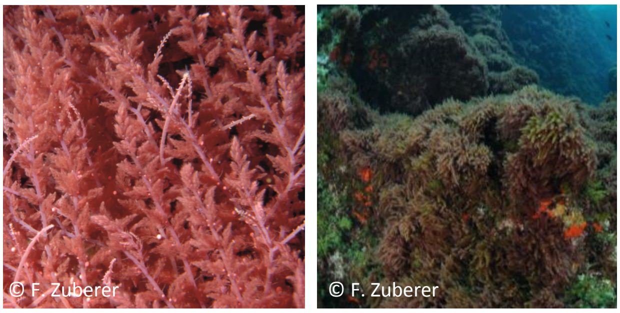

Asparagopsis spp.

Αυτά τα είδη μακροφυκών έχουν δυο κοινά χαρακτηριστικά: είναι **αλλόχθονα**, δηλαδή έχουν προέλευση από άλλες θαλάσσιες περιοχές εκτός της Μεσογείου, και είναι επίσης **εισβολικά** που σημαίνει ότι μπορούν να εξαπλωθούν και να ανταγωνιστούν τα αυτόχθονα είδη. Είναι ιδιαίτερα σημαντικό να παρακολουθείται/καταγράφεται η παρουσία και η αφθονία (χονδρική εκτίμηση) των ειδών αυτών, καθώς μπορούν να απειλήσουν την τοπική βιοποικιλότητα και τη σταθερότητα των οικοσυστημάτων.

Περισσότερες φωτογραφίες: *C. cylindracea*, *Asparagopsis* spp.

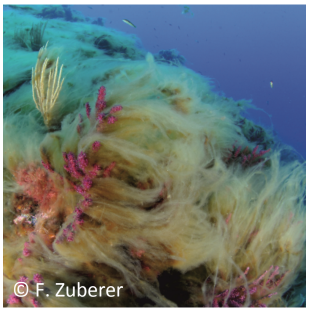

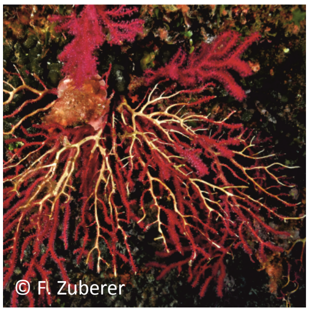

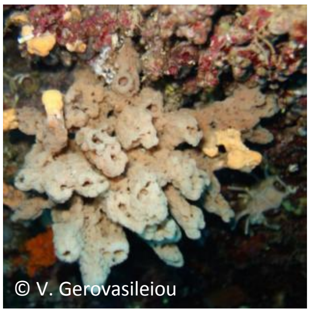

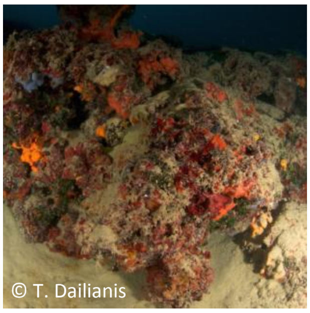

Ζελατινώδειςσυναθροίσεις   Νέκρωση/Περιστατικά μαζικής θανάτωσης   Απόθεση ιζήματος

Ζελατινώδεις συναθροίσεις: σχηματίζονται από μικροσκοπικά φύκη με χαρακτηριστική ζελατινώδη υφή και νηματοειδή εμφάνιση, συνήθως υποκίτρινου χρώματος. Οι ζελατινώδεις συναθροίσεις μπορούν να αναπτύσσονται υπέρμετρα σε συγκεκριμένες συνθήκες θερμοκρασίας και κυκλοφορίας του νερού (π.χ. ρεύματα) με αποτέλεσμα να καλύπτουν εξολοκλήρου τον θαλάσσιο πυθμένα και να προκαλούν ασφυξία στους βενθικούς οργανισμούς.

Η παρουσία των ζελατινωδών συναθροίσεων, σε συνδυασμό με ιδιαίτερα αυξημένες τιμές θερμοκρασίας για μεγάλα χρονικά διαστήματα, μπορεί να προκαλέσει μερική ή ολική **νέκρωση** των οργανισμών ή ακόμα και **περιστατικά μαζικής θανάτωσης** ολόκληρων πληθυσμών (όλα τα άτομα που ανήκουν στο ίδιο είδος).
Η εκτεταμένη **απόθεση ιζήματος** μπορεί να έχει είτε φυσική προέλευση, για παράδειγμα ως αποτέλεσμα της έκπλυσης της ξηράς εξαιτίας ισχυρών βροχοπτώσεων, είτε να είναι αποτέλεσμα ανθρώπινων δραστηριοτήτων, όπως οι κατασκευές, οι εξορύξεις και η εκροή επεξεργασμένων υδάτων στις παράκτιες περιοχές. Ασχέτως από την πηγή προέλευσης, η εκτεταμένη απόθεση ιζήματος οδηγεί στο 'θάψιμο' των οργανισμών και την υποβάθμιση των κοινοτήτων.

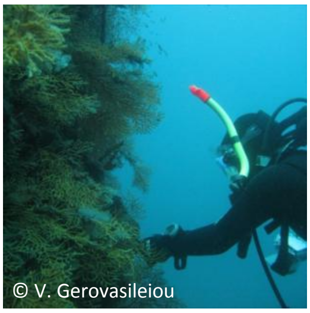

Απροσεξία δυτών

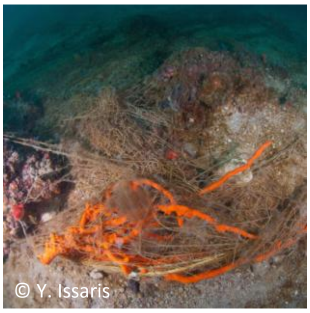

Αλιευτικά εργαλεία

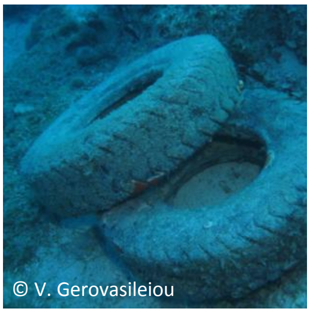

Απορρίμματα

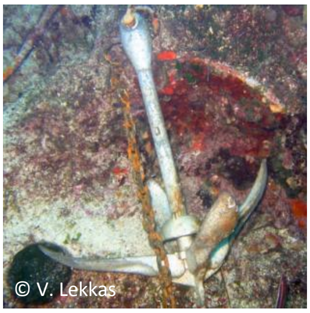

Αγκυροβολία

Τα **απορρίμματα**, τα εγκαταλειμμένα **αλιευτικά εργαλεία** και η **αγκυροβολία** μπορούν να είναι ιδιαίτερα επιβλαβή για τις κοραλλιγενείς κοινότητες σε τοπικό επίπεδο.

Η **απροσεξία των δυτών** μπορεί επίσης να έχει αρνητικές συνέπειες στις κοραλλιγενείς κοινότητες. Για παράδειγμα τα βρυόζωα είναι ιδιαίτερα ευαίσθητα στη μηχανική όχληση που προκαλείται από την επαφή των δυτών (π.χ. με τα χέρια, πέδιλα ή οποιοδήποτε εξάρτημα του εξοπλισμού). Για το λόγο αυτό, θεωρούνται ως δείκτες της καταδυτικής έντασης που δέχεται μια περιοχή. Σε περιοχές με μεγάλη καταδυτική επισκεψιμότητα μπορούν να βρεθούν ολόκληρες αποικίες από βρυόζωα αποκολλημένες από τη φυσική τους θέση.

4. Τα είδη

Η παρακάτω λίστα περιλαμβάνει έναν περιορισμένο αριθμό ειδών, τα οποία επιλέχτηκαν σύμφωνα με: (1) το ρόλο που παίζουν στις κοραλλιγενείς κοινότητες (π.χ. βιοκατασκευαστές ή βιοαποδομητές), (2) τη χαρακτηριστική παρουσία τους στις κοραλλιγενείς κοινότητες ή τη χρήση τους ως δεικτών για την εκτίμηση της οικολογικής ποιότητας αυτών και (3) το καθεστώς προστασίας τους από την Εθνική και Διεθνή νομοθεσία. Η συλλογή πληροφορίας για την παρουσία και αφθονία των ειδών αυτών συνεισφέρει στην κατανόηση και περιγραφή των κοραλλιγενών κοινοτήτων και την εκτίμηση της οικολογικής τους κατάστασης.

Για την **εκτίμηση της αφθονίας** κάθε είδους μπορείτε να επιλέξετε μια από τις παρακάτω κατηγορίες: **0 = απόν, + = σπάνιο, ++ = άφθονο, +++ = πολύ άφθονο**.

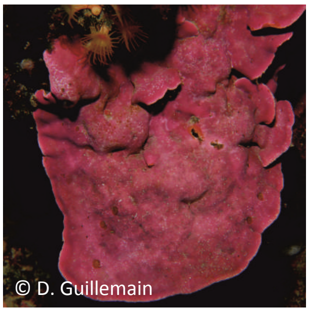

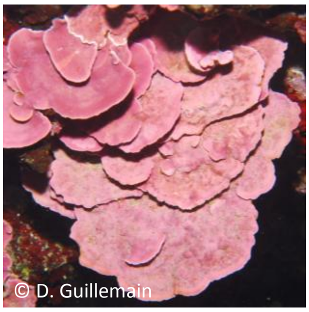

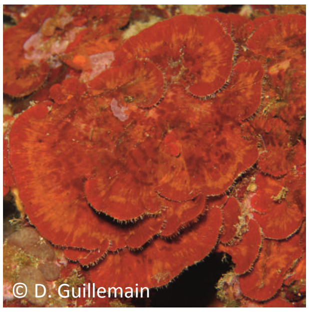

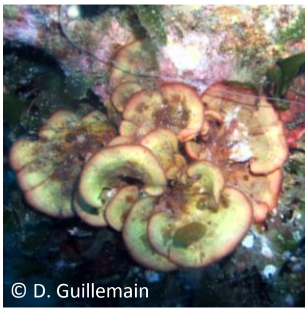

Ενασβεστωμένα ροδοφύκη

Peyssonnelia spp.

Τα φύκη αυτά αποτελούν τους κυριότερους εκπροσώπους της χλωρίδας των κοραλλιγενών κοινοτήτων. Τα **ενασβεστωμένα ροδοφύκη**, *Lithophyllum stictaeforme/cabiochiae* και *Mesophyllum alterans* είναι από τους κυριότερους βιοκατασκευαστές των κοραλλιγενών κοινοτήτων. Τα μακροφύκη του γένους ***Peyssonnelia*** περιλαμβάνουν τόσο ενασβεστωμένα (*Peyssonnelia rosa-marina*) όσο και μη ενασβεστωμένα (*Peyssonnelia squamaria)* είδη.

Περισσότερες φωτογραφίες: *L. stictaeforme/cabiochiae*, *M. expansum*, *P. rosa-marina*, *P. squamaria*.

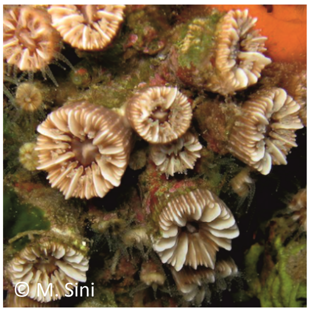

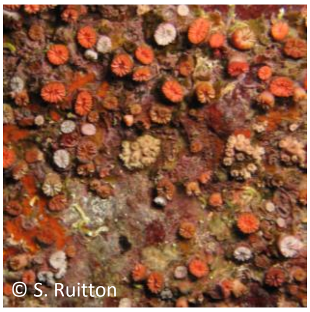

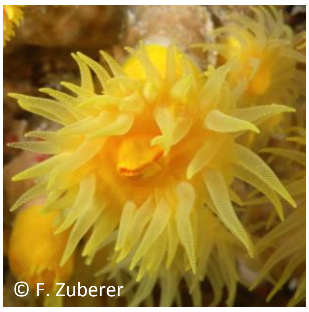

Σκληρακτίνια κοράλλια

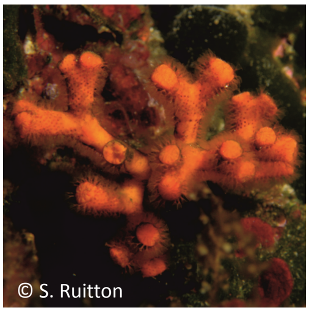

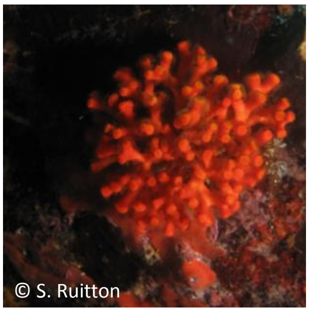

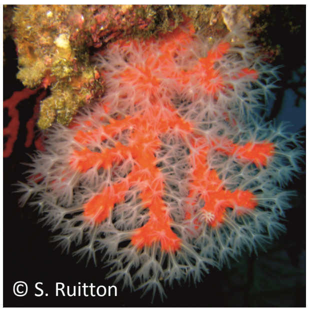

Myriapora truncata

Corallium rubrum

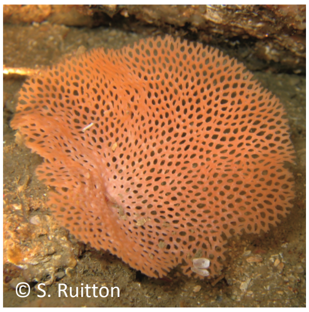

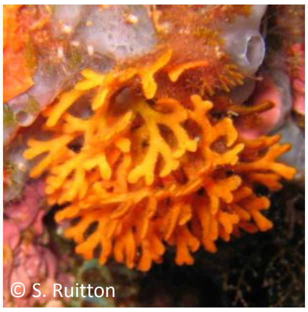

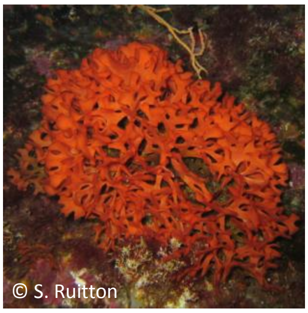

Άλλα βρυόζωα

Τα **σκληρακτίνια κοράλλια (Scleractinia)** και τα βρυόζωα (***Myriapora truncata*** και **άλλα είδη βρυοζώων**) - γνωστά και ως ψευδοκόραλλα - διαθέτουν ασβεστώδη σκελετό και αποτελούν δευτερεύοντες βιοκατασκευαστές των κοραλλιγενών σχηματισμών. Τα βρυόζωα είναι εύθραυστα και συνεπώς ιδιαίτερα ευαίσθητα στη μηχανική όχληση. Χρησιμοποιούνται ως δείκτες έντασης της καταδυτικής δραστηριότητας σε μια περιοχή.
Το κόκκινο κοράλλι (***Corallium rubrum***), που δεν πρέπει να συγχέεται με το βρυόζωο *M. truncata*, θεωρείται δευτερεύων βιοκατασκευαστής των κοραλλιγενών κοινοτήτων και η συλλογή του ρυθμίζεται από τη σχετική νομοθεσία. Είναι πιο κοινό στη Δυτική Μεσόγειο.

Περισσότερες φωτογραφίες: Σκληρακτίνια κοράλλια - *Caryophyllia (Caryophyllia) smithii*, *Caryophyllia inornata*, *Hoplangia durotrix*, *Leptopsammia pruvoti*, *Madracis pharensis*, *Phyllangia mouchezii*, *Polycyathus muellerae*. Βρυόζωο *M. truncata*. Κόκκινο κοράλλι *C. rubrum*. Άλλα βρυόζωα - *Adeonella calveti*, *Pentapora fascialis*, *Smittina cervicornis*.

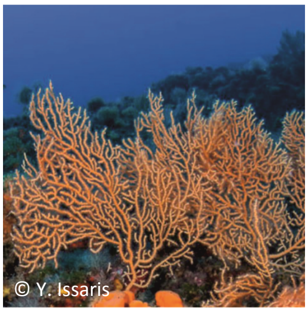

Eunicella cavolini

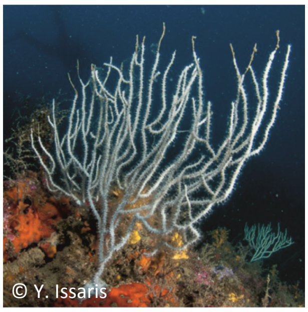

Eunicella singularis

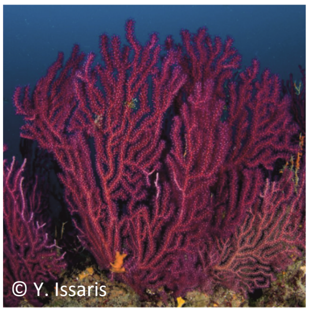

Paramuricea clavata

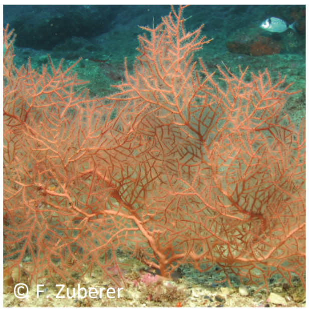

Leptogorgia sarmentosa

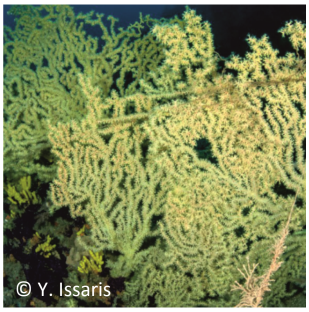

Savalia savaglia

Τα είδη με δενδροειδή μορφή παρουσιάζουν ιδιαίτερο ενδιαφέρον καθώς αυξάνουν τη δομική πολυπλοκότητα του οικοτόπου. Οι κίτρινες (***Eunicella cavolini***), άσπρες (***Eunicella singularis***) και μωβ γοργονίες (***Paramuricea calvata***) είναι και οι πιο κοινές. Η πορτοκαλί γοργονία (***Leptogorgia sarmentosa***) προτιμά θολά νερά και έτσι θεωρείται δείκτης θολερότητας των υδάτων.
Η ***Savalia savaglia*** (ψευδογιούσουρι) είναι σπάνια και απαντά σε σχετικά βαθύτερα νερά. Η αναφορά του είδους αυτού είναι ιδιαίτερα σημαντική για την εκτίμηση της κατανομής του.

Πρόσφατα, η μωβ γοργονία (*P. clavata*) συμπεριλήφθηκε στην Κόκκινη Λίστα Απειλούμενων Ειδών της IUCN ως τρωτό είδος· οι γοργονίες *E. cavolini*, *E. singularis* και το ψευδογιούσουρι (*S. savaglia*) συμπεριλήθφηκαν στην Κόκκινη Λίστα ως σχεδόν απειλούμενα.

Περισσότερες φωτογραφίες: *E. cavolini*, *E. singularis*, *P. clavata*, *L. sarmentosa*, *S. savaglia*.

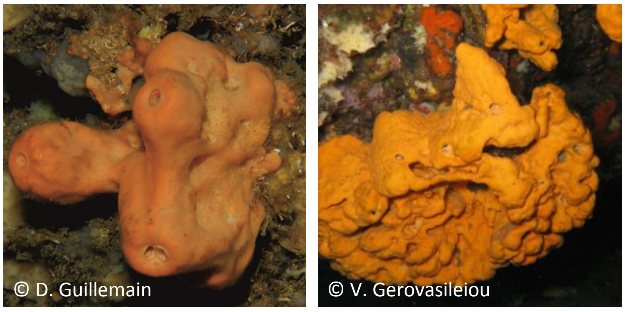

Agelas oroides

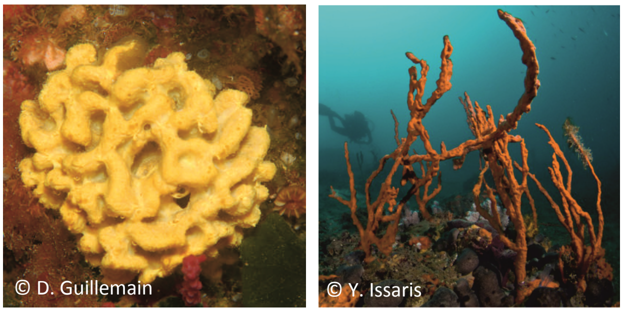

Axinella spp.

Οι σπόγγοι αναπτύσσουν σημαντικούς πληθυσμούς στις κοραλλιγενείς κοινότητες, ειδικά στην ανατολική Μεσόγειο (π.χ. Αιγαίο και Ιόνιο). Έτσι, επιλέχτηκαν τα πιο κοινά Μεσογειακά είδη: ***Agelas oroides*** και τα είδη του γένους ***Axinella*** (*Axinella cannabina, A. damicornis, A. verrucosa, A. polypoides*, όλα προστατευόμενα).

Περισσότερες φωτογραφίες: *A. oroides*, *A. damicornis*, *A. verrucosa*, *A. polypoides*.

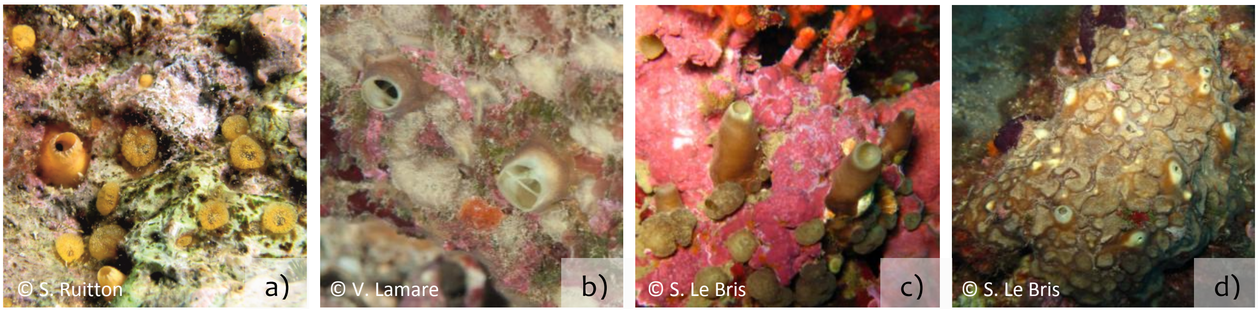

Cliona spp.

Οι σπόγγοι του γένους ***Cliona*** παρουσιάζουν ιδιαίτερο ενδιαφέρον καθώς διατρυπούν τα ασβεστώδη υποστρώματα. Αποτελούν τους κυριότερους βιοαποδομητές των κοραλλιγενών σχηματισμών. Στα πρώτα στάδια της ζωής τους έχουν τη μορφή 'θηλών' (Εικόνες a και b) ή μικρών 'καμινάδων' (Εικόνα c) ενώ σε πιο προχωρημένα στάδια ανάπτυξης μπορούν να αποκτήσουν συμπαγή μορφή (Εικόνα d).

Περισσότερες φωτογραφίες: *C. celata*, *C. shmidtii*, *C. viridis*.

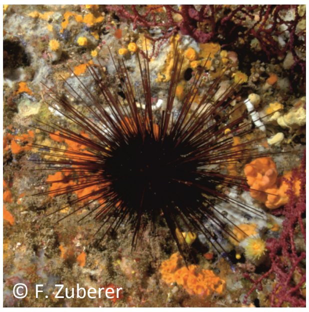

Centrostephanus longispinus

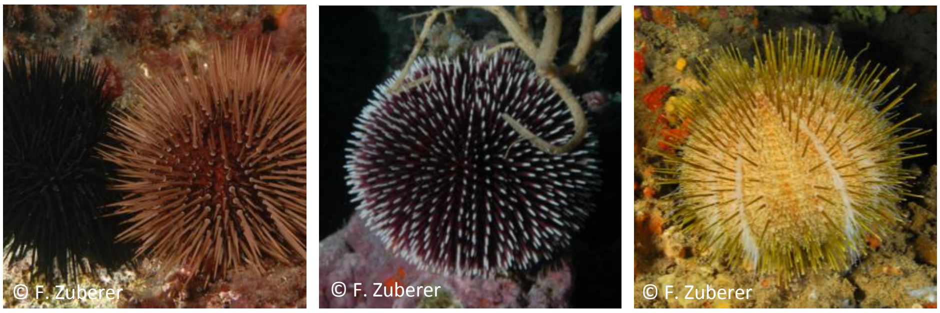

Άλλα είδη αχινών

Οι αχινοί «βόσκουν» τα ενασβεστωμένα ροδοφύκη, συνεισφέροντας έτσι στην αποδόμηση του υποστρώματος (βιοαποδομητές). Ο ***Centrostephanus longispinus*** είναι ιδιαίτερα ενεργός βοσκητής και αποτελεί προστατευόμενο είδος. **Άλλα είδη αχινών** (π.χ. *Echinus melo, Gracilechinus acutus, Sphaerechinus granularis, Stylocidaris affinis, Paracentrotus lividus, Arbacia lixula*) συνεισφέρουν επίσης στην αποδόμηση του βιογενούς υποστρώματος, αν και σε μικρότερο βαθμό.

Περισσότερες φωτογραφίες: *C. longispinus*, *E. melo*, *G. acutus*, *S. granularis*, *S. affinis*, *P. lividus*, *A. lixula*.

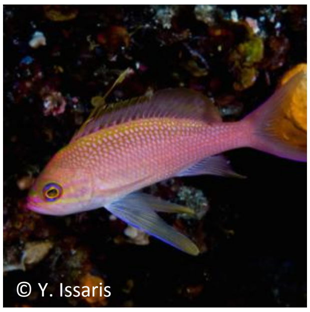

Anthias anthias

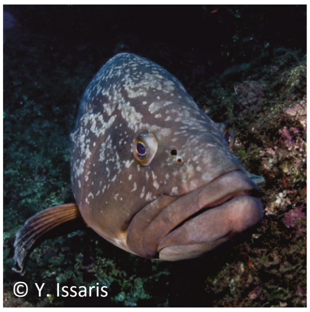

Epinephelus marginatus

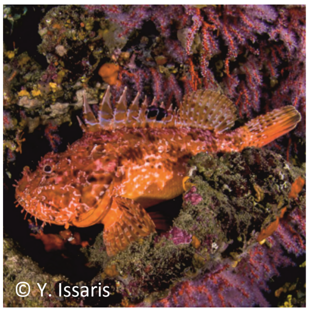

Scorpaena spp.

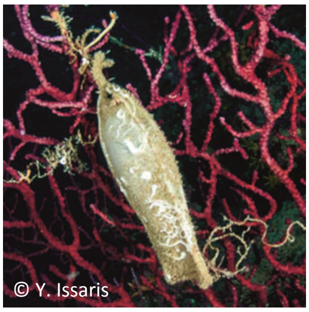

Αυγά σκυλόψαρων

Ο Ανθίας ή κοκκινόχανος (***Anthias anthias***), ο ροφός (***Epinephelus marginatus***) και τα σκορπίδια (***Scorpaena* spp.**) είναι κοινά στις κοραλλιγενείς κοινότητες. Ο ροφός θεωρείται παγκοσμίως κινδυνεύον είδος καθώς οι πληθυσμοί του έχουν υποστεί δραματική μείωση τις περασμένες δεκαετίες και η ερασιτεχνική αλιεία του περιορίζεται από τη σχετική νομοθεσία. Τα είδη του γένους *Scorpaena* (σκορπίδια) παρουσιάζουν σημαντικό αλιευτικό ενδιαφέρον. Οι **καρχαρίες** και τα σελάχια συχνά αποθέτουν τα **αυγά** τους στα κλαδιά των γοργονιών, χρησιμοποιώντας τις κοραλλιγενείς κοινότητες ως πεδίο αναπαραγωγής και νηπιοτροφείο.

Περισσότερες φωτογραφίες: *A. anthias*, *E. marginatus*, *Scorpaena maderensis*, *S. notata*, *S. porcus*, *S. scrofa*. Αυγά σκυλόψαρων.

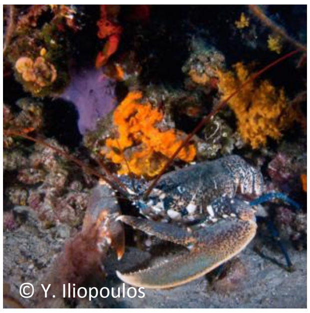

Homarus gammarus

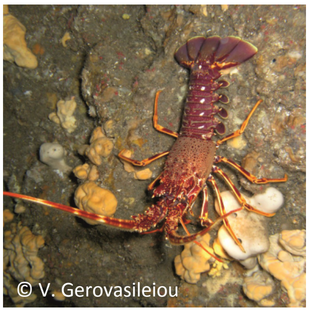

Palinurus elephas

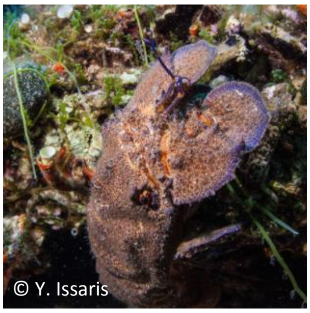

Scyllarides latus

Τα είδη αυτά των καρκινοειδών που απαντούν συχνά στις κοραλλιγενείς κοινότητες παρουσιάζουν μεγάλη εμπορική αξία. Για το λόγο αυτό, η συλλογή της αστακοκαραβίδας (***Homarus gammarus***), του αστακού (***Palinurus elephas***) και της κωλοχτύπας (***Scylarides latus***) ρυθμίζεται από τη σχετική νομοθεσία.

Περισσότερες φωτογραφίες: *H. gammarus*, *P. elephas*, *S. latus*.

Ήρθε η ώρα για κατάδυση, τι πρέπει να κάνω;

Μπορείτε να σημειώσετε τις παρατηρήσεις σας στον υποβρύχιο πίνακα (πρωτόκολλο) που σας παρέχουμε με ένα μολύβι. Η σειρά με την οποία θα μπορούσατε να συμπληρώσετε τον πίνακα είναι η εξής: από πάνω προς τα κάτω και από αριστερά προς τα δεξιά. Ο λόγος για να ακολουθήσετε τη σειρά αυτή είναι ότι ο πίνακας έχει σχεδιαστεί με τέτοιο τρόπο ώστε να βελτιστοποιεί την παρατήρησή σας. Για παράδειγμα, η σειρά παρατήρησης των ειδών σχετίζεται με το μέγεθος και άρα με την ευκολία εντοπισμού τους: τα μεγάλα και ευδιάκριτα είδη είναι πρώτα, ενώ διαδοχικά ακολουθούν μικρότερα είδη που απαιτούν μια πιο κοντινή ματιά ή είδη που ζουν σε τρύπες και κοιλότητες. Αντίστοιχα, η θερμοκρασία του νερού στο βάθος παρατήρησης βρίσκεται στο τέλος έτσι

ώστε να παρέχεται αρκετός χρόνος στον υπολογιστή κατάδυσης (dive computer) για να καταγράψει με ακρίβεια τη θερμοκρασία.

Κανένα πεδίο του πίνακα δεν είναι υποχρεωτικό αλλά συνιστάται να μην ξεχάσετε να σημειώσετε το βάθος της παρατήρησης.

ΑΠΑΡΑΙΤΗΤΟΣ ΕΞΟΠΛΙΣΜΟΣ: ΠΙΝΑΚΑΣ (ΠΡΩΤΟΚΟΛΛΟ), ΦΑΚΟΣ, ΥΠΟΛΟΓΙΣΤΗΣ ΚΑΤΑΔΥΣΗΣ, ΠΥΞΙΔΑ

ΠΡΟΑΙΡΕΤΙΚΟΣ ΕΞΟΠΛΙΣΜΟΣ: GPS, ΥΠΟΒΡΥΧΙΑ ΦΩΤΟΓΡΑΦΙΚΗ ΜΗΧΑΝΗ

Συμπλήρωση του πρωτοκόλλου βήμα προς βήμα:

1. Βεβαιωθείτε ότι το μολύβι με το οποίο θα συμπληρώσετε το πρωτόκολλο είναι εντάξει (ένα εφεδρικό μολύβι θα ήταν επίσης χρήσιμο).
2. Σημειώστε την ημερομηνία και την περιοχή κατάδυσης (δώστε συντεταγμένες από GPS αν είναι δυνατό).
3. Κατά την κάθοδό σας σημειώστε το βάθος στο οποίο νιώσατε απότομη πτώση της θερμοκρασίας (αν συμβεί αυτό).
4. Μόλις φτάσετε στο βάθος που θέλετε, επιλέξτε την περιοχή που θα πραγματοποιηθεί η παρατήρηση: θα μπορούσε να είναι μια περιορισμένη επιφάνεια (όχι όμως μικρότερη σε μήκος και πλάτος από το άνοιγμα των χεριών σας) ή μια μικρή διαδρομή σε σταθερό βάθος.
5. Σημειώστε: το βάθος παρατήρησης, το ρεύμα, την ορατότητα, το παρατηρούμενο εύρος βάθους, την οριζόντια έκταση, τη συνέχεια/ασυνέχεια της κοινότητας, την κλίση, την τραχύτητα και τον προσανατολισμό.
6. Παρατηρήστε αν υπάρχουν εισβολικά είδη ή άλλες πιέσεις/απειλές και σημειώστε την αφθονία και την έντασή τους, σύμφωνα με τις κατηγορίες που δίνονται.
7. Παρατηρήστε τα είδη και εκτιμήστε την αφθονία τους σύμφωνα με τις κατηγορίες που δίνονται.
8. Σημειώστε τη θερμοκρασία του νερού και το βάθος στο οποίο πραγματοποιήθηκε η παρατήρηση.
9. Μετά την κατάδυση μπορείτε να ανεβάσετε τις παρατηρήσεις και τις φωτογραφίες σας στην ιστοσελίδα της δράσης '*CIGESMED για δύτες, πολίτες-επιστήμονες*' (http://cs.cigesmed.eu).

   Εφόσον ο υπολογιστής κατάδυσης (dive computer) που χρησιμοποιήσατε καταγράφει τη θερμοκρασία του νερού κατά τη διάρκεια της κατάδυσης, σημειώστε τη θερμοκρασία του νερού σε προκαθορισμένα βάθη (κάθε 10 μέτρα).

   Μετά την επιτυχή δήλωση της υποβρύχιας καταγραφής σας στην ιστοσελίδα, φωτογραφήστε το συμπληρωμένο πίνακα που χρησιμοποιήσατε και αποθηκεύστε τη φωτογραφία στο αρχείο σας. Στη συνέχεια μπορείτε να σβήσετε τα συμπληρωμένα πεδία και να χρησιμοποιήσετε τον πίνακα ξανά όσες φορές επιθυμείτε.

Όνομα

Τοποθεσία

Ημερομηνία

Σε ποιο βάθος αισθανθήκατε σημαντική πτώση της θερμοκρασίας? m / ποτέ

Βάθος παρατήρησης:

Ένταση του ρεύματος
Απουσία ☐ Ήπιο ☐ Δυνατό ☐

Ορατότητα
Διαυγές νερό ☐ Μερικά σωματίδια ☐ Θολά ☐

Εξάπλωση κοραλλιγενών κοινοτήτων

Κατακόρυφη εξάπλωση
Ελάχιστο βάθος
Μέγιστο βάθος:

Οριζόντια εξάπλωση
<5 m ☐ 5-10 m ☐
10-20 m ☐ >20 m ☐

Συνέχεια κοινότητας

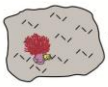 ☐ 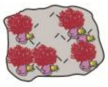 ☐ 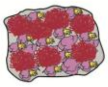 ☐

Κλίση υποστρώματος

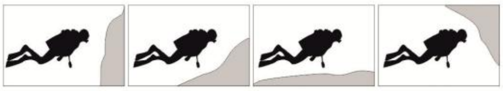

☐ ☐ ☐ ☐

Τραχύτητα

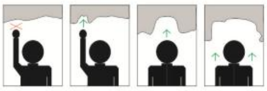

☐ ☐ ☐ ☐

Προσανατολισμός

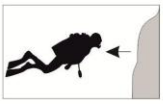

Β ☐ Ν ☐
ΒΑ ☐ ΝΔ ☐
Α ☐ Δ ☐
ΝΑ ☐ ΒΔ ☐

Πιέσεις και απειλές

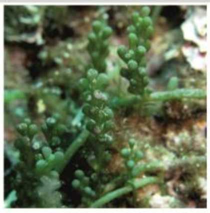

Caulerpa cylindracea

0 + ++
☐ ☐ ☐

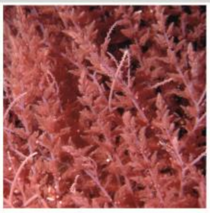

Asparagopsis spp.

0 + ++
☐ ☐ ☐

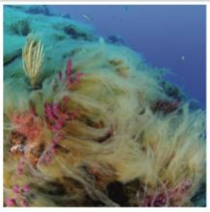

Ζελατινώδεις συναθροίσεις

0 + ++
☐ ☐ ☐

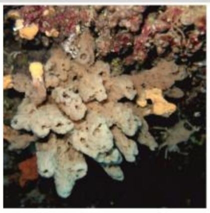

Νέκρωση/περιστατικά μαζικής θανάτωσης

0 + ++
☐ ☐ ☐

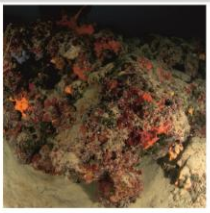

Απόθεση ιζήματος

0 + ++
☐ ☐ ☐

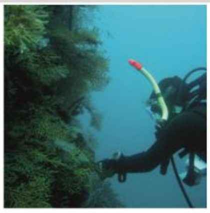

Απροσεξία δυτών

0 + ++
☐ ☐ ☐

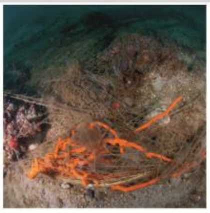

Αλιευτικά εργαλεία

0 + ++
☐ ☐ ☐

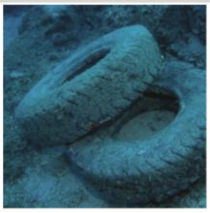

Απορρίμματα

0 + ++
☐ ☐ ☐

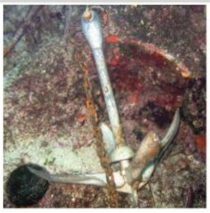

Αγκυροβολία

0 + ++
☐ ☐ ☐

0 = Απούσα
+ = Σπάνια
++ = Άφθονη

Άλλες παρατηρήσεις

Οργανισμοί κοραλλιγενών κοινοτήτων

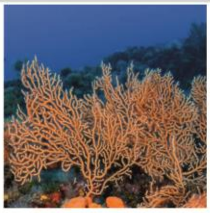
Eunicella cavolini
0 + ++ +++

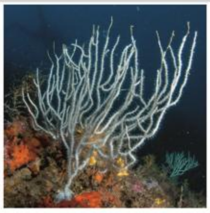
Eunicella singularis
0 + ++ +++

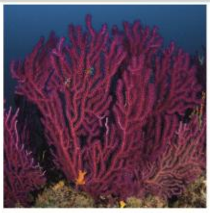
Paramuricea clavata
0 + ++ +++

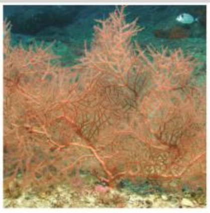
Leptogorgia sarmentosa
0 + ++ +++

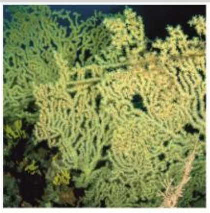
Savalia savaglia
0 + ++ +++

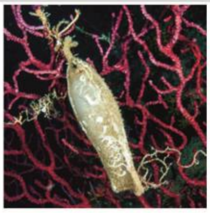
Αυγά σκυλόψαρων
0 + ++ +++

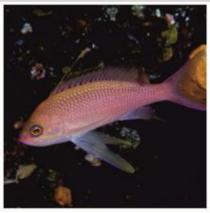
Anthias anthias
0 + ++ +++

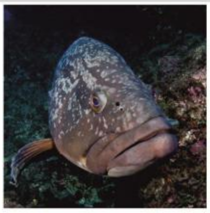
Epinephelus marginatus
0 + ++ +++

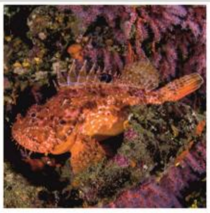
Scorpaena spp.
0 + ++ +++

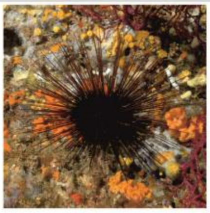
Centrostephanus longispinus
0 + ++ +++

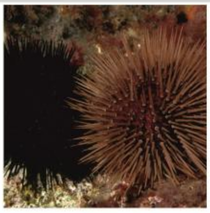
Άλλα είδη αχινών
0 + ++ +++

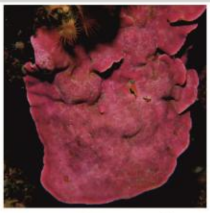
Ενασβεστωμένα ροδοφύκη
0 + ++ +++

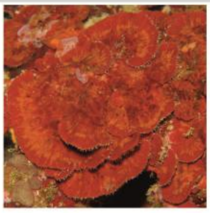
Peyssonnelia spp.
0 + ++ +++

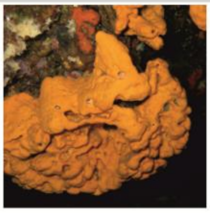
Agelas oroides
0 + ++ +++

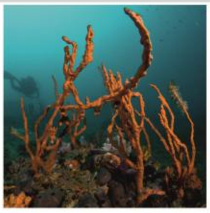
Axinella spp.
0 + ++ +++

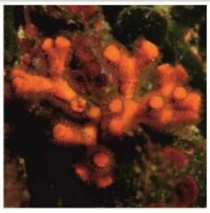
Myriapora truncata
0 + ++ +++

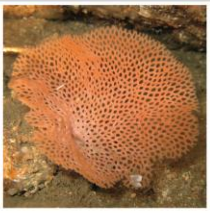
Άλλα βρυόζωα
0 + ++ +++

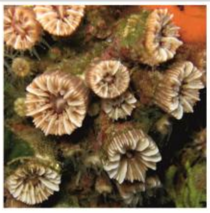
Σκληρακτίνια κοράλλια
0 + ++ +++

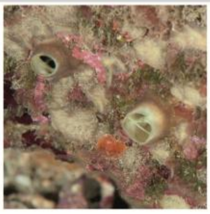
Cliona spp.
0 + ++ +++

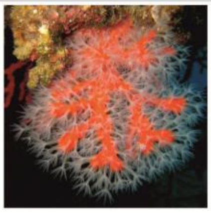
Corallium rubrum
0 + ++ +++

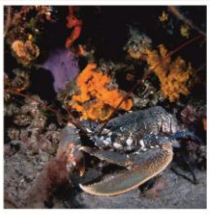
Homarus gammarus
0 + ++ +++

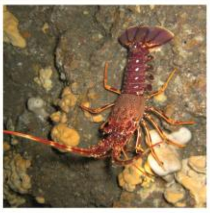
Palinurus elephas
0 + ++ +++

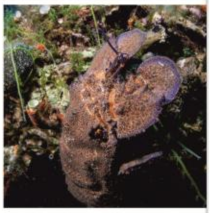
Scyllarides latus
0 + ++ +++

Θερμοκρασία νερού στο βάθος παρατήρησης:

0 = Απόν + = Σπάνιο
++ = Άφθονο +++ = Πολύ άφθονο

Citizen Science 4 CIGESMED

Guidelines to be cited as: Gatti G., Thierry de Ville d'Avray L., David R., Dimitriadis C., Gerovasileiou V., Dailianis T., Sini M., Salomidi M., Dogan A., Issaris Y., Çinar M.E., Koutsoubas D., Arvanitidis C., Feral J-P. 2015. CIGESMED for divers - Citizen Science for CIGESMED. SeasEra project (E.U. FP7 ERA-NET). 12 p.
