# Supplementary material for: CIGESMED for divers: Establishing a citizen science initiative for the mapping and monitoring of coralligenous assemblages in the Mediterranean Sea
Source: Biodivers Data J. 2016 Nov 1;(4):e8692. doi: 10.3897/BDJ.4.e8692 (PMC5136673; doi:10.3897/BDJ.4.e8692)
Supplement: Supplementary material 4 — CIGESMED per i subacquei - Citizen science per CIGESMED [file biodiversity_data_journal-4-e8692-s004.pdf]

http://cs.cigesmed.eu/it

www.cigesmed.eu

CIGESMED per i subacquei – Citizen Science per CIGESMED

Che cos'è il "coralligeno"?

Quello che viene comunemente chiamato "coralligeno" è in realtà un complesso paesaggio sottomarino, che si sviluppa in condizioni di limitata luminosità ed esclusivamente nel mar Mediterraneo. L'habitat coralligeno è costruito principalmente da alghe rosse calcificate, che depositano strati di calcare sui fondali rocciosi da centinaia di anni: per questo motivo sono considerati l'equivalente mediterraneo delle scogliere coralline tropicali. All'azione costruttrice delle alghe si contrappone quella dei cosiddetti "erosori" - come le spugne del genere *Cliona* o i ricci di mare - ossia organismi che perforano e consumano il substrato calcareo, contribuendo ad aumentare la complessità strutturale del coralligeno. Tali substrati calcarei supportano numerose specie longeve (spugne, gorgonie, briozoi, coralli), che a loro volta contribuiscono a strutturare l'habitat coralligeno. Infine, un gran numero di invertebrati (nudibranchi, crostacei, ascidie, echinodermi, molluschi) e pesci abitano tale habitat, contribuendo ulteriormente a renderlo una riserva di biodiversità unica.

Perche osserviamo il coralligeno?

L'habitat coralligeno è unico al mondo ed è uno tra i più ricchi e bei paesaggi che si possano osservare in immersione subacquea. Grazie alla loro complessità, ospitano un gran numero di specie importanti dal punto di vista ecologico, estetico e commerciale, alcune delle quali sono protette da leggi nazionali ed internazionali. L'habitat coralligeno, pero', è spesso minacciato dal comportamento umano. L'ancoraggio delle barche, la subacquea irresponsabile, la (sovra)pesca, lo scarico di rifiuti, insieme al riscaldamento delle acque superficiali (causato dal cambiamento climatico) ed alle invasioni da parte di specie aliene, sono tra le principali minacce per lo stato di salute di questo habitat.

Partecipando alle attività di *CIGESMED for divers* contribuirai all'esplorazione ed alla conservazione dell'habitat coralligeno e dell'ambiente marino, e potrai approfondire le tue conoscenze sulla biodiversità marina.

Che cosa osserviamo?

1. Alcune informazioni generali sul sito

Temperatura dell'acqua alla profondità dell'osservazione:

La **temperature dell'acqua** è un fattore essenziale per la sopravvivenza delle specie, ed è quindi importante conoscerne il valore **alla profondità dell'osservazione**.

A che profondità hai incontrato l'acqua più fredda?	m / mai

Tale profondità corrisponde al limite inferiore del termoclino, ovvero la fascia di profondità caratterizzata da una brusca diminuzione della **temperatura** dell'acqua (transizione tra le acque superficiali, più calde, e quelle profonde, più fredde). Talvolta tale limite puó scendere a profondità superiori rispetto a quelle abituali, a causa di eccezionali ondate di calore. Di conseguenza, gli organismi abituati a vivere in condizioni fredde vengono a contatto con acque più calde; se tale situazione persiste, puó causare mortalità parziali o estese di numerosi organismi, tra i quali gorgonie e spugne.

Riempire lo spazio indicando la temperature alla profondità dell'osservazione e il limite inferiore del termoclino (A che profondità hai incontrato l'acqua più fredda?) in metri, oppure spuntare l'opzione 'mai' se non lo si ha incontrato.

Profondità della osservazione:	Corrente	Visibilità
	Assente☐ Debole☐ Forte☐	Acqua limpida☐ Qualche particella☐ Torbida☐

La **profondità** è un fattore essenziale nel determinare la distribuzione delle specie marine e la composizione dell'habitat. Indicare la profondità (in metri) alla quale è stata effettuata l' osservazione.

Alla profondità dell'osservazione, alcune informazioni riguardo l'intensità di corrente e la visibilità vanno a completare la descrizione generale del sito. Per la **corrente** è sufficiente scegliere tra: **assente, debole, forte.** Riguardo alla **visibilità**, ci sono nuovamente tre possibilità: **acqua limpida**, **acqua torbida**, ed una classe intermedia chiamata "**qualche particella in sospensione**".

Spuntare la casella corrispondente all'intensità di corrente ed alla visibilità osservate.

2. Caratteristiche del sito e dell'habitat

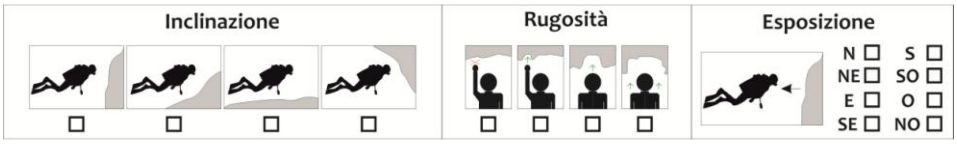

Insieme alla profondità, l'inclinazione del substrato, la rugosità ed l'esposizione sono tra i fattori che influenzano maggiormente la composizione del coralligeno.

A seconda dell'**inclinazione** del substrato, gli organismi sono esposti ad intensità luminose ed a tassi di sedimentazione differenti. E' possibile scegliere tra quattro categorie di inclinazione: **verticale**, **inclinato**, **orizzontale** e **strapiombante**.

La **rugosità** rappresenta l'irregolarità del substrato: più quest'ultimo è irregolare e più é complesso. Suggeriamo un metodo pratico per misurare la rugosità utilizzando dei riferimenti anatomici. Buchi e cavità sono: 1) **troppo piccoli perché ci si possa infilare un pugno dentro**, oppure 2) **abbastanza grandi da poterci infilare dentro un pugno**, 3) **la testa** o 4) **le spalle.** Nella maggior parte dei casi ci si troverà di fronte a cavità di differenti dimensioni: in tal caso, si dovrà scegliere la taglia più grande, perchè è presumibile che contenga delle cavità più piccole.

L'**esposizione** è la direzione verso la quale "guarda" il substrato. Per misurarla ci si pone di fronte alla parete con la bussola e si considera la direzione indicata dalla freccia immaginaria che esce dalla parete. Ovviamente, le superfici orizzontali non hanno alcuna esposizione.

Spuntare la casella corrispondente all'inclinazione, alla dimensione delle cavità ed all'esposizione osservate.

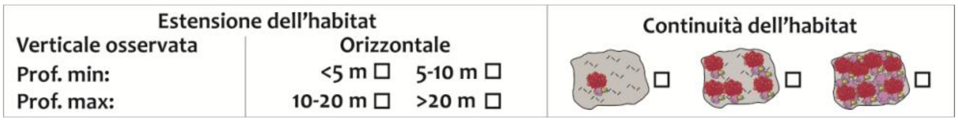

Estensione dell'habitat		Continuità dell'habitat
Verticale osservata	Orizzontale	
Prof. min:	<5 m ☐ 5-10 m ☐	☐ ☐ ☐
Prof. max:	10-20 m ☐ >20 m ☐	

Non è sempre possibile osservare l'estensione verticale totale dell'habitat coralligeno, perché puó superare abbondantemente le profondità massime raggiungibili durante un'immersione subacquea. Questo è il motivo per cui parliamo di "**estensione verticale osservata dell'habitat**", ovvero le profondità minima e massima (in metri) alle quali è stato possibile osservare l'habitat durante l'immersione; tali profondità possono essere, quindi, ben differenti dalle profondità effettivamente raggiunte dall'habitat. In altre parole, è possibile stimare la profondità raggiunta dall'habitat (se si estende pochi metri al di sotto della nostra profondità d'osservazione), oppure si puó semplicemente usare la profondità massima raggiunta durante l'immersione. Un ragionamento simile puó essere applicato anche nel caso della profondità minima, qualora non sia possibile osservarla direttamente.

L'**estensione orizzontale dell'habitat** si riferisce alla superficie orizzontale occupata dall'habitat coralligeno nel sito d'osservazione. E' sufficiente stimare uno dei seguenti intervalli di distanza: **<5 m**, **5-10 m**, **10-20 m** o **>20 m**. Spuntare la casella corrispondente ell'estensione osservata.

La **continuità dell'habitat** indica se il coralligeno è limitato ad una singola chiazza isoltata (circondata, ad esempio, da sedimento o roccia nuda), se è discontinuo (molteplici chiazze separate) o se invece è continuo, ininterrotto (nel riquadrio le immagini sono ordinate da sinistra a destra, rispettivamente). Spuntare la casella corrispondente alla situazione osservata.

3. Le pressioni che agiscono sull'habitat

Tutte le pressioni, siano esse di origine umana o naturale, possono minacciare la sopravvivenza degli organismi e la stabilità dell'habitat. In alcuni casi è difficile prevenire una pressione o ridurne gli effetti negativi (è il caso, ad esempio, delle specie invasive). Tuttavia, in alcuni casi, il monitoraggio delle pressioni puó favorire la gestione e la riduzione degli effetti negativi indotti (es. controllo degli ancoraggi, limitazioni della pesca, subacquea responsabile, etc.)

Durante l'immersione, è possibile **stimare gli impatti delle pressioni** (elencate di seguito) sulla base delle proprie impressioni, quantificandole secondo le seguenti classi: **0 = assente**; + = **limitato**; ++ = **esteso.**

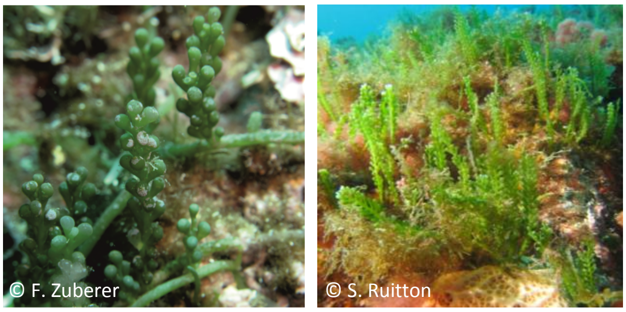

Caulerpa cylindracea

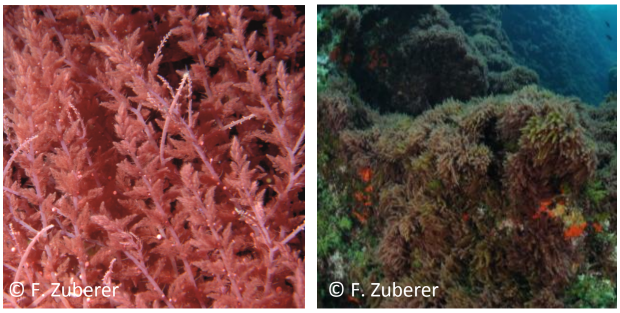

Asparagopsis spp.

L'alga verde *C. cylindracea* e le alghe del genere *Asparagopsis* hanno due caratteristiche comuni: sono **aliene**, ovvero non originarie del mar Mediterraneo (specie tropicali che sono state introdotte o sono arrivate autonomamente, e si sono poi stabilite nel nostro mare); e sono **invasive**, cioè si diffondono ovunque e risultano vincenti nella competizione con gli organismi indigeni. La biodiversità locale e la stabilità dell'habitat sono minacciate da tali specie, è perció particolrmente importante monitorarne la presenza e l'abbondanza.

Più foto: [*C. cylindracea*], [*Asparagopsis* spp].

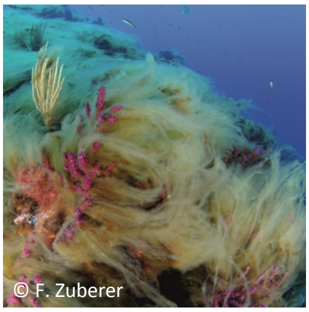

Aggregati mucillaginosi

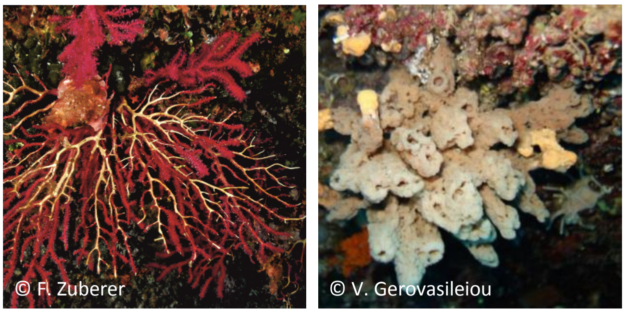

Necrosi/eventi di mortalità

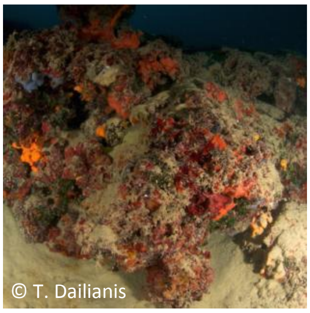

Sedimentazione

Gli **aggregati mucillaginosi** sono composti da alghe microscopiche che producono delle sostanze galatinose responsabili del caratteristico aspetto filamentoso giallasto. Tali aggregati possono svilupparsi enormemente in particolari condizioni di temperatura e circolazione delle acque, ricoprendo i fondali e soffocando letteralmente gli organismi bentonici.

La presenza di aggregati mucillaginosi, insieme alla persistenza di elevate temperature dell’acqua per lunghi periodi, possono causare la **necrosi** (morte di tessuto vivente) parziale o totale degli organismi, o addirittura **eventi di mortalità** di massa di intere popolazioni (l’insieme degli organismi appartenenti alla medesima specie).

Un’intensa **sedimentazione** puó essere causata da eventi naturali, come delle piogge intense, o da attività umane, ad esempio attività edilizie o estrattive, e lo scarico delle acque urbane in zone costiere. Qualunque sia la sua origine, il deposito eccessivo di sedimento sui substrati rocciosi causa il seppellimento degli organismi e la degradazione dell’habitat.

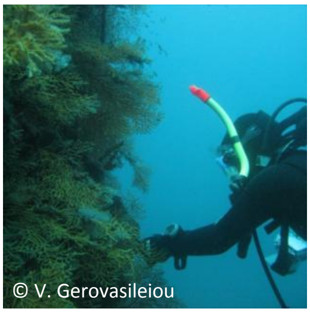

Danni causati dai subacquei

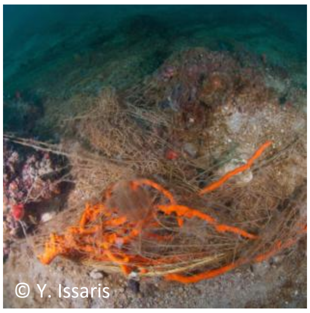

Attrezzi da pesca

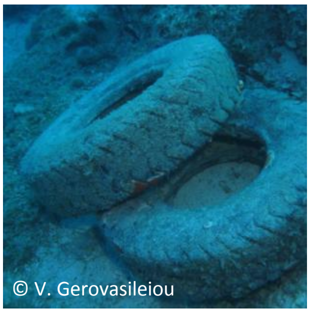

Rifiuti

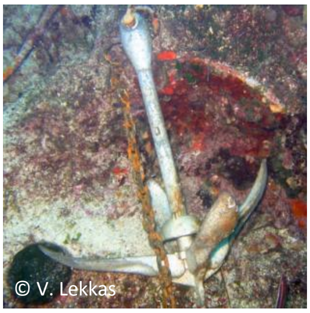

Ancore/segni d’ancoraggio

Rifiuti di differente natura, **attrezzature da pesca** abbandonate e gli **ancoraggi** possono provocare seri danni all’habitat coralligeno.

Anche **la scarsa attenzione da parte dei subacquei puó causare danni** notevoli. I subacquei possono danneggiare gli organismi urtandoli col corpo o con le pinne. Tra gli altri, i briozoi eretti calcificati sono particolarmente sensibili ai danni meccanici, e per questo sono considerati indicatori di frequentazione subacquea. Se si osserva con attenzione il fondale al piede della falesia, la presenza di pezzi rotti o di intere colonie di briozoi e gorgonie indica che il sito è frequentato da subacquei “disattenti”.

4. Le specie

La lista seguente include un numero limitato di specie, selezionate sulla base di differenti critreri: 1) il ruolo che svolgono all'interno dell'habitat (ad esempio costruttori, erosori, strutturanti del paesaggio), 2) la loro presenza regolare nell'habitat (specie tipiche del coralligeno) e 3) il loro stato di protezione (protette da leggi nazionali o internazionali). Informazioni riguardo alla loro presenza ed abbondanza (stima grossolana) ci permettono di descrivere l'habitat e di stimarne lo stato di salute.

Per la **stima dell'abbondanza** di ciascuna specie (o gruppo di specie), si considerino le seguenti classi: **0 = assente; + = scarso; ++ = abbondante; +++ = molto abbondante**. Abbiate fiducia nel vostro giudizio!

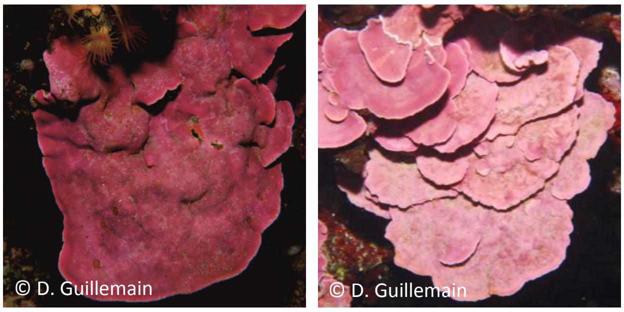

Alghe rosse calcaree

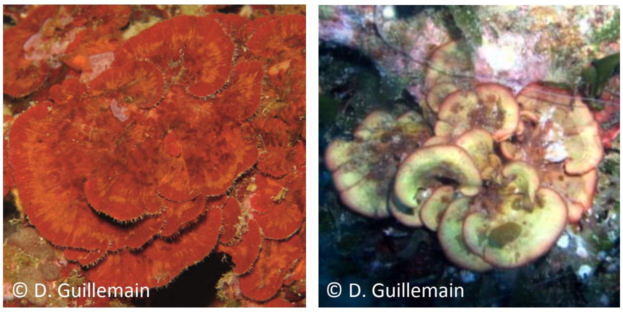

Peyssonnelia spp.

Queste alghe sono tra i maggiori rappresentanti della flora del coralligeno. Le **alghe rosse calcaree**, *Lithophyllum stictaeforme/cabiochiae* and *Mesophyllum expansum* in particolare, sono i principali costruttori del substrato calcareo. Le alghe de genere ***Peyssonnelia*** comprendono sia specie calcificate (*Peyssonnelia rosa-marina*) che non calcificate (*Peyssonnelia squamaria*).

Più foto: *L. stictaeforme/cabiochiae*, *M. expansum*, *P. rosa-marina*, *P. squamaria*.

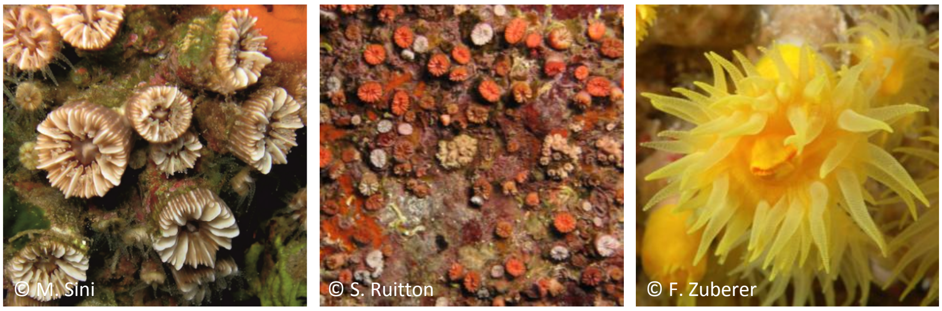

Sclerattinie

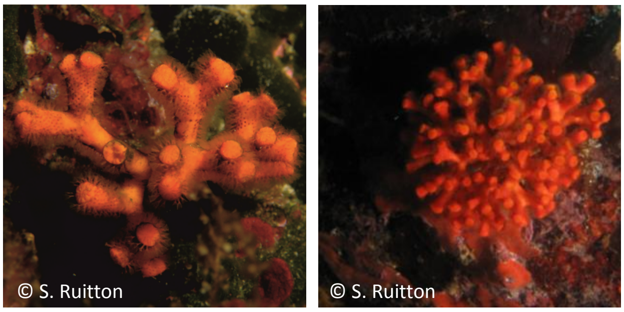

Myriapora truncata

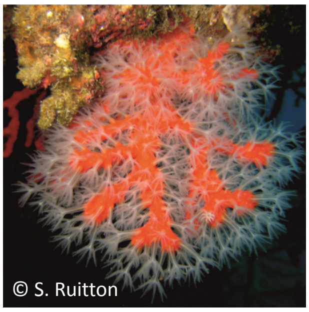

Corallium rubrum

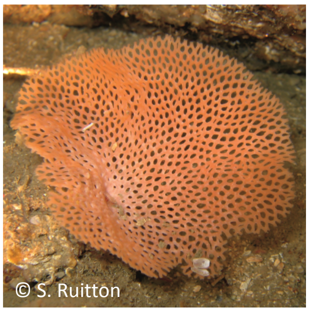

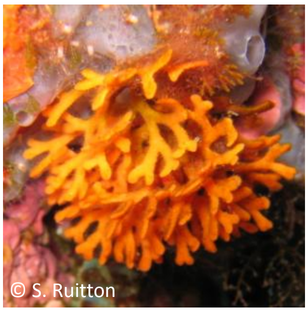

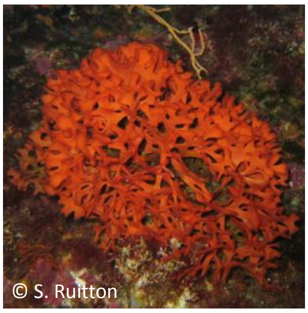

Altri briozoi

Sclerattinie e briozoi (***Myriapora truncata*** ed **altri**) producono uno scheletro calcareo, e rappresentano quindi i costruttori secondari del substrato del coralligeno. I briozoi eretti sono fragili e possono essere facilmente danneggiati in caso di impatti meccanici: questa è la ragione per cui sono considerati indicatori di frequentazione subacquea. Ad esclusione di *M. truncata*, specie particolarmente tollerante, gli altri briozoi sono sensibili all'inquinamento delle acque, e sono pertanto usati come indicatori di tale pressione.
Da non confondere con *M. truncata*, il corallo rosso, a dispetto del suo nome, non è davvero un corallo, ma la sola gorgonia che produce uno scheletro calcareo. Quindi, anch'esso rientra nella schiera dei costruttori secondari del substrato coralligeno. ***Corallium rubrum*** è stato recentemente indicato come specie in pericolo nella Lista Rossa IUCN delle Specie Minacciate.

Più foto: Sclerattinie - *Caryophyllia (Caryophyllia) smithii*, *Caryophyllia inornata*, *Hoplangia durotrix*, *Leptopsammia pruvoti*, *Madracis pharensis*, *Phyllangia mouchezii*, *Polycyathus muellerae*. *M. truncata*. *C. rubrum*. Altri briozoi - *Adeonella calveti*, *Pentapora fascialis*, *Smittina cervicornis*.

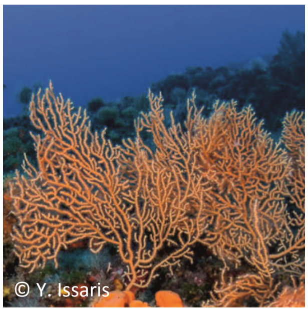

Eunicella cavolini

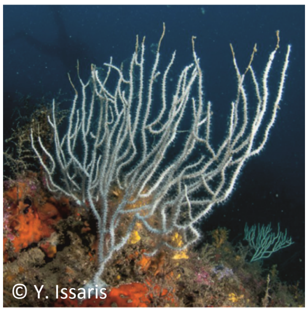

Eunicella singularis

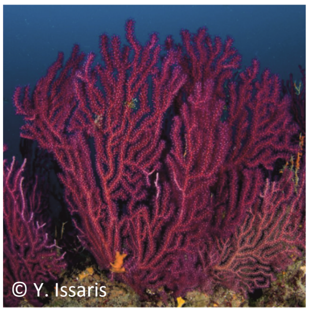

Paramuricea clavata

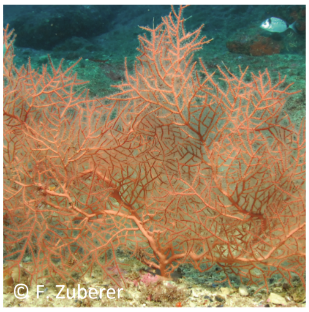

Leptogorgia sarmentosa

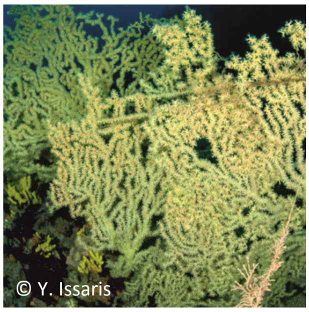

Savalia savaglia

Tra gli organismi che strutturano il paesaggio coralligeno, questi organismi arborescenti sono sicuramente i più notevoli. Le gorgonie gialle (***Eunicella cavolini***), bianche (***Eunicella singularis***) e viola (***Paramuricea clavata***) sono le più comuni. ***Leptogorgia sarmentosa***, la gorgonia arancio, preferisce vivere in acque torbide, ed è quindi considerate come indicatore di torbidità.
Savalia savaglia, il falso corallo nero, assomiglia molto ad una gorgonia ma ha in realtà relazioni di parentela più prossime con anemoni e coralli. E' una specie rara e vive generalmente in acque profonde, per questo conoscerne la presenza è importante per poter stimare la sua distribuzione.

Recentemente, *P. clavata* è stata inclusa nella Lista Rossa IUCN delle Specie in Pericolo come specie vulnerabile, ed *E. cavolini*, *E. singularis* and *S. savaglia* come specie quasi minacciate.

Più foto: *E. cavolini*, *E. singularis*, *P. clavata*, *L. sarmentosa*, *S. savaglia*.

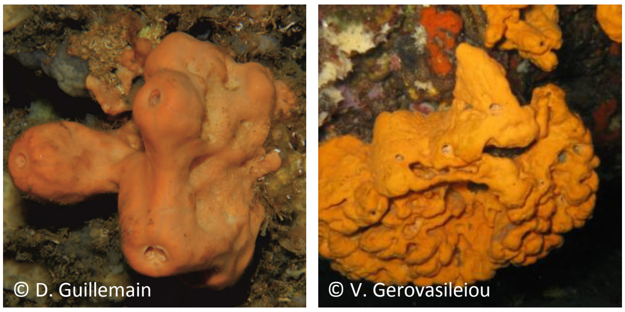

Agelas oroides

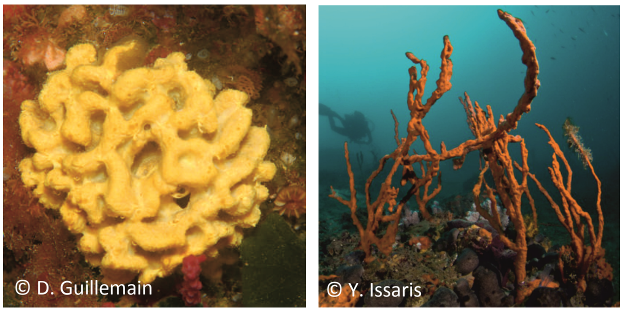

Axinella spp.

Il numero di spugne che è possibile osservare nel coralligeno è enorme. In rappresentanza di questo gruppo sono state scelte le specie più ampiamente diffuse nel mar Mediterraneo: ***Agelas oroides*** e le specie del genere ***Axinella*** (*Axinella damicornis, A. verrucosa, A. polypoides* (protetta)).

Più foto*:* *A. oroides*, *A. damicornis*, *A. verrucosa*, *A. polypoides*.

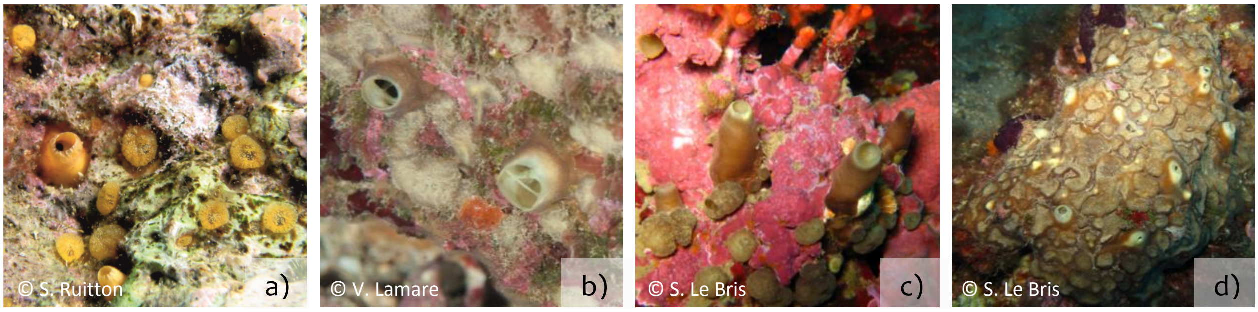

Cliona spp.

Le spugne del genere ***Cliona*** sono molto particolari, perchè sono in grado di perforare la roccia calcarea. Per questo motivo, sono i principali erosori del substrato calcareo del coralligeno. Durante i primi stadi della loro esistenza si presentano sottoforma di papille [Figure a) e b)] o piccoli "camini" [Figura c)]; successivamente crescono in forme massive [Figura d)].

Più foto: *C. celata*, *C. shmidtii*, *C. viridis*.

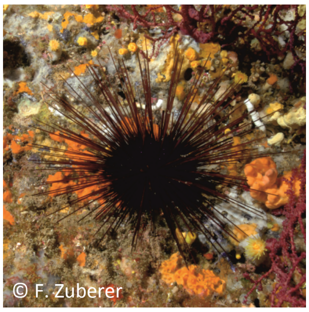

Centrostephanus longispinus

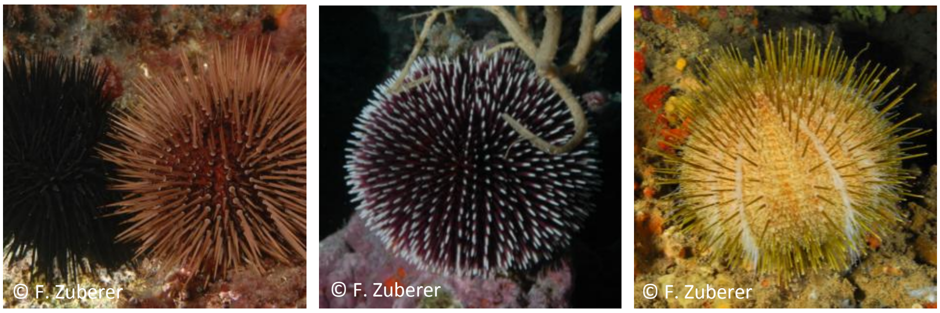

Altri ricci di mare

I ricci di mare brucano le alghe rosse calcificate, e contribuiscono quindi all'erosione del substrato calcareo. ***Centrostephanus longispinus*** (il riccio diadema) è uno dei più attivi, ed è anche una specie protetta. Anche gli **altri ricci di mare** (*Echinus melo*, *Gracilechinus acutus*, *Sphaerechinus granularis*, *Stylocidaris affinis*, *Paracentrotus lividus*, *Arbacia lixula*) contribuiscono, anche se in misura minore, all'erosione del substrato del coralligeno.

Più foto: *C. longispinus*, *E. melo*, *G. acutus*, *S. granularis*, *S. affinis*, *P. lividus*, *A. lixula*.

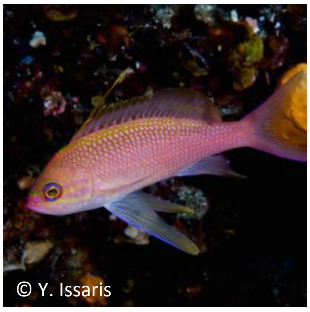

Anthias anthias

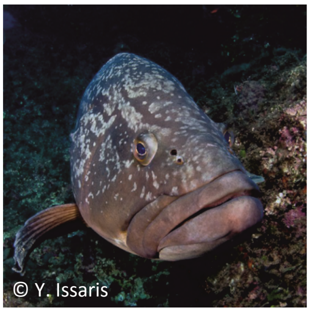

Epinephelus marginatus

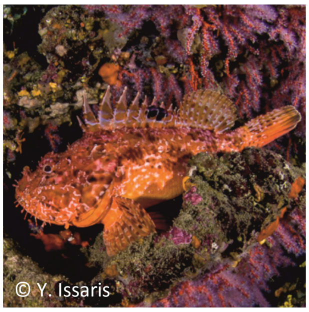

Scorpaena spp.

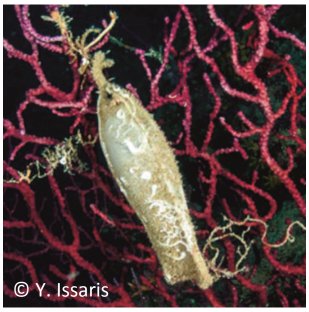

Uova di squalo

Le castagnole rosse (***Anthias anthias***), le cernie brune (***Epinephelus marginatus***) e gli scorfani (***Scorpaena* spp.**) sono pesci che abitualmente vivono nel coralligeno. Nel corso dei decenni le popolazioni di *E. marginatus* hanno subito una drammatica riduzione a causa della pesca, e sono quindi attualmente protette. Le specie del genere *Scorpaena* sono elementi importanti della pesca locale.
Squali e razze spesso depongono le loro **uova** sui rami delle gorgonie, utilizzando l'habitat coralligeno come nursery.

Più foto: *A. anthias*, *E. marginatus*, *Scorpaena maderensis*, *S. notata*, *S. porcus*, *S. scrofa*. Uova di squalo.

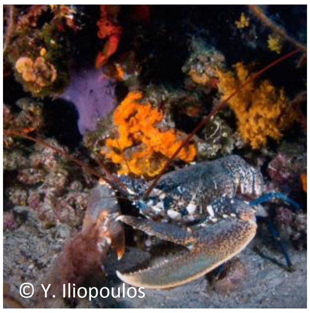

Homarus gammarus

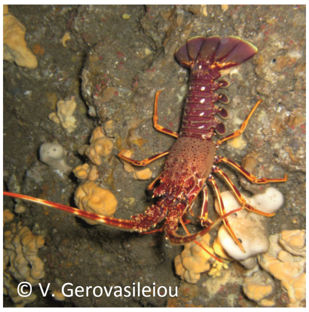

Palinurus elephas

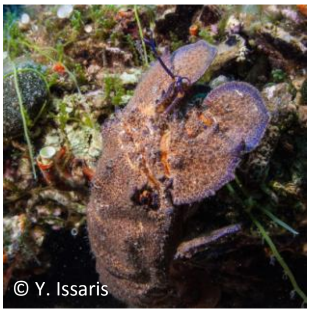

Scyllarides latus

Tra gli abitanti del coralligeno, questi crostacei sono quelli che destano il maggiore interesse commerciale, dato il loro elevato valore culinario. Per questo motivo, la raccolta di astici (***Homarus gammarus***), aragoste (***Palinurus elephas***) e magnose (***Scyllarides latus***) è regolamentata per legge.

Più foto: *H. gammarus*, *P. elephas*, *S. latus*.

E’ tempo di immergersi! Ma che cosa devo fare?

Ti abbiamo fornito una lavagnetta subacquea, su cui potrai registrare le tue osservazioni con una semplice matita. Ti consigliamo di rispettare l’ordine di riempimento da sinistra verso destra, e dall’alto verso il basso. Questo perché la lavagnetta è stata progettata seguendo alcuni accorgimenti che permettono di ottimizzare l’osservazione (e il tempo!) sott’acqua. Ad esempio, l’ordine delle specie è basato sulla loro dimensione e sulla facilità) di avvistamento: inizialmente ci sono le specie grandi e ben visibili, seguite gradualmente da specie più piccole che richiedono un’osservazione più attenta, fino ad arrivare agli organismi che vivono nascosti nelle cavità. La temperatura dell’acqua alla profondità dell’osservazione la troverai alla fine della lavagnetta: in tal modo il tuo computer subacqueo avrà tutto il tempo per rilevare il valore reale della temperatura.

Nessun campo è obbligatorio, ma raccomandiamo fortemente di non dimenticare di rilevare la profondità dell’osservazione.

MATERIALE ESSENZIALE: LAVAGNETTA, MOSCHETTONE, TORCIA, COMPUTER SUBACQUEO, BUSSOLA

Materiale opzionale: GPS, macchina fotografica

Il protocollo punto per punto:

1. Assicurati che la matita abbia la punta (un’eventuale matita di scorta puó tornare molto utile).
2. Scrivi la data e il nome del sito d’immersione (rileva le coordinate GPS, se possibile).
3. Durante la discesa, riporta la profondità alla quale hai percepito l’acqua più fredda, se l’hai incontrata.
4. Dopo aver raggiunto la profondità desiderata, individua l’area dell’osservazione: puoi scegliere una superficie limitata (di dimensione almeno pari alle tue due braccia aperte, in altezza e larghezza) oppure un piccolo percorso a profondità costante. Sentiti libero di fare quello che preferisci!
5. Segna sulla lavagnetta: profondità dell’osservazione, corrente, visibilità, estensione verticale osservata dell’habitat, estensione orizzontale, continuità dell’habitat, inclinazione, rugosità e esposizione.
6. Osserva se ci sono specie invasive o alter pressioni, e indica la classe di abbondanza per ciascuna.
7. Osserva le specie e indica la loro classe di abbondanza.
8. Segna la temperature dell’acqua alla profondità dell’osservazione.
9. A casa: carica i tuoi dati e le eventuali fotografie sul sito (http://cs.cigesmed.eu/it).
   Se il tuo computer subacqueo registra il profilo di temperature dell’immersione, puoi anche aggiungere I dati relative alla temperature dell’acqua a profondità predefinite (ogni 10 m).
   Quando avrai finito di caricare i dati, fai una foto alla lavagnetta e crea il tuo archivio. A questo punto puoi cancellare la lavagnetta e riutilizzarla ogni volt ache vorrai!

Citizen Science 4 CIGESMED

cs.cigesmed.eu/it

CIGESMED

Nome ______________________

Sito ______________________

Data

A che profondità hai incontrato l'acqua più fredda? m / mai

Profondità della osservazione:	Corrente	Visibilità
	Assente ☐ Debole ☐ Forte ☐	Acqua limpida ☐ Qualche particella ☐ Torbida ☐

Estensione dell'habitat

Verticale osservata

Prof. min:

Prof. max:

Orizzontale

<5 m ☐ 5-10 m ☐

10-20 m ☐ >20 m ☐

Continuità dell'habitat

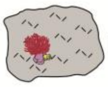 ☐ 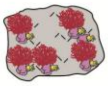 ☐ 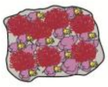 ☐

Inclinazione

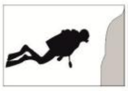 ☐ 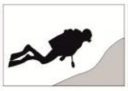 ☐ 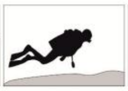 ☐ 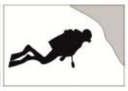 ☐

Rugosità

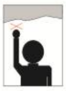 ☐ 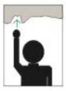 ☐ 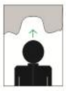 ☐ 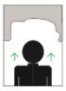 ☐

Esposizione

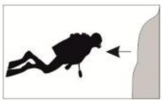

N ☐ S ☐

NE ☐ SO ☐

E ☐ O ☐

SE ☐ NO ☐

Pressioni

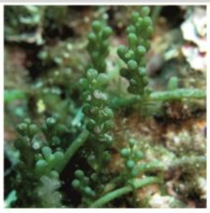

Caulerpa cylindracea

0 ☐ + ☐ ++ ☐

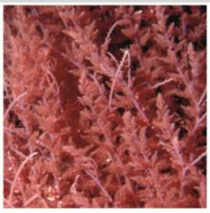

Asparagopsis spp.

0 ☐ + ☐ ++ ☐

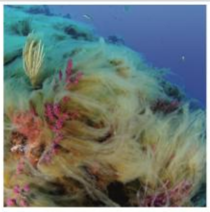

Aggregati mucillaginosi

0 ☐ + ☐ ++ ☐

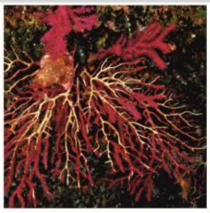

Necrosi/ eventi di mortalità

0 ☐ + ☐ ++ ☐

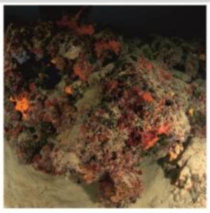

Sedimentazione

0 ☐ + ☐ ++ ☐

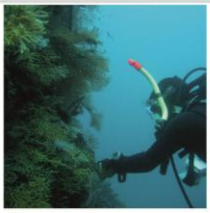

Danni causati dai subacquei

0 ☐ + ☐ ++ ☐

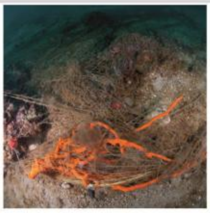

Strumenti da pesca

0 ☐ + ☐ ++ ☐

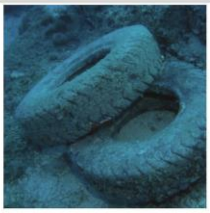

Rifiuti

0 ☐ + ☐ ++ ☐

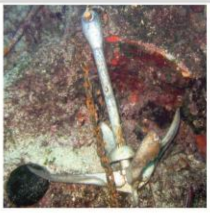

Ancore/ segni d'ancoraggio

0 ☐ + ☐ ++ ☐

0 = assente

\+ = limitato

++ = esteso

Hai osservato qualcos'altro?

Specie

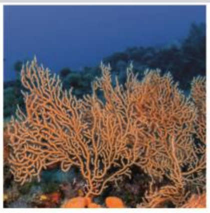
Eunicella cavolini
0 ☐ + ☐ ++ ☐ +++ ☐

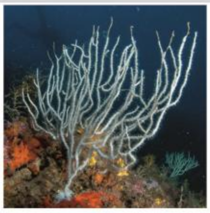
Eunicella singularis
0 ☐ + ☐ ++ ☐ +++ ☐

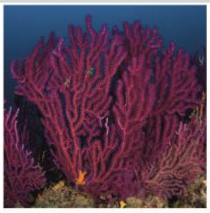
Paramuricea clavata
0 ☐ + ☐ ++ ☐ +++ ☐

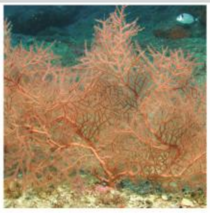
Leptogorgia sarmentosa
0 ☐ + ☐ ++ ☐ +++ ☐

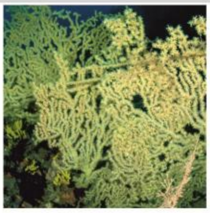
Savalia savaglia
0 ☐ + ☐ ++ ☐ +++ ☐

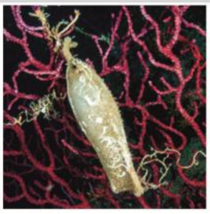
Uova di squalo
0 ☐ + ☐ ++ ☐ +++ ☐

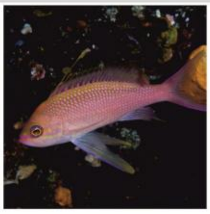
Anthias anthias
0 ☐ + ☐ ++ ☐ +++ ☐

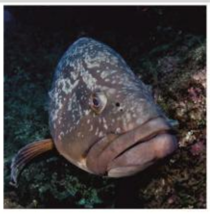
Epinephelus marginatus
0 ☐ + ☐ ++ ☐ +++ ☐

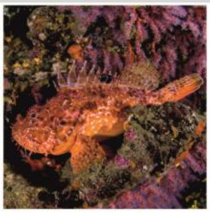
Scorpaena spp.
0 ☐ + ☐ ++ ☐ +++ ☐

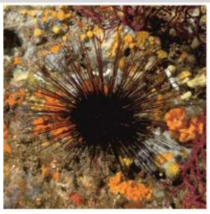
Centrostephanus longispinus
0 ☐ + ☐ ++ ☐ +++ ☐

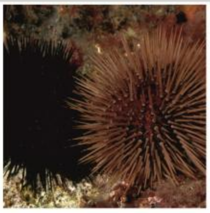
Altri ricci
0 ☐ + ☐ ++ ☐ +++ ☐

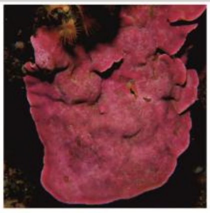
Alghe rosse calcaree
0 ☐ + ☐ ++ ☐ +++ ☐

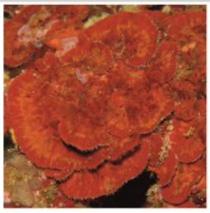
Peyssonnelia spp.
0 ☐ + ☐ ++ ☐ +++ ☐

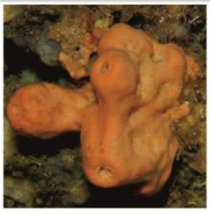
Agelas oroides
0 ☐ + ☐ ++ ☐ +++ ☐

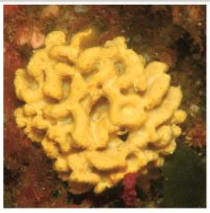
Axinella spp.
0 ☐ + ☐ ++ ☐ +++ ☐

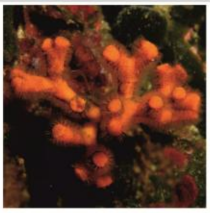
Myriapora truncata
0 ☐ + ☐ ++ ☐ +++ ☐

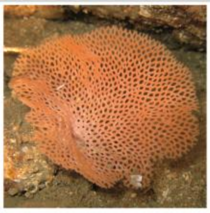
Altri briozoi
0 ☐ + ☐ ++ ☐ +++ ☐

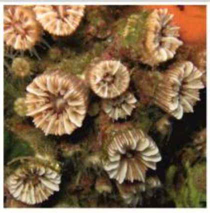
Sclerattinie
0 ☐ + ☐ ++ ☐ +++ ☐

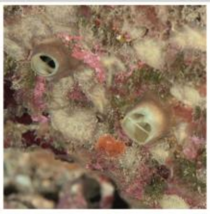
Cliona spp.
0 ☐ + ☐ ++ ☐ +++ ☐

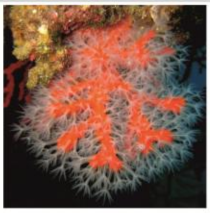
Corallium rubrum
0 ☐ + ☐ ++ ☐ +++ ☐

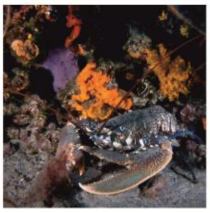
Homarus gammarus
0 ☐ + ☐ ++ ☐ +++ ☐

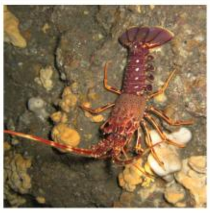
Palinurus elephas
0 ☐ + ☐ ++ ☐ +++ ☐

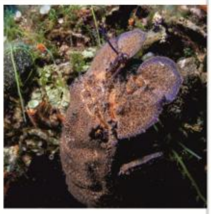
Scyllarides latus
0 ☐ + ☐ ++ ☐ +++ ☐

Temperatura dell 'acqua alla profondità dell 'osservazione:

Citizen Science 4 CIGESMED

0 = assente + = scarso ++ = abbondante +++ = molto abbondante

Per citare il presente documento: Gatti G., Thierry de Ville d'Avray L., David R., Dimitriadis C., Gerovasileiou V., Dailianis T., Sini M., Salomidi M., Dogan A., Issaris Y., Çinar M.E., Koutsoubas D., Arvanitidis C., Feral J-P. 2015. CIGESMED per i subacquei - Citizen Science per CIGESMED. SeasEra project (E.U. FP7 ERA-NET). 12 p.
