# Supplementary material for: CIGESMED for divers: Establishing a citizen science initiative for the mapping and monitoring of coralligenous assemblages in the Mediterranean Sea
Source: Biodivers Data J. 2016 Nov 1;(4):e8692. doi: 10.3897/BDJ.4.e8692 (PMC5136673; doi:10.3897/BDJ.4.e8692)
Supplement: Supplementary material 5 — Dalgıçlar için CIGESMED – CIGESMED için Vatandaş Bilimi [file biodiversity_data_journal-4-e8692-s005.pdf]

http://cs.cigesmed.eu/tr

www.cigesmed.eu

Dalgıçlar için CIGESMED – CIGESMED için Vatandaş Bilimi

“Korallijen” nedir?

Korallijen olarak adlandırılan şey, aslında, daha çok Akdeniz’e özgü olan ve loş ışıklı ortamlarda gelişen kompleks bir deniz (sualtı) habitatıdır. Korallijenli habitat, genel olarak kalkerli kırmızı algler tarafından oluşturulur ve bu algler denizsel kayalık zeminler üzerinde binlerce yıl boyunca resif benzeri yapılar oluştururlar. Bu bağlamda, korallijenli oluşumlar mercan resiflerinin Akdeniz’deki karşılıkları olarak kabul edilirler. Aynı zamanda, *Cliona* genusuna dahil süngerler veya deniz kestaneleri gibi biyo-eroderler (biyo-aşındırıcılar) olarak isimlendirilen diğer bazı organizmalar kalkerli substratları deler ve tüketirler ve bu nedenle korallijenli oluşumların yapısal karmaşıklıklarının artmasına katkıda bulunurlar. Bu kalkerli substratlar, aynı zamanda, korallijenli habitatın oluşumuna katkıda bulunan bazı uzun yaşamlı türleri de (süngerler, gorgonlar, bryozoonlar, mercanlar) desteklemiş olurlar. Sonuç olarak, çok sayıda omurgasız canlının (örneğin, nudibranşlar, krustaseler, askidianlar, ekinodermler, mollusklar) ve balıkların korallijenli habitatlarla birlik oluşturmaları, korallijenli habitatın eşsiz bir biyoçeşitlilik sıcak noktası olarak tanımlanmasına katkı sağlamaktadır.

Korallijenli habitatları neden inceliyoruz?

Dünya çapında eşsiz habitatlar olan korallijen, aletli dalışlarda gözlenecek en zengin ve en güzel deniz yapıları arasında yer almaktadır. Karmaşıklıklarına bağlı olarak bu habitatlar, bazıları ulusal ve uluslararası kanunlar tarafından korunan, ekolojik, estetik ve ticari bakımdan değerli birçok türü barındırmaktadır. Korallijenli habitatlar sıklıkla insan kaynaklı tehdit altında kalmaktadır. Yoğun demirleme, sorumsuz dalışlar, (aşırı) avcılık, çöp deşarjı, deniz yüzey suyu sıcaklık artışı (iklimsel değişimden kaynaklanan) ve yabancı tür istilaları, korallijenli habitatların ekolojik durumları üzerinde negatif etki oluşturan temel tehditler arasında yer alırlar.

Dalgıçlar için CIGESMED projesine katılarak, korallijenli habitatların ve denizel çevrenin keşfedilmesine ve korunmasına katkıda bulunabilir ve aynı zamanda denizel biyoçeşitlilik konusundaki bilgilerinizi arttırabilirsiniz.

Ne inceliyoruz?

1. <u>Dalış noktası hakkında genel bilgiler</u>

Gözlem derinliğinde su sıcaklığı:

Su sıcaklığı, tür yaşamı için temel bir parametre olup, **gözlem derinliğine** ilişkin böyle bir veriye sahip olmak önemlidir.

Daha soğuk suya hangi derinlikte rastladınız?	m / asla

Bu derinlik, su **sıcaklığının** aniden düştüğü zon olan termoklinin alt sınırına karşılık gelen derinliktir [sığ (daha sıcak) sulardan derin (daha soğuk) sulara geçiş]. Bazen bu sınır, sıcak akıntılar yüzünden beklenenden daha derinlerde bulunabilir. Sonuç olarak, genelde daha soğuk sularda yaşayan organizmalar bu gibi durumlarda daha sıcak sulara maruz kalırlar. Eğer bu olay uzun süre yaşanırsa veya kalıcı hale gelirse, özellikle gorgon ve süngerlerden birçok türün kısmi ya da genel ölümüne neden olabilir.

Lütfen, gözlem derinliğindeki su sıcaklığı ile termoklinin alt sınırını (daha soğuk suya hangi derinlikte rastladınız) metre olarak belirterek kutucukları doldurunuz veya termokline hiç rastlamadıysanız asla seçeneğini işaretleyiniz.

Gözlem derinliği:	Akıntı	Berraklık
	Yok ☐ Zayıf ☐ Kuvvetli ☐	Berrak ☐ Az miktarda askı maddesi ☐ Bulanık ☐

Derinlik, denizel türlerin dağılımlarının ve korallijenli habitatların kompozisyonlarının belirlenmesindeki temel faktördür. Lütfen gözlemin yapıldığı derinliği metre olarak doldurunuz.

Gözlemin yapıldığı derinlikte, akıntı durumu ve berraklık, dalış noktasının genel özelliklerini tamamlayacaktır.

Akıntı için, üç kategori öngörülmektedir: **yok, zayıf, güçlü.**

Berraklık ile ilgili olarak da üç sınıf vardır: **berrak su, bulanık su** ve ara bir kategori olan "**az miktarda askı maddesi**".

Lütfen, ilgili kutucuklarda, gözlemlenen akıntı hızı ve berraklıkla ile ilgili kısımları işaretleyiniz.

2. <u>Dalış noktası ve habitat özellikleri</u>

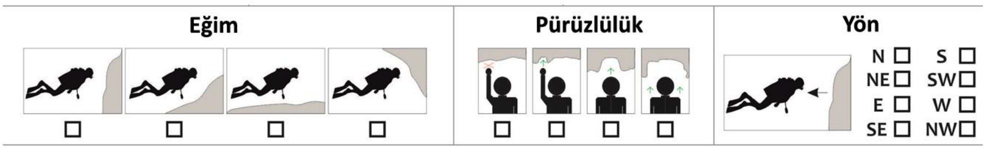

Substratumun eğimi, pürüzlülüğü ve yönü, derinlik ile beraber, korallijenli habitatların kompozisyonlarının belirlenmesindeki en temel faktörler arasındadır.

Organizmalar, üzerinde bulundukları substratumun **eğimine** göre farklı ışık yoğunluğu ve sediment birikimlerine maruz kalırlar. Substratumun eğimi ile ilgili olarak dört kategori göz önüne alınabilir: **"dikey", "eğimli", "yatay"** ve **"kaya tavanı"**.

Pürüzlülük (rugosite) kayalık zemindeki düzensizlik yani girintiler anlamına gelmektedir: substratum ne kadar düzensizse o kadar karmaşık demektir. Pürüzlülüğü sınıflandırmak için anatomik referanslara dayanan pratik bir yol izlenebilir: 1) Substratumdaki yarıklar ya da **delikler dalgıcın yumruğunun giremeyeceği kadar küçük**, 2) dalgıcın **yumruğunun gireceği kadar büyük**, 3) dalgıcın **başının gireceği kadar büyük** ve 4) dalgıcının **omuzlarının girebileceği kadar büyük**. Birçok durumda sadece bir çeşit yarık bulmak zordur. Bu durumda, küçük yarık ya da delikleri içerebileceği farz edildiği için daha büyük olan kategori seçilir.

Yön, substratın baktığı doğrultuyu ifade eder. Bunu belirlemek için, duvara yüzünüzü dönüp, duvardan gelen farazi okun gösterdiği yönün işaretlenmesi gerekir. Tahmin edileceği üzere, yatay substratların herhangi bir yön ile ifade edilmesi söz konusu olamaz.

Lütfen gözlemlediğiniz substratın eğimine, pürüzlülüğüne ve yönüne karşılık gelen kutucukları işaretleyiniz.

Habitatın boyutu			Habitat sürekliliği
Dikey	Yatay		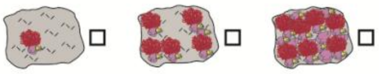
Min Derinlik:	<5 m ☐	5-10 m ☐	
Maks Derinlik:	10-20 m ☐	>20 m ☐	

Korallijenli habitatın dikey boyutunun tamamı her durumda gözlemlenmeyebilir, çünkü bu dalışınız boyunca ulaştığınız maksimum derinliği genellikle önemli ölçüde aşabilir. Bu nedenle, **“gözlemlenen habitatın dikey boyutu”** korallijenli habitatın ulaştığı maksimum derinliği değil de, korallijenli habitatın sizin gözlemlediğiniz maksimum ve minimum derinliklerini (metre olarak) ifade eder. Başka bir ifade ile, alt tarafınızda korallijenli habitatın ulaştığı derinliği tahmin edebilir ya da basitçe, dalışta ulaştığınız derinliği kullanabilirsiniz. Yukarıda bahsedilen mantık, minimum derinlik için de uygulanabilir.

Habitatın yatay boyutu, gözlem yaptığınız dalış noktasındaki korallijenli habitatın kapladığı duvarın yatay olarak boyutuna karşılık gelmektedir. Boyut aralıkları ile ilgili olarak 4 seçenek sunulmaktadır: **<5 m, 5-10 m, 10-20 m ya da >20 m.** Lütfen gözlemlenen boyutlara karşılık gelen kutucukları işaretleyiniz

Habitat sürekliliği, korallijenin tek izole parça ile kısıtlı mı kaldığını (örneğin, sediment ya da çıplak kaya ile çevrili), aralıklı mı olduğunu (sadece birkaç kaplanmamış alan) ya da sürekli mi olduğunu (dikkate değer bir aralık olmaması) tanımlar (şekiller kutucukta soldan sağa doğru sıralanmıştır). Lütfen gözlemlenen durumuna karşılık gelen kutucukları işaretleyiniz

3. Habitatı etkileyen baskılar

İnsan veya doğal kaynaklı tüm baskılar türlerin yaşamını ve habitatın dengesini tehdit edebilir. Bazı durumlarda baskıların negatif etkilerini engellemek ya da düzeltmek zordur (örneğin, istilacı türlerin oluşturduğu durum). Ancak, baskıların izlenmesi, bunların yönetimi ve oluşturdukları negatif etkilerin azaltılmasına yardımcı olabilir (örneğin, demirlemenin kontrol altına alınması, balıkçılık aktivitesinin kısıtlanması, sorumlu rekreasyonal dalış, vb…).

Dalışınız esnasında sizin algınıza dayanan bir **“baskı etkisinin tahmini”** yapılabilir. Bu bağlamda, baskılara bağlı etkileri (aşağıda sunulan) şu sınıflara göre değerlendirebilirsiniz: **0=yok, + = sınırlı, ++ = yaygın.**

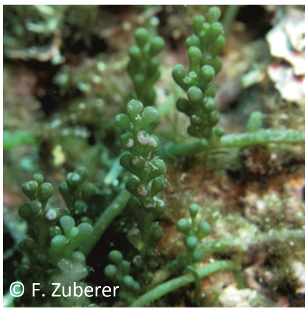

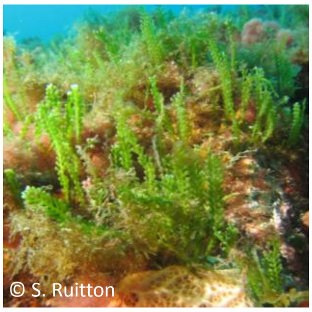

Caulerpa cylindracea

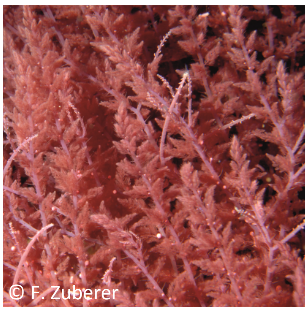

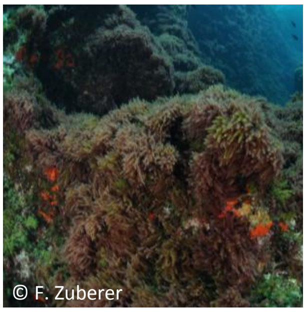

Asparagopsis spp.

Yeşil alg *Caulerpa cylindracea* ve *Asparagopsis* genusundaki alglerin iki ortak özelliği vardır: bunlar yerli olmayan türlerdir, yani bu türler Akdeniz orjinli değillerdir (Akdeniz’e taşınmış ve yerleşmiş tropikal türlerdir); ve bunlar istilacı türlerdir, yani bu türler her yere yayılabilir ve yerel türler ile girdikleri rekabeti kazanabilirler. Yerel biyoçeşitlilik ve habitat dengesi, bu gibi türler yüzünden tehlike altında olmasından dolayı bu alg türlerinin varlık ve bolluklarının (kabaca tahmin) izlenmesi kritik önem arz etmektedir.

Daha fazla fotoğraf: *C. cylindracea*, *Asparagopsis* spp.

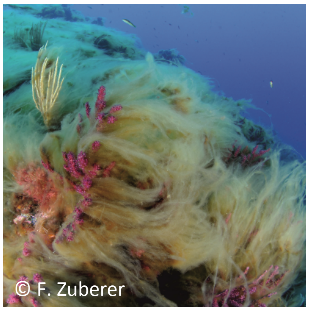

Müsilaj birikimleri

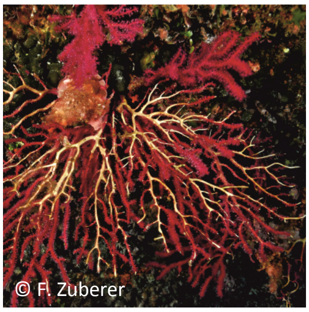

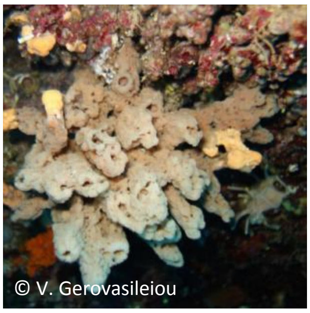

Organizmada doku ölümü/ölüm olayları

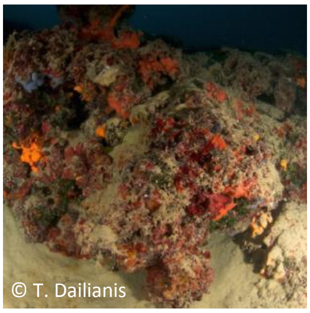

Sediment birikimi

Müsilaj birikimleri, mikroskobik algler tarafından oluşturulur ve bu türler sarımsı filament benzeri (ipliksi) görünüme sahip bazı maddeler üretirler. Sıcaklık ve su sirkülasyonu ile bağlantılı bazı özel durumlar oluştuğunda, müsilaj birikimleri aşırı biçimde artarak deniz zeminini tamamen kaplayabilir ve böylece bentik organizmaları tamamen öldürebilirler.

Müsilaj birikimleri, uzun süren sıra dışı yüksek su sıcaklığı ile birleşirse, organizmaların kısmen ya da tamamen **nekrozuna** (canlı doku ölümüne) veya bütün populasyonun kitlesel **ölüm olaylarına** neden olabilirler.

Yoğun **sedimentasyon** aşırı yağışların neden olduğu doğal şartlardan kaynaklanabildiği gibi, yapılaşma, madencilik ve kıyısal alanlara arıtılmış suların deşarjı gibi insan kaynaklı aktivitelerden de kaynaklanabilir. Kökeni ne olursa olsun, aşırı sedimentasyonun kayalık substratumlarda birikimi organizmaların gömülmesine ve habitat tahribatına yol açar.

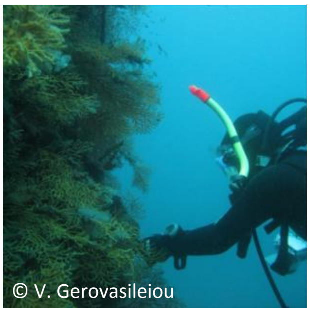

Dalgıç dikkatsizliği

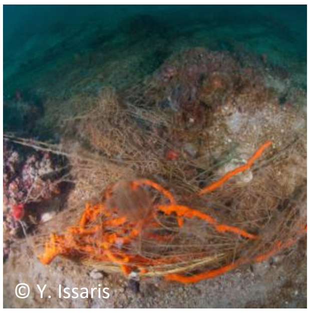

Balık ağı

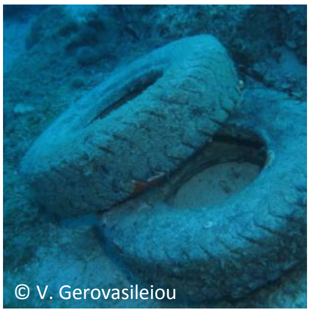

Katı atık

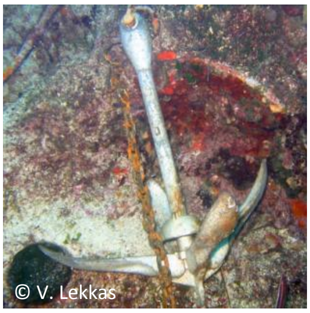

Demirleme

Farklı niteliklerde **katı atıklar**, denizde terk edilmiş **balıkçılık aletleri (balık ağı, misina vb…)** ve **demirleme**, korallijenli habitatlarda bölgesel olarak ciddi tahribatlara yol açabilirler.

Bunun yanında, **dalgıç dikkatsizliğinin korallijenli habitatlarda** negatif sonuçları olabilir. Dikkatsiz dalgıçlar vücutları veya paletleri ile dokunarak organizmalara zarar verebilirler. Diğerleri arasında, dikine büyüyen kalkerli Bryozoonlar mekanik darbelere karşı oldukça hassaslardır; bu yüzden bu türler, belli bir bölgedeki dalış sıklığı ve yoğunluğunun göstergesi olarak kabul edilirler. Duvarın alt kesimlerinde kopmuş bryozoon ya da gorgon parçalarını veya bütün olarak kopmuş kolonileri görmek buranın dikkatsiz dalgıçlar tarafından sıklıkla ziyaret edildiğini ortaya koymaktadır.

4. Türler

Aşağıdaki liste, 1) korallijenli habitatlarda oynadıkları role [örneğin, korallijenli yapıyı meydana getirenler, biyo-eroderler (biyo-aşındırıcılar)], 2) korallijenli habitatların göstergesi olarak sağladıkları faydaya ve bu habitatlarda yaygın dağılım göstermelerine (korallijenli habitatların tipik organizmaları olmaları), 3) koruma altında olma durumlarına (ulusal ya da uluslararası kanunlarla korunan) göre seçilen sınırlı sayıda türü içermektedir. Bu türlerin varlıkları ve bolluklarına (kabaca tahmin) ilişkin veriler, bu habitatların tanımlanmasına ve ekolojik kalite durumlarının değerlendirilmesine imkân sağlamaktadır.

Her bir türün (ya da tür gruplarının) **bolluğunun değerlendirilmesi** için aşağıdaki sınıflar dikkate alınmalıdır: **0 = yok; + = nadir; ++ = bol; +++ = çok bol.** Değerlendirmenize güvenin!

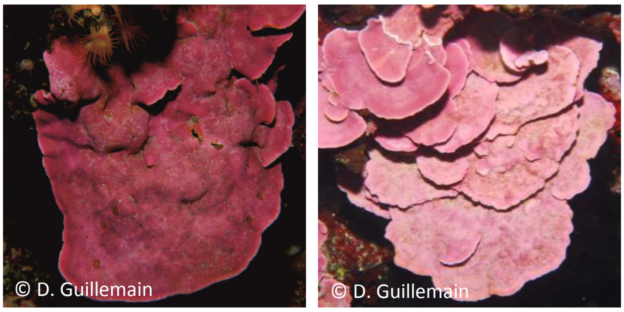

Kalkerli kırmızı algler

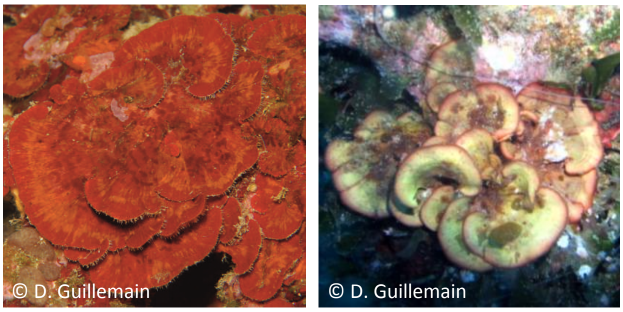

Peyssonnelia spp.

Bu kırmızı algler korallijenli floranın ana temsilcileridir. **Kalkerli kırmızı algler**, özellikle de *Lithophyllum stictaeforme/cabiochiae* ve *Mesophyllum expansum* kalkerli substratın temel oluşturucularıdırlar. ***Peyssonnelia*** genusuna ait algler, kalkerli (*Peyssonnelia rosa-marina*) ve kalkersiz (*Peyssonnelia squamaria*) türleri içerir.

Daha fazla fotoğraf: *L. stictaeforme/cabiochiae*, *M. expansum*, *P. rosa-marina*, *P. squamaria*.

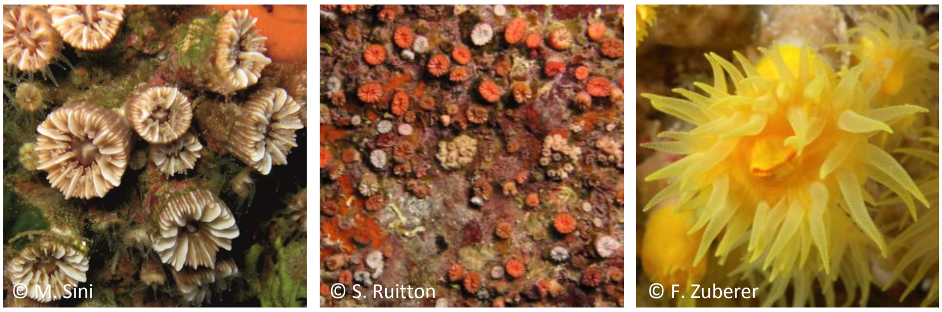

Skleraktinianlar

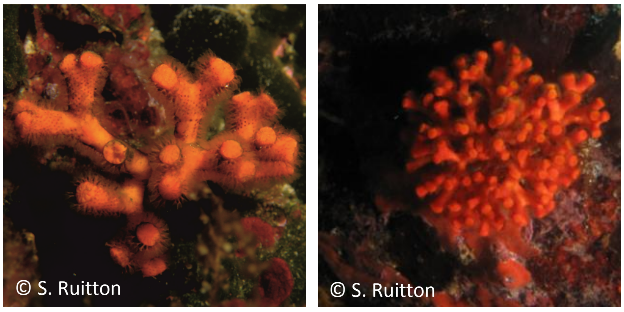

Myriapora truncata

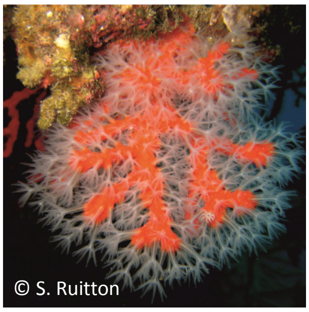

Corallium rubrum

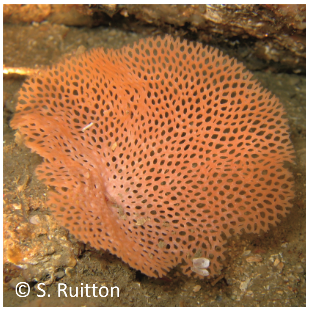

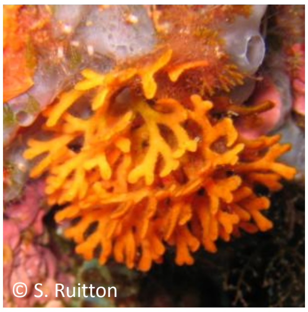

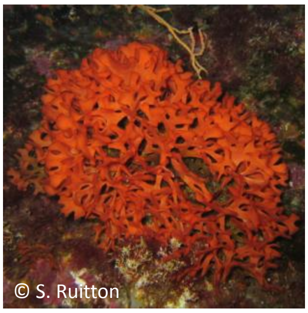

Diğer bryozoonlar

Skleraktinianlar ve bryozoonlar (***Myriapora truncata*** ve **diğerleri**), kalkerli iskelet üretirler ve bu yüzden korallijenli kalkerli substratın ikincil oluşturucularıdır. Çalı bryozoonları kırılgandır ve mekanik etki sonucu kolayca zarar görebilirler: bu durum, bu organizmaların neden belli bölgelerdeki dalış frekansının göstergeçi olduklarının sebebidir. *Myriapora truncata* hariç diğer bryozoonlar, kirlilik baskısına karşı duyarlı olmaları nedeniyle kirlilik göstergeçi olarak kullanılırlar.

Myriapora truncata ile karıştırmamak adına, kırmızı mercan ismine rağmen gerçek bir mercan değildir, ancak kalkerli iskelet üreten tek gorgondur. Bu yüzden bu tür, korallijenli substratların ikincil oluşturucusu olarak kabul edilebilir. ***Corallium rubrum*,** kısa süre önce IUCN'in tehdit altındaki ve nesli tehlike altındaki türler Kırmızı Listesine dahil edilmiştir.

Daha fazla fotoğraf: Skleraktinianlar - *Caryophyllia (Caryophyllia) smithii*, *Caryophyllia inornata*, *Hoplangia durotrix*, *Leptopsammia pruvoti*, *Madracis pharensis*, *Phyllangia mouchezii*, *Polycyathus muellerae*. Diğer bryozoonlar - *Adeonella calveti*, *Pentapora fascialis*, *Smittina cervicornis*. *M. truncata*. *C. rubrum*.

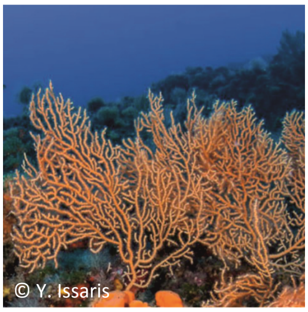

Eunicella cavolini

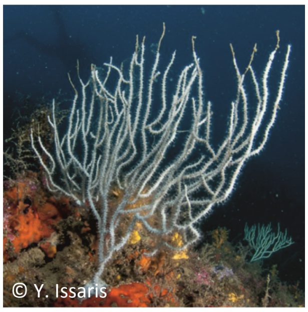

Eunicella singularis

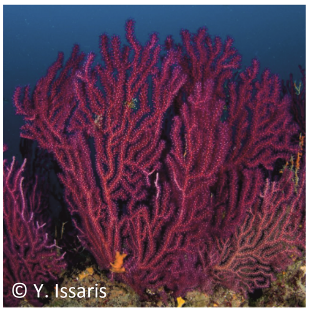

Paramuricea clavata

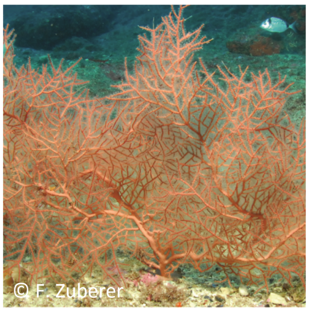

Leptogorgia sarmentosa

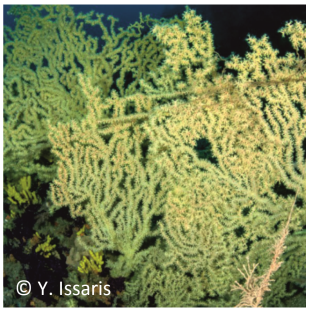

Savalia savaglia

Korallijen habitatını oluşturan organizmalar arasında, bu ağaç-şekilli türler kesinlikle en göze çarpanlardır. Sarı (***Eunicella cavolini***), beyaz (***Eunicella singularis***) ve mor/kırmızı gorgonlar (***Paramuricea clavata***) en yaygın olanlarıdır. Turuncu gorgon ***Leptogorgia sarmentosa***, bulanık suları tercih eder, dolayısıyla bu tür bulanıklığın bir göstergesi olarak kabul edilir. Çalı anemonu, ***Savalia savaglia,*** gorgon türleri gibi görünse de, bu grup organizmalara ait değildir. Esasen bu türün anemon ve mercanlarla daha yakın bir akrabalığı vardır, oysa bu tür nadir görünür ve derin sularda yaşar. Bu türün rapor edilmesi dağılımının belirlenmesi için önemlidir.

Kısa süre önce *P. clavata* hassas bir tür olarak IUCN'in tehdit altındaki türleri kapsayan, *E. cavolini*, *E. singularis* ve *S. savaglia* ise tehdide yakın türleri içeren kırmızı Listesine dahil edilmiştir.

Daha fazla fotoğraf: *E. cavolini*, *E. singularis*, *P. clavata*, *L. sarmentosa*, *S. savaglia*.

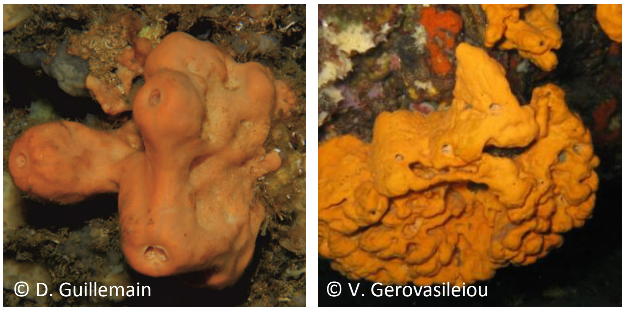

Agelas oroides

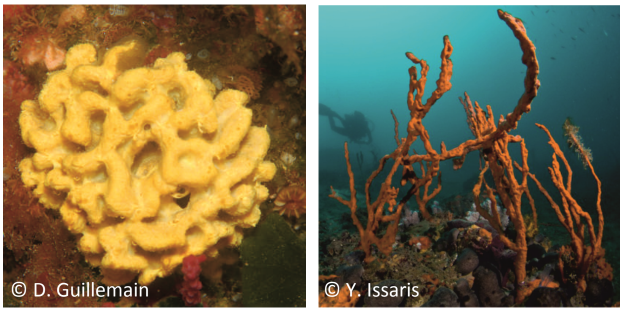

Axinella spp.

Korallijenli resiflerde gözlenebilen süngerlerin sayısı oldukça yüksektir. Bu yüzden, bu grup türlerin temsil edilmesi, Akdeniz genelinde en yaygın olanlar ile sınırlandırılmıştır: ***Agelas oroides*** ve ***Axinella*** genusuna ait türler [*Axinella damicornis, A. verrucosa, A. polypoides* (koruma altında)].

Daha fazla fotoğraf: *A. oroides*, *A. damicornis*, *A. verrucosa*, *A. polypoides*.

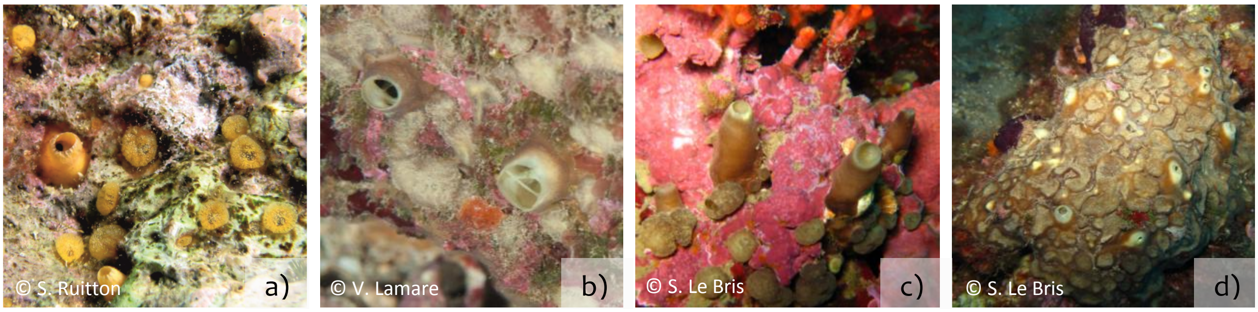

Cliona spp.

Cliona genusuna ait süngerler eşsizdir çünkü bu türler kalkerli kayaları delerler. Bu yüzden bu genusa ait türler korallijenli kalkerli substratın ana aşındırıcılarıdır. Yaşamlarının ilk safhalarında papilla formunda (Şekil a ve b) ya da küçük "baca" (Şekil c) şeklinde görünürken daha sonra öbek şeklinde büyürler (Şekil d).

Daha fazla fotoğraf: *C. celata*, *C. shmidtii*, *C. viridis*.

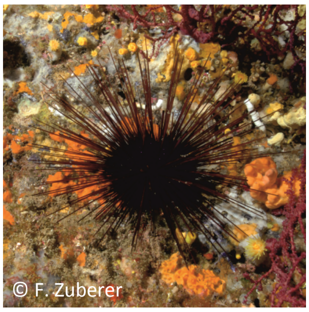

Centrostephanus longispinus

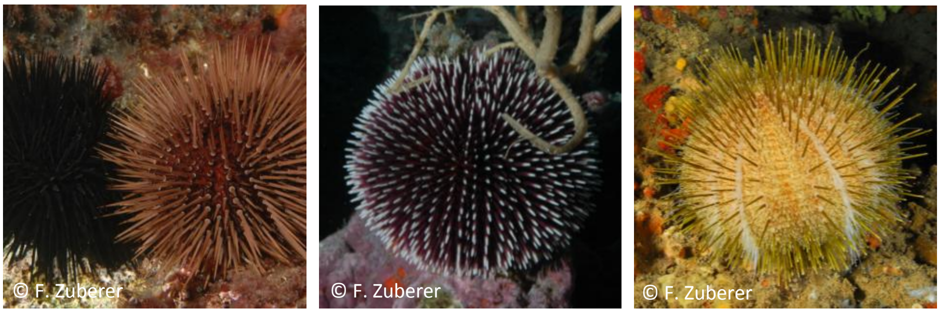

Diğer deniz kestaneleri

Denizkestaneleri kalkerli kırmızı algleri tüketirler, ve bu yüzden de kalkerli substratın erozyonuna neden olurlar. ***Centrostephanus longispinus*** (uzun dikenli denizkestanesi) en aktif tüketicilerden biri olup, aynı zamanda koruma altında olan bir türdür. **Diğer denizkestaneleri** (*Echinus melo*, *Gracilechinus acutus*, *Sphaerechinus granularis*, *Stylocidaris affinis*, *Paracentrotus lividus*, *Arbacia lixula* gibi) de korallijenli substratın erozyonunda az da olsa bir paya sahiptirler.

Daha fazla fotoğraf: *C. longispinus*, *E. melo*, *G. acutus*, *S. granularis*, *S. affinis*, *P. lividus*, *A. lixula*.

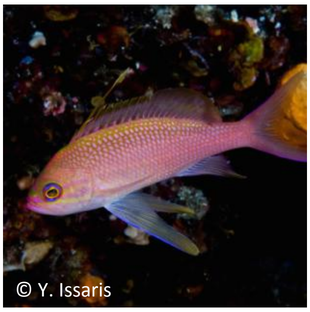	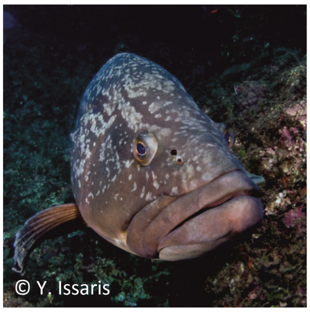	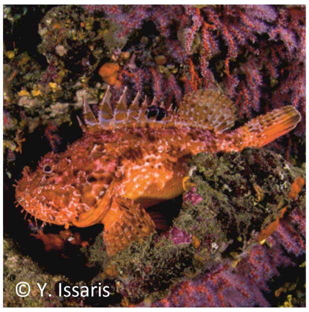	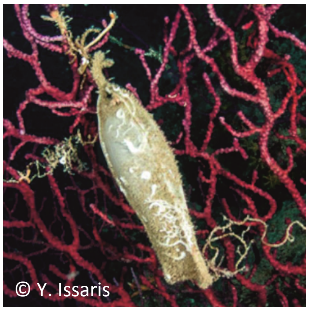
Anthias anthias	*Epinephelus marginatus*	*Scorpaena* spp.	Köpekbalığı yumurtaları

Berber balığı (***Anthias anthias***), orfoz (***Epinephelus marginatus***) ve iskorpitler (***Scorpaena* spp.**) genel olarak korallijenli habitatlarda yaşarlar. Son birkaç on yıllık zaman dilimi içerisinde *E. marginatus* populasyonları aşırı avcılığa bağlı olarak oldukça azalmış ve bu yüzden, bu tür kanunlar çerçevesinde koruma altına alınmıştır. *Scorpaena* genusuna ait türler halen bölgesel balıkçılığın önemli bileşenleridir. **Köpek balıkları** ve vatozlar korallijenli habitatları barınma yeri olarak kullanırlar ve yumurtalarını genellikle gorgonların dallarına yerleştirirler.

Daha fazla fotoğraf: *A. anthias*, *E. marginatus*, *Scorpaena maderensis*, *S. notata*, *S. porcus*, *S. scrofa*. Köpekbalığı yumurtaları.

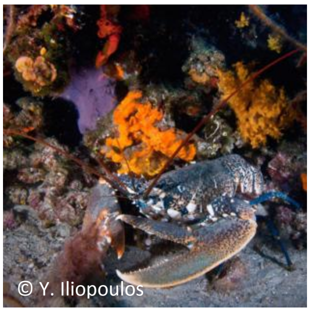	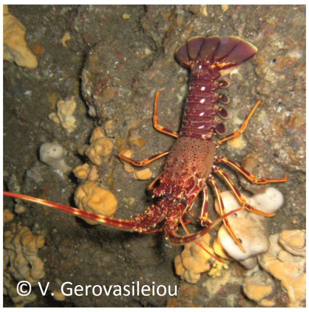	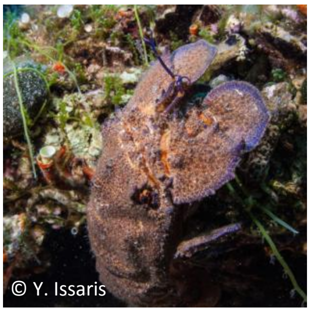
Homarus gammarus	*Palinurus elephas*	*Scyllarides latus*

Bu krustase türleri, korallijenli habitatların en yüksek ekonomik öneme sahip sakinlerindendir. Bu sebeple, Avrupa ıstakozu (***Homarus gammarus***), böcek (***Palinurus elephas***) ve büyük ayı ıstakozunun (***Scyllarides latus***) avcılığına düzenleme getirilmiştir.

Daha fazla fotoğraf: *H. gammarus*, *P. elephas*, *S. latus*.

Simdi dalış zamanı, ne yapmalıyım?

Gözlemlerinizi işaretlemeniz için bir sualtı tableti hazırlanmıştır. Basit bir kalem de gereklidir. Doldurma işlemi tabletin yukarısından aşağısına ve solundan sağına doğru yapılmalıdır. Çünkü tablet, su-altı gözleminizi pratik bir şekilde kayıt altına alabilmeniz için tasarlanmıştır. Örneğin, türlerin tabletteki sıralanışları, boyları baz alınarak yapılmıştır. Bunun kolaylığı fark edilebilir: tablet üzerine ilk önce büyük ve rahatlıkla görülebilen türler, daha sonra ise substrata daha yakından bakmayı gerektiren küçük boyutlu türler ya da oyuklarda yaşayan türler daha sonra yerleştirilmiştir. Öte yandan, gözlem derinliğindeki su sıcaklığı, dalış bilgisayarına uygun ölçüm için zaman kazandırmak adına tabletin en sonunda yer almıştır.

Tablette zorunlu olan hiçbir alan yoktur ancak gözlem derinliğini işaretlemeyi unutmamanız şiddetle tavsiye edilmektedir.

GEREKLİ EKİPMANLAR: TABLET, KARABİNA, FENER, DALIŞ BİLGİSAYARI, PUSULA

İsteğe Bağlı Ekipmanlar: GPS, Sualtı fotoğraf makinesi.

Protokolün adım adım uygulanması:

1. Kalemlerin yazıp yazmadığını kontrol ediniz (yedek bir kalem de işe yarayabilir).
2. Tarihi ve dalış noktasının adını kaydediniz (mümkünse GPS koordinatlarını da).
3. Dalış esnasında, fark ettiğinizde, daha soğuk suya rastladığınız derinliği not ediniz.
4. Tercih ettiğiniz derinliğe ulaştığınızda, gözlem yapacağınız alanı seçiniz: sınırlı bir yüzey olabilir (kollarınızı açtığınızdaki en ve boydan daha küçük olmamak şartı ile) ya da sabit bir derinlikte kısa bir rota izleyebilirsiniz. İstediğinizi yapma konusunda kendinizi özgür hissedin!
5. İşaretleyiniz: gözlem derinliği, akıntı, berraklık, gözlemlenen derinlik aralığı, yatay boyut, habitat sürekliliği, eğim, pürüzlülük ve yön.
6. İstilacı tür ya da diğer baskıların olup olmadığının araştırıp, her birinin bolluğunu/derecesini işaretleyiniz.
7. Türlerin bolluk durumlarını kaydediniz.
8. Gözlem derinliğindeki su sıcaklığını işaretleyiniz.
9. Dönüşte: verilerinizi ve muhtemel fotoğraflarınızı internet sayfasına (http://cs.cigesmed.eu) yükleyiniz. Eğer dalış bilgisayarınız su kolonuna ait su sıcaklığına ilişkin veri sağlıyorsa lütfen internet sayfasındaki özel alanları, her 10 m için kaydedilen değerleri ekleyerek doldurunuz.
   Veri girişi başarılı biçimde tamamlandığında, tableti basit bir silgi ile silerek daha sonra tekrar istediniz kadar kullanabilirsiniz.

cs.cigesmed.eu/tr	Gözlemci adı
	Bölge
	Tarih

Daha soğuk suya hangi derinlikte rastladınız? m / asla

Gözlem derinliği:	Akıntı	Berraklık
	Yok ☐ Zayıf ☐ Kuvvetli ☐	Berrak ☐ Az miktarda askı maddesi ☐ Bulanık ☐

Habitatın boyutu

Dikey
Min Derinlik:
Maks Derinlik:

Yatay
<5 m ☐ 5-10 m ☐
10-20 m ☐ >20 m ☐

Habitat sürekliliği

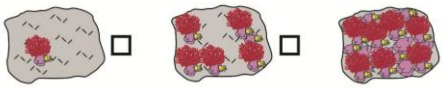
☐ ☐ ☐

Eğim

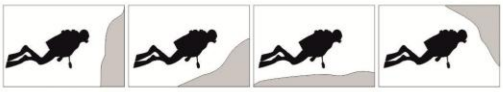
☐ ☐ ☐ ☐

Pürüzlülük

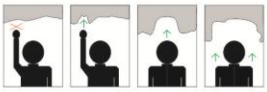
☐ ☐ ☐ ☐

Yön

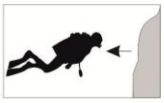

K ☐ G ☐
KD ☐ GB ☐
D ☐ B ☐
GD ☐ KB ☐

Baskılar

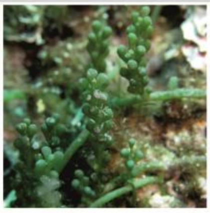
Caulerpa cylindracea
0 ☐ + ☐ ++ ☐

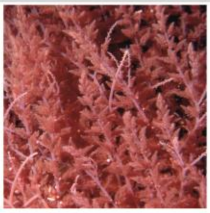
Asparagopsis spp.
0 ☐ + ☐ ++ ☐

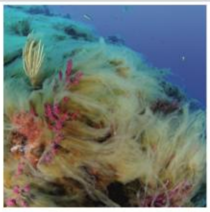
Müsilaj birikimi
0 ☐ + ☐ ++ ☐

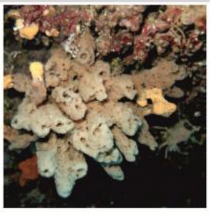
Organizmada doku ölümü / ölüm olayları
0 ☐ + ☐ ++ ☐

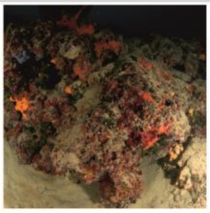
Sediment birikimi
0 ☐ + ☐ ++ ☐

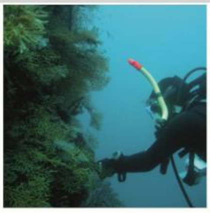
Dalgıç dikkatsizliği
0 ☐ + ☐ ++ ☐

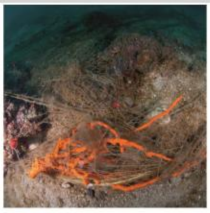
Balık ağı
0 ☐ + ☐ ++ ☐

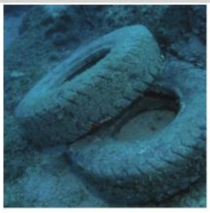
Katı atık
0 ☐ + ☐ ++ ☐

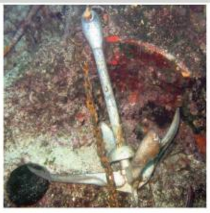
Demirleme
0 ☐ + ☐ ++ ☐

0 = Yok
\+ = Sınırlı
++ = Yaygın

Başka birşey gözlemlediniz mi?

Türler

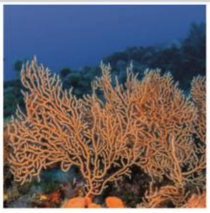
Eunicella cavolini
0 ☐ + ☐ ++ ☐ +++ ☐

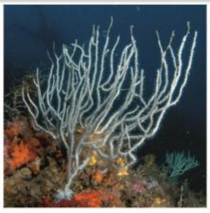
Eunicella singularis
0 ☐ + ☐ ++ ☐ +++ ☐

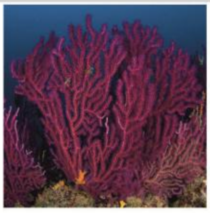
Paramuricea clavata
0 ☐ + ☐ ++ ☐ +++ ☐

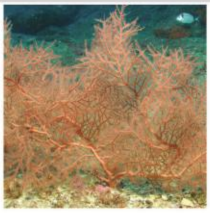
Leptogorgia sarmentosa
0 ☐ + ☐ ++ ☐ +++ ☐

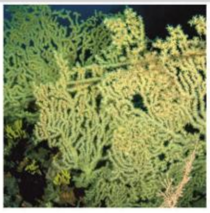
Savalia savaglia
0 ☐ + ☐ ++ ☐ +++ ☐

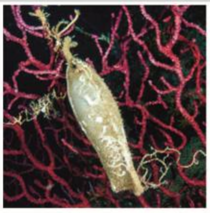
Köpekbalığı yumurtaları
0 ☐ + ☐ ++ ☐ +++ ☐

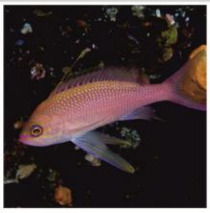
Anthias anthias
0 ☐ + ☐ ++ ☐ +++ ☐

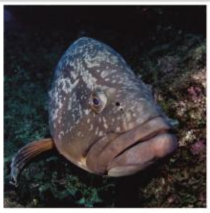
Epinephelus marginatus
0 ☐ + ☐ ++ ☐ +++ ☐

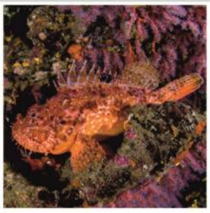
Scorpaena spp.
0 ☐ + ☐ ++ ☐ +++ ☐

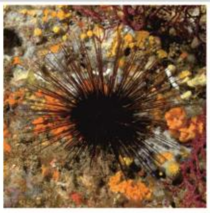
Centrostephanus longispinus
0 ☐ + ☐ ++ ☐ +++ ☐

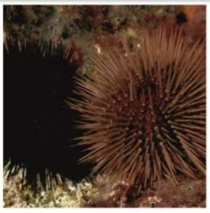
Diğer deniz kestaneleri
0 ☐ + ☐ ++ ☐ +++ ☐

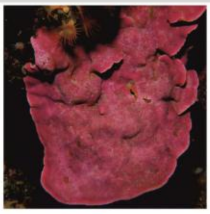
Kalkerli kırmızı algler
0 ☐ + ☐ ++ ☐ +++ ☐

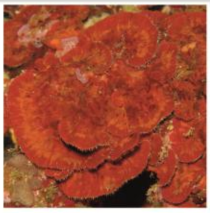
Peyssonnelia spp.
0 ☐ + ☐ ++ ☐ +++ ☐

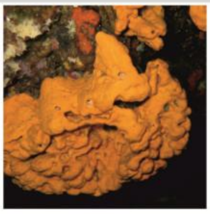
Agelas oroides
0 ☐ + ☐ ++ ☐ +++ ☐

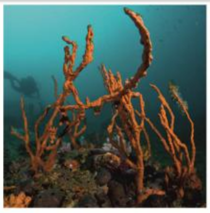
Axinella spp.
0 ☐ + ☐ ++ ☐ +++ ☐

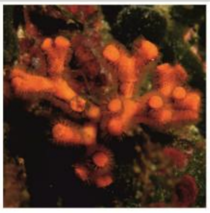
Myriapora truncata
0 ☐ + ☐ ++ ☐ +++ ☐

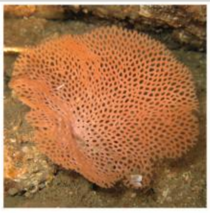
Diğer dikine büyüyen bryozoonlar
0 ☐ + ☐ ++ ☐ +++ ☐

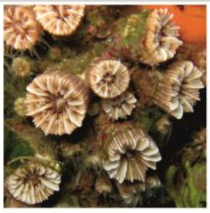
Skleraktinianlar
0 ☐ + ☐ ++ ☐ +++ ☐

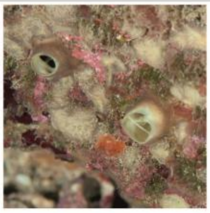
Cliona spp.
0 ☐ + ☐ ++ ☐ +++ ☐

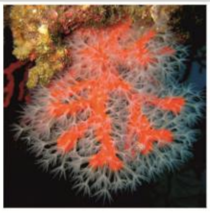
Corallium rubrum
0 ☐ + ☐ ++ ☐ +++ ☐

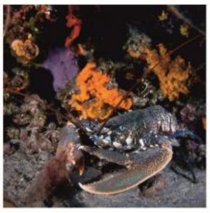
Homarus gammarus
0 ☐ + ☐ ++ ☐ +++ ☐

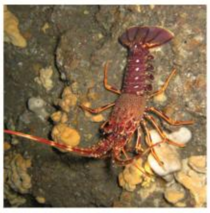
Palinurus elephas
0 ☐ + ☐ ++ ☐ +++ ☐

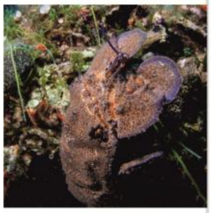
Scyllarides latus
0 ☐ + ☐ ++ ☐ +++ ☐

0 = Yok
\+ = Nadir
++ = Bol
+++ = Çok bol

Gözlem derinliğinde su sıcaklığı:

Klavuza şu şekilde atıfta bulunulmalıdır: Gatti G., Thierry de Ville d'Avray L., David R., Dimitriadis C., Gerovasileiou V., Dailianis T., Sini M., Salomidi M., Dogan A., Issaris Y., Çinar M.E., Koutsoubas D., Arvanitidis C., Feral J-P. 2015. Dalgıçlar için CIGESMED – CIGESMED için Vatandaş Bilimi. SeasEra project (E.U. FP7 ERA-NET). 12 s.
