# Supplementary material for: CIGESMED for divers: Establishing a citizen science initiative for the mapping and monitoring of coralligenous assemblages in the Mediterranean Sea
Source: Biodivers Data J. 2016 Nov 1;(4):e8692. doi: 10.3897/BDJ.4.e8692 (PMC5136673; doi:10.3897/BDJ.4.e8692)
Supplement: Supplementary material 6 — CIGESMED for divers – Citizen Science for CIGESMED [file biodiversity_data_journal-4-e8692-s006.pdf]

cs.cigesmed.eu

Name

Site

Date

At what depth you met colder water? m / never

Observation depth:	Current	Visibility
	None ☐ Weak ☐ Strong ☐	Clear water ☐ Some particles ☐ Turbid ☐

Habitat extent

Verticale observed

Min depth:

Max depth:

Horizontal

<5 m ☐ 5-10 m ☐

10-20 m ☐ >20 m ☐

Habitat continuity

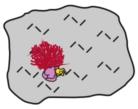 ☐ 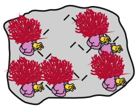 ☐ 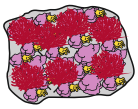 ☐

Slope

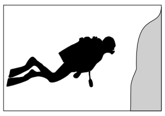 ☐ 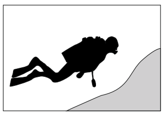 ☐ 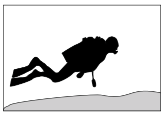 ☐ 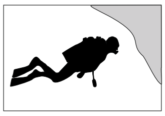 ☐

Rugosity

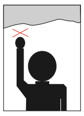 ☐ 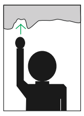 ☐ 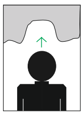 ☐ 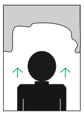 ☐

Orientation

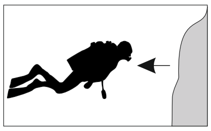

N ☐ S ☐

NE ☐ SW ☐

E ☐ W ☐

SE ☐ NW ☐

Pressures

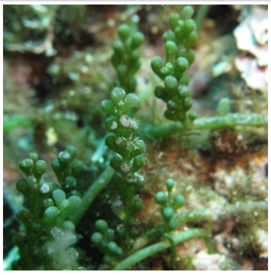

Caulerpa cylindracea

0 ☐ + ☐ ++ ☐

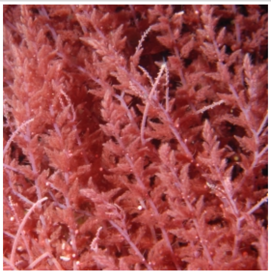

Asparagopsis spp.

0 ☐ + ☐ ++ ☐

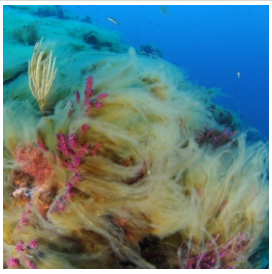

Mucilaginous aggregates

0 ☐ + ☐ ++ ☐

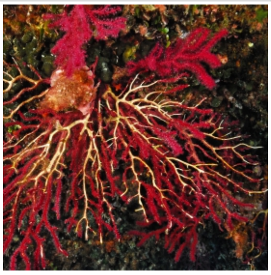

Necrosis/ mortality events

0 ☐ + ☐ ++ ☐

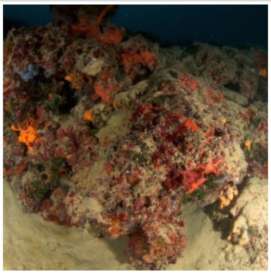

Sedimentation

0 ☐ + ☐ ++ ☐

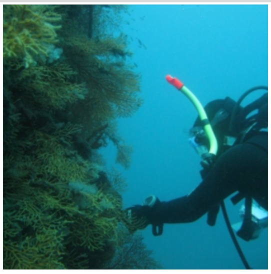

Divers recklessness marks

0 ☐ + ☐ ++ ☐

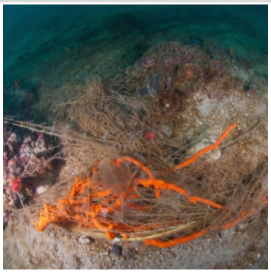

Fishing gears

0 ☐ + ☐ ++ ☐

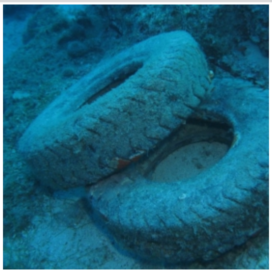

Litter

0 ☐ + ☐ ++ ☐

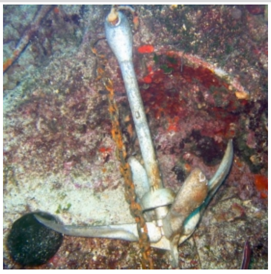

Anchoring

0 ☐ + ☐ ++ ☐

0 = absent

+ = limited

++ = extended

Did you observe anything else?

Species

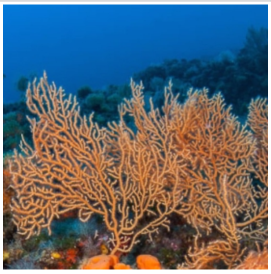

Eunicella cavolini

0 ☐ + ☐ ++ ☐ +++ ☐

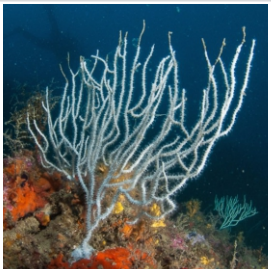

Eunicella singularis

0 ☐ + ☐ ++ ☐ +++ ☐

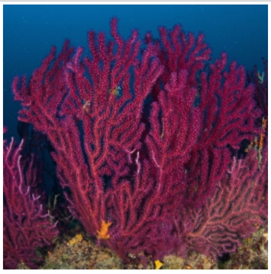

Paramuricea clavata

0 ☐ + ☐ ++ ☐ +++ ☐

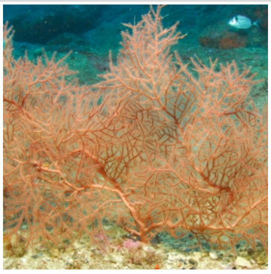

Leptogorgia sarmentosa

0 ☐ + ☐ ++ ☐ +++ ☐

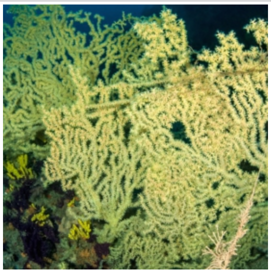

Savalia savaglia

0 ☐ + ☐ ++ ☐ +++ ☐

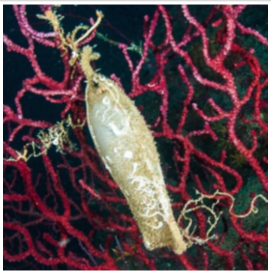

Shark eggs

0 ☐ + ☐ ++ ☐ +++ ☐

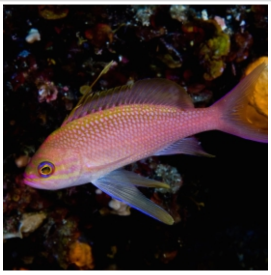

Anthias anthias

0 ☐ + ☐ ++ ☐ +++ ☐

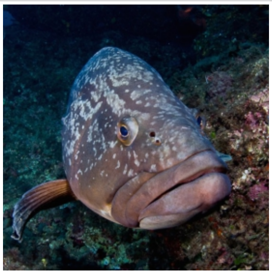

Epinephelus marginatus

0 ☐ + ☐ ++ ☐ +++ ☐

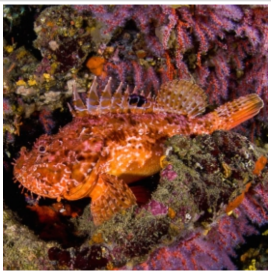

Scorpaena spp.

0 ☐ + ☐ ++ ☐ +++ ☐

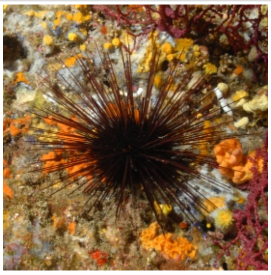

Centrostephanus longispinus

0 ☐ + ☐ ++ ☐ +++ ☐

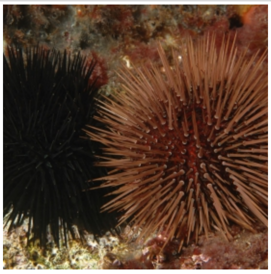

Other sea urchins

0 ☐ + ☐ ++ ☐ +++ ☐

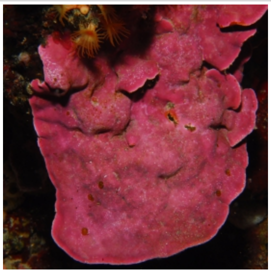

Calcareous red algae

0 ☐ + ☐ ++ ☐ +++ ☐

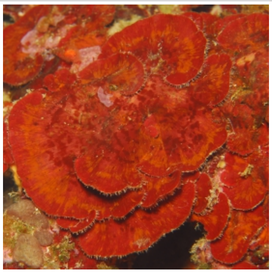

Peyssonnelia spp.

0 ☐ + ☐ ++ ☐ +++ ☐

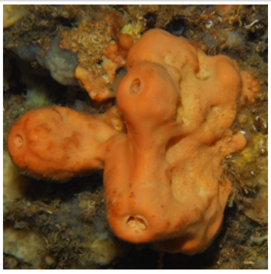

Agelas oroides

0 ☐ + ☐ ++ ☐ +++ ☐

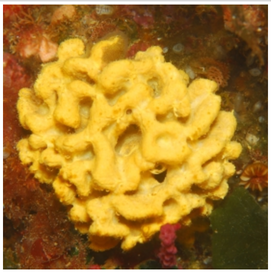

Axinella spp.

0 ☐ + ☐ ++ ☐ +++ ☐

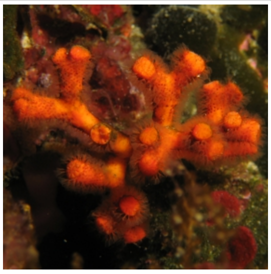

Myriapora truncata

0 ☐ + ☐ ++ ☐ +++ ☐

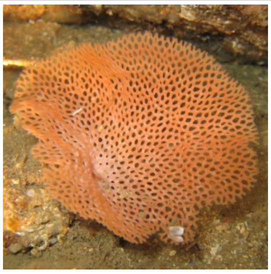

Other bryozoans

0 ☐ + ☐ ++ ☐ +++ ☐

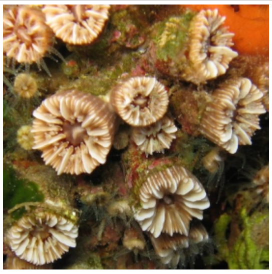

Scleractinians

0 ☐ + ☐ ++ ☐ +++ ☐

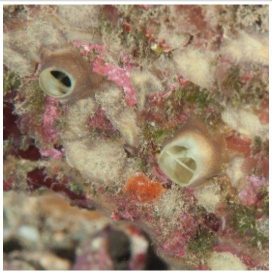

Cliona spp.

0 ☐ + ☐ ++ ☐ +++ ☐

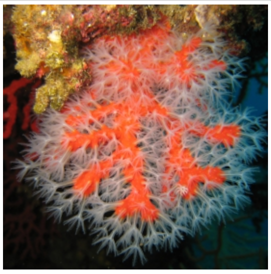

Corallium rubrum

0 ☐ + ☐ ++ ☐ +++ ☐

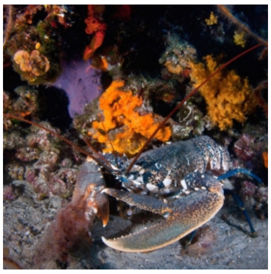

Homarus gammarus

0 ☐ + ☐ ++ ☐ +++ ☐

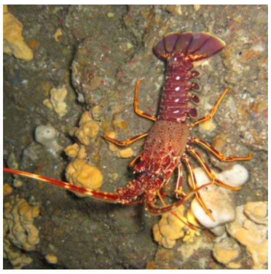

Palinurus elephas

0 ☐ + ☐ ++ ☐ +++ ☐

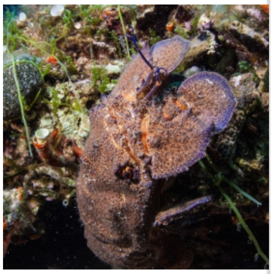

Scyllarides latus

0 ☐ + ☐ ++ ☐ +++ ☐

Water temperature at observation depth:

Citizen Science 4 GESMED

0 = absent + = scarce ++ = abundant +++ = very abundant
