# Supplementary material for: CIGESMED for divers: Establishing a citizen science initiative for the mapping and monitoring of coralligenous assemblages in the Mediterranean Sea
Source: Biodivers Data J. 2016 Nov 1;(4):e8692. doi: 10.3897/BDJ.4.e8692 (PMC5136673; doi:10.3897/BDJ.4.e8692)
Supplement: Supplementary material 7 — CIGESMED pour les plongeurs – Les sciences participatives pour CIGESMED [file biodiversity_data_journal-4-e8692-s007.pdf]

Nom

Site

Date

cs.cigesmed.eu/fr

A partir de quelle profondeur avez-vous rencontré l 'eau la plus froide ? m / jamais

Profondeur de l 'observation :	Courant	Visibilité
	Aucun ☐ Faible ☐ Fort ☐	Eau Claire ☐ Quelques particules ☐ Trouble ☐

Etendue de l 'habitat

Verticale observée

Prof. mini :

Prof. max :

Horizontale

<5 m ☐ 5-10 m ☐

10-20 m ☐ >20 m ☐

Continuité de l 'habitat

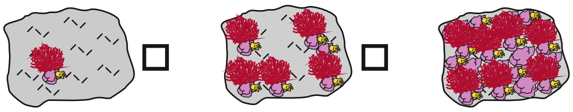

☐ ☐ ☐

Pente

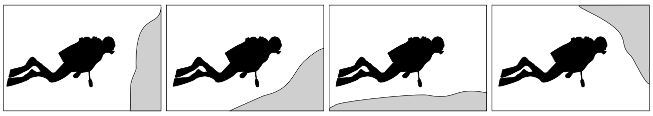

☐ ☐ ☐ ☐

Rugosité

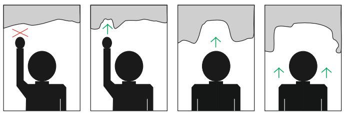

☐ ☐ ☐ ☐

Orientation

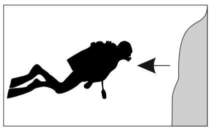

N ☐ S ☐

NE ☐ SO ☐

E ☐ O ☐

SE ☐ NO ☐

Pressions

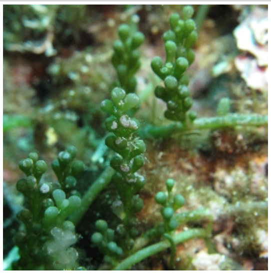

Caulerpa cylindracea

0 ☐ + ☐ ++ ☐

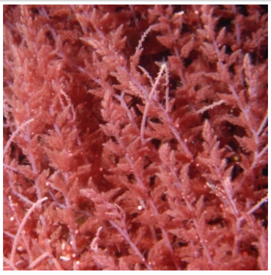

Asparagopsis spp.

0 ☐ + ☐ ++ ☐

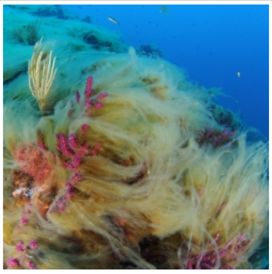

Présence de mucilage

0 ☐ + ☐ ++ ☐

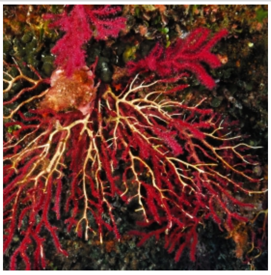

Nécrose / mortalité

0 ☐ + ☐ ++ ☐

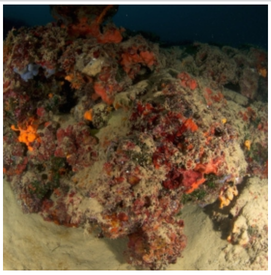

Sédimentation

0 ☐ + ☐ ++ ☐

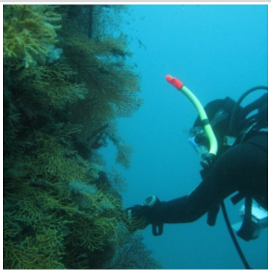

Dégâts imputables aux plongeurs

0 ☐ + ☐ ++ ☐

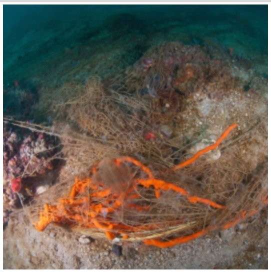

Engins de pêche

0 ☐ + ☐ ++ ☐

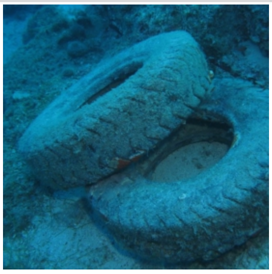

Déchets

0 ☐ + ☐ ++ ☐

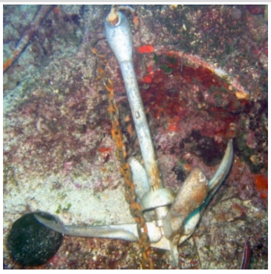

Marques d'ancrage/ ancres

0 ☐ + ☐ ++ ☐

0 = absent

+ = limité

++ = étendu

Avez-vous observé autre chose ?

Espèces

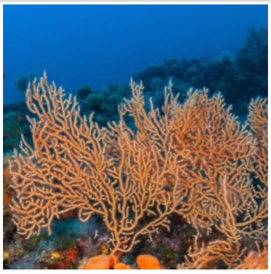

Eunicella cavolini

0 ☐ + ☐ ++ ☐ +++ ☐

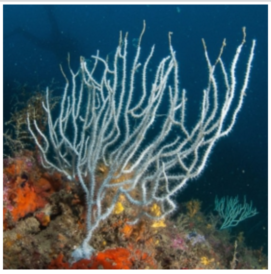

Eunicella singularis

0 ☐ + ☐ ++ ☐ +++ ☐

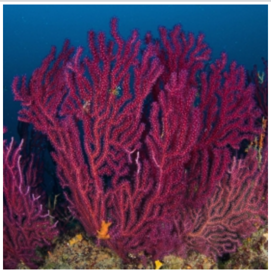

Paramuricea clavata

0 ☐ + ☐ ++ ☐ +++ ☐

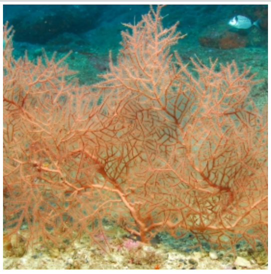

Leptogorgia sarmentosa

0 ☐ + ☐ ++ ☐ +++ ☐

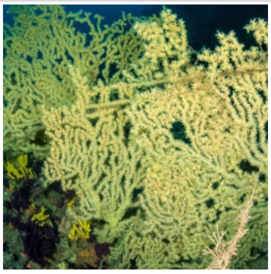

Savalia savaglia

0 ☐ + ☐ ++ ☐ +++ ☐

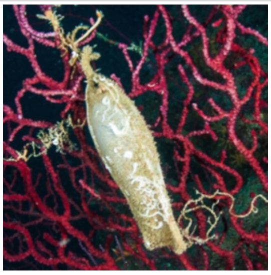

Œufs de sélaciens

0 ☐ + ☐ ++ ☐ +++ ☐

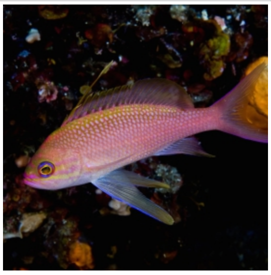

Anthias anthias

0 ☐ + ☐ ++ ☐ +++ ☐

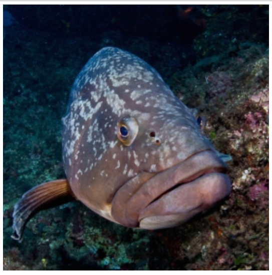

Epinephelus marginatus

0 ☐ + ☐ ++ ☐ +++ ☐

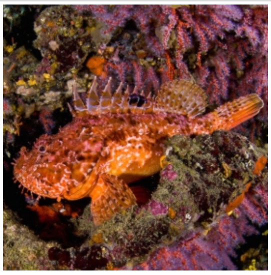

Scorpaena spp.

0 ☐ + ☐ ++ ☐ +++ ☐

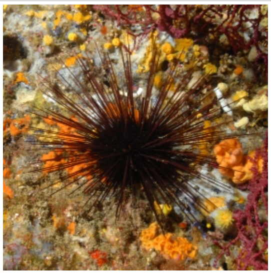

Centrostephanus longispinus

0 ☐ + ☐ ++ ☐ +++ ☐

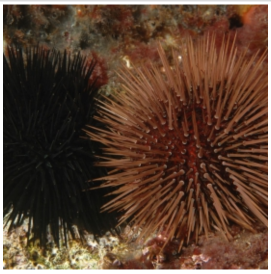

Autres oursins

0 ☐ + ☐ ++ ☐ +++ ☐

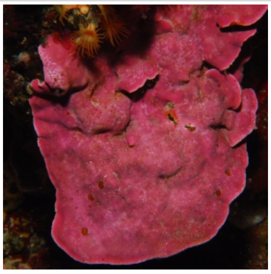

Algues rouges calcaires

0 ☐ + ☐ ++ ☐ +++ ☐

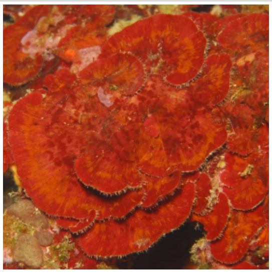

Peyssonnelia spp.

0 ☐ + ☐ ++ ☐ +++ ☐

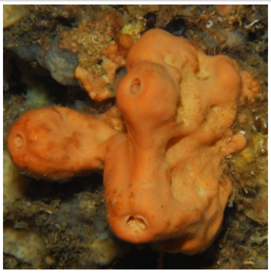

Agelas oroides

0 ☐ + ☐ ++ ☐ +++ ☐

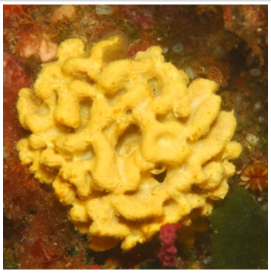

Axinella spp.

0 ☐ + ☐ ++ ☐ +++ ☐

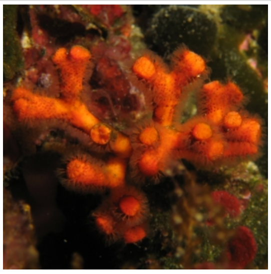

Myriapora truncata

0 ☐ + ☐ ++ ☐ +++ ☐

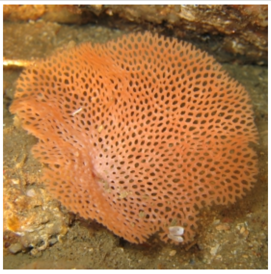

Autres bryozoaires

0 ☐ + ☐ ++ ☐ +++ ☐

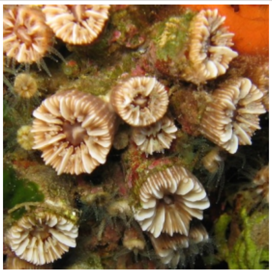

Scléractiniaires

0 ☐ + ☐ ++ ☐ +++ ☐

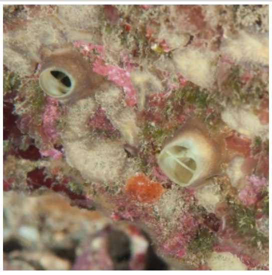

Cliona spp.

0 ☐ + ☐ ++ ☐ +++ ☐

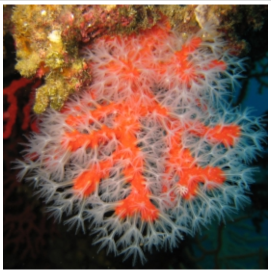

Corallium rubrum

0 ☐ + ☐ ++ ☐ +++ ☐

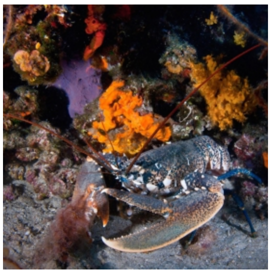

Homarus gammarus

0 ☐ + ☐ ++ ☐ +++ ☐

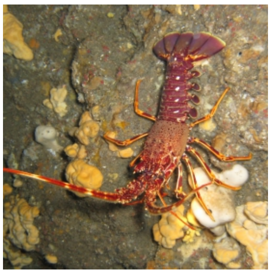

Palinurus elephas

0 ☐ + ☐ ++ ☐ +++ ☐

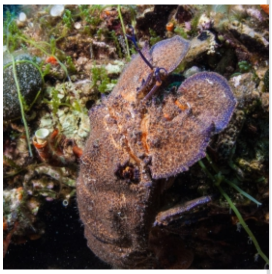

Scyllarides latus

0 ☐ + ☐ ++ ☐ +++ ☐

Température de l'eau à la profondeur de l'observation :

Citizen Science 4 GESMED

0 = absent + = peu abondant ++ = abondant +++ = très abondant
