# Supplementary material for: CIGESMED for divers: Establishing a citizen science initiative for the mapping and monitoring of coralligenous assemblages in the Mediterranean Sea
Source: Biodivers Data J. 2016 Nov 1;(4):e8692. doi: 10.3897/BDJ.4.e8692 (PMC5136673; doi:10.3897/BDJ.4.e8692)
Supplement: Supplementary material 8 — CIGESMED για δύτες, πολίτες-επιστήμονες για το πρόγραμμα παρακολούθησης των κοραλλιγενών οικοτόπων [file biodiversity_data_journal-4-e8692-s008.pdf]

Όνομα

Τοποθεσία

Ημερομηνία

Σε ποιο βάθος αισθανθήκατε σημαντική πτώση της θερμοκρασίας? m / ποτέ

Βάθος παρατήρησης:	Ένταση του ρεύματος	Ορατότητα
	Απουσία ☐ Ήπιο ☐ Δυνατό ☐	Διαυγές νερό ☐ Μερικά σωματίδια ☐ Θολά ☐

Εξάπλωση κοραλλιγενών κοινοτήτων

Κατακόρυφη εξάπλωση	Οριζόντια εξάπλωση	
Ελάχιστο βάθος	<5 m ☐	5-10 m ☐
Μέγιστο βάθος:	10-20 m ☐	>20 m ☐

Συνέχεια κοινότητας

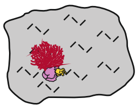 ☐ 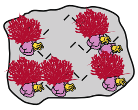 ☐ 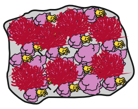 ☐

Κλίση υποστρώματος

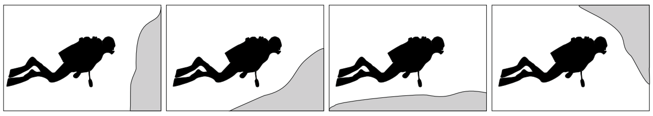

☐ ☐ ☐ ☐

Τραχύτητα

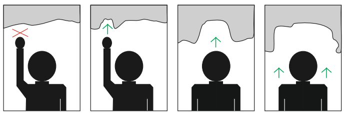

☐ ☐ ☐ ☐

Προσανατολισμός

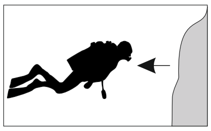

Β ☐ Ν ☐
ΒΑ ☐ ΝΔ ☐
Α ☐ Δ ☐
ΝΑ ☐ ΒΔ ☐

Πιέσεις και απειλές

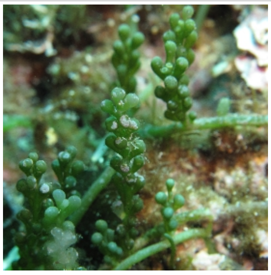

Caulerpa cylindracea

0 ☐ + ☐ ++ ☐

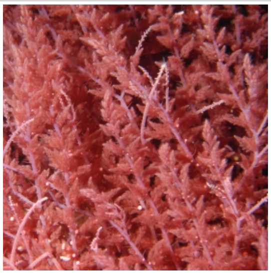

Asparagopsis spp.

0 ☐ + ☐ ++ ☐

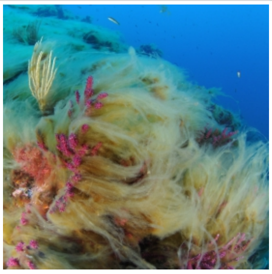

Ζελατινώδεις συναθροίσεις

0 ☐ + ☐ ++ ☐

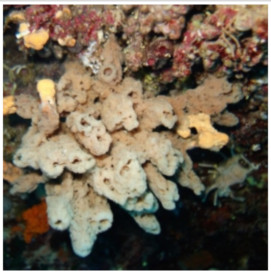

Νέκρωση/περιστατικά μαζικής θανάτωσης

0 ☐ + ☐ ++ ☐

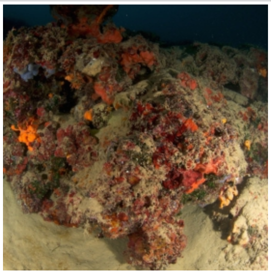

Απόθεση ιζήματος

0 ☐ + ☐ ++ ☐

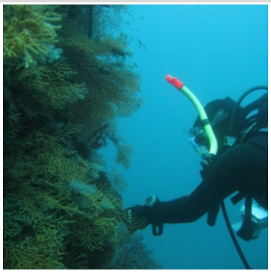

Απροσεξία δυτών

0 ☐ + ☐ ++ ☐

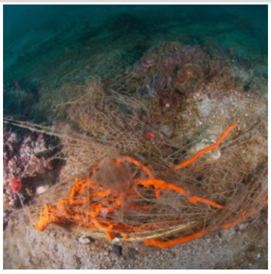

Αλιευτικά εργαλεία

0 ☐ + ☐ ++ ☐

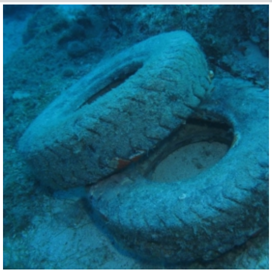

Απορρίμματα

0 ☐ + ☐ ++ ☐

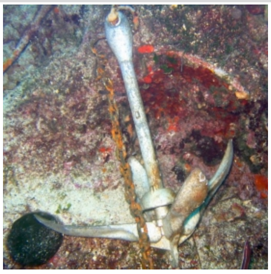

Αγκυροβολία

0 ☐ + ☐ ++ ☐

0 = Απούσα
+ = Σπάνια
++ = Άφθονη

Άλλες παρατηρήσεις

Οργανισμοί κοραλλιγενών κοινοτήτων

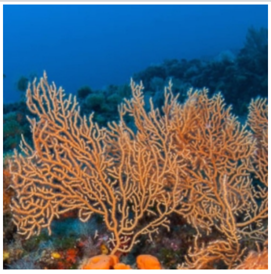

Eunicella cavolini

0 ☐ + ☐ ++ ☐ +++ ☐

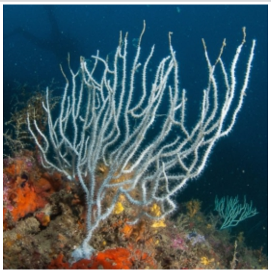

Eunicella singularis

0 ☐ + ☐ ++ ☐ +++ ☐

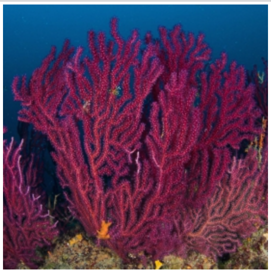

Paramuricea clavata

0 ☐ + ☐ ++ ☐ +++ ☐

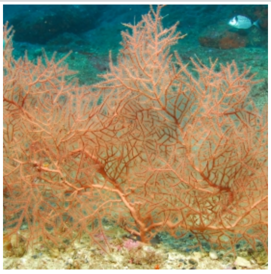

Leptogorgia sarmentosa

0 ☐ + ☐ ++ ☐ +++ ☐

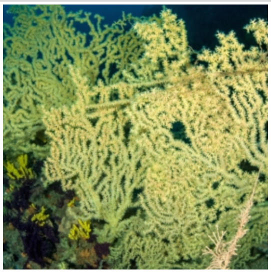

Savalia savaglia

0 ☐ + ☐ ++ ☐ +++ ☐

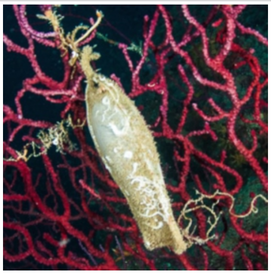

Αυγά σκυλόψαρων

0 ☐ + ☐ ++ ☐ +++ ☐

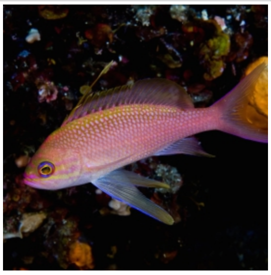

Anthias anthias

0 ☐ + ☐ ++ ☐ +++ ☐

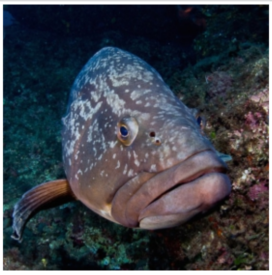

Epinephelus marginatus

0 ☐ + ☐ ++ ☐ +++ ☐

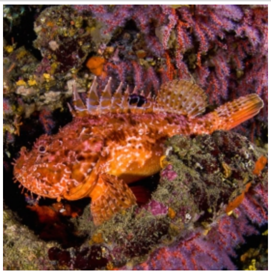

Scorpaena spp.

0 ☐ + ☐ ++ ☐ +++ ☐

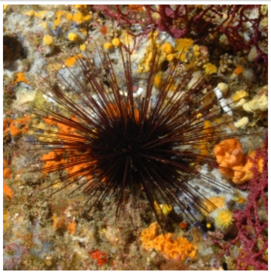

Centrostephanus longispinus

0 ☐ + ☐ ++ ☐ +++ ☐

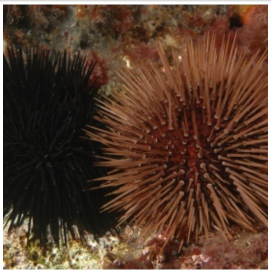

Άλλα είδη αχινών

0 ☐ + ☐ ++ ☐ +++ ☐

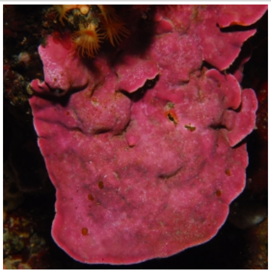

Ενασβεστωμένα ροδοφύκη

0 ☐ + ☐ ++ ☐ +++ ☐

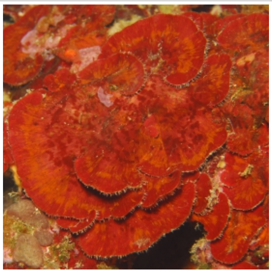

Peyssonnelia spp.

0 ☐ + ☐ ++ ☐ +++ ☐

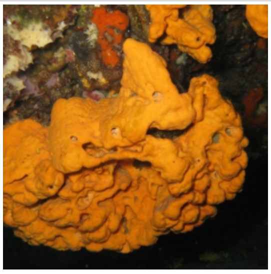

Agelas oroides

0 ☐ + ☐ ++ ☐ +++ ☐

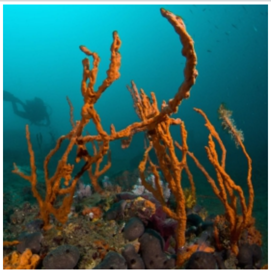

Axinella spp.

0 ☐ + ☐ ++ ☐ +++ ☐

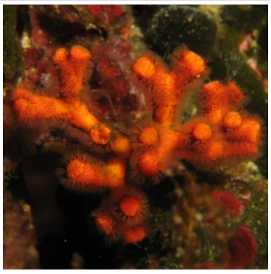

Myriapora truncata

0 ☐ + ☐ ++ ☐ +++ ☐

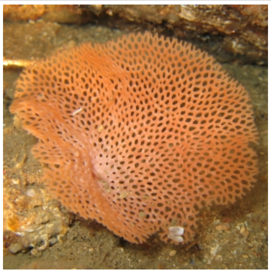

Άλλα βρυόζωα

0 ☐ + ☐ ++ ☐ +++ ☐

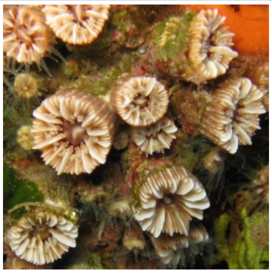

Σκληρακτίνια κοράλλια

0 ☐ + ☐ ++ ☐ +++ ☐

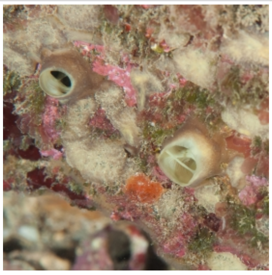

Cliona spp.

0 ☐ + ☐ ++ ☐ +++ ☐

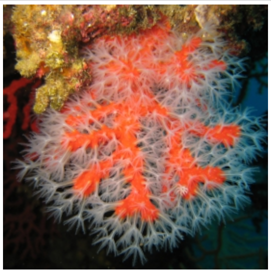

Corallium rubrum

0 ☐ + ☐ ++ ☐ +++ ☐

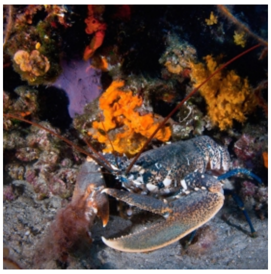

Homarus gammarus

0 ☐ + ☐ ++ ☐ +++ ☐

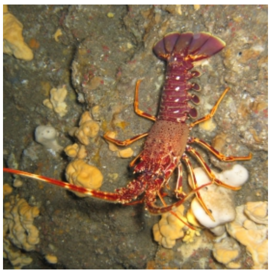

Palinurus elephas

0 ☐ + ☐ ++ ☐ +++ ☐

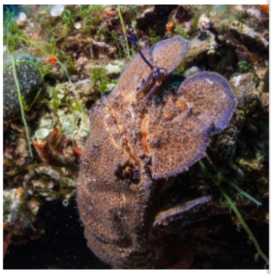

Scyllarides latus

0 ☐ + ☐ ++ ☐ +++ ☐

Θερμοκρασία νερού στο βάθος παρατήρησης:

0 = Απόν + = Σπάνιο
++ = Άφθονο +++ = Πολύ άφθονο

Citizen Science 4 GESMED
