# Supplementary material for: CIGESMED for divers: Establishing a citizen science initiative for the mapping and monitoring of coralligenous assemblages in the Mediterranean Sea
Source: Biodivers Data J. 2016 Nov 1;(4):e8692. doi: 10.3897/BDJ.4.e8692 (PMC5136673; doi:10.3897/BDJ.4.e8692)
Supplement: Supplementary material 9 — CIGESMED per i subacquei - Citizen science per CIGESMED [file biodiversity_data_journal-4-e8692-s009.pdf]

cs.cigesmed.eu/it

Nome

Sito

Data

A che profondità hai incontrato l'acqua più fredda? m / mai

Profondità della osservazione:	Corrente	Visibilità
	Assente ☐ Debole ☐ Forte ☐	Acqua limpida ☐ Qualche particella ☐ Torbida ☐

Estensione dell'habitat

Verticale osservata

Prof. min:

Prof. max:

Orizzontale

<5 m ☐ 5-10 m ☐

10-20 m ☐ >20 m ☐

Continuità dell'habitat

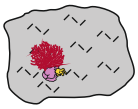 ☐ 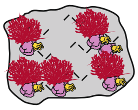 ☐ 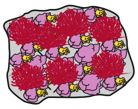 ☐

Inclinazione

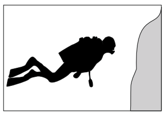 ☐ 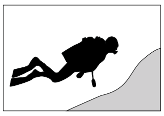 ☐ 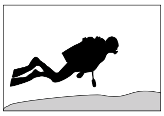 ☐ 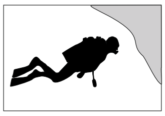 ☐

Rugosità

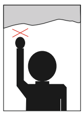 ☐ 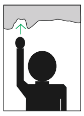 ☐ 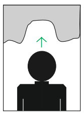 ☐ 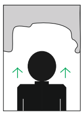 ☐

Esposizione

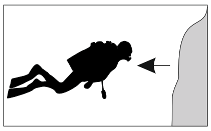

N ☐ S ☐

NE ☐ SO ☐

E ☐ O ☐

SE ☐ NO ☐

Pressioni

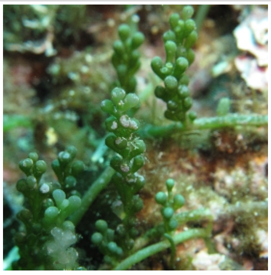

Caulerpa cylindracea

0 ☐ + ☐ ++ ☐

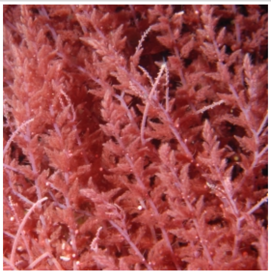

Asparagopsis spp.

0 ☐ + ☐ ++ ☐

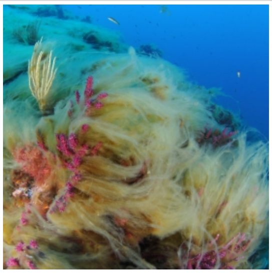

Aggregati mucillaginosi

0 ☐ + ☐ ++ ☐

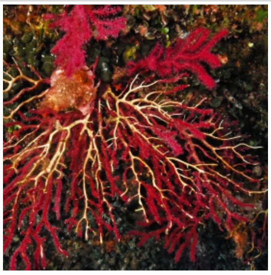

Necrosi/ eventi di mortalità

0 ☐ + ☐ ++ ☐

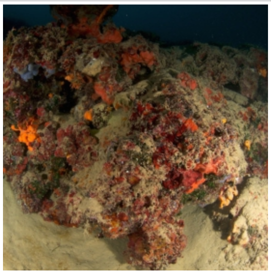

Sedimentazione

0 ☐ + ☐ ++ ☐

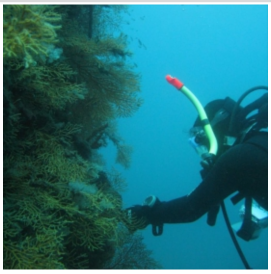

Danni causati dai subacquei

0 ☐ + ☐ ++ ☐

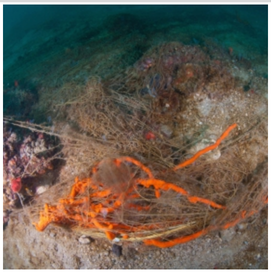

Strumenti da pesca

0 ☐ + ☐ ++ ☐

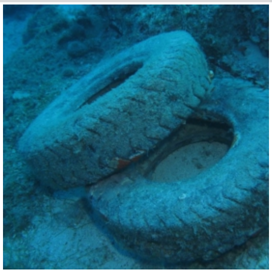

Rifiuti

0 ☐ + ☐ ++ ☐

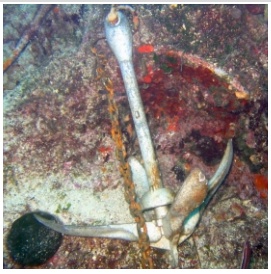

Ancore/ segni d'ancoraggio

0 ☐ + ☐ ++ ☐

0 = assente

\+ = limitato

++ = esteso

Hai osservato qualcos'altro?

Specie

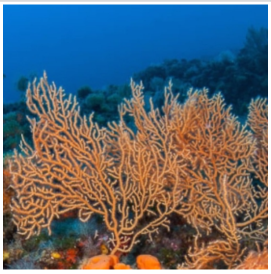

Eunicella cavolini

0 ☐ + ☐ ++ ☐ +++ ☐

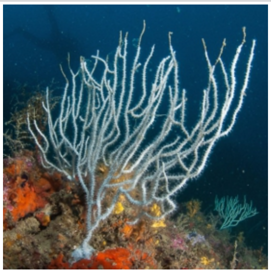

Eunicella singularis

0 ☐ + ☐ ++ ☐ +++ ☐

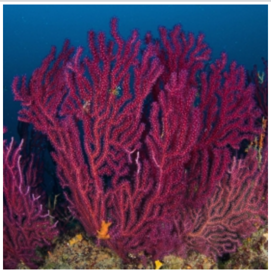

Paramuricea clavata

0 ☐ + ☐ ++ ☐ +++ ☐

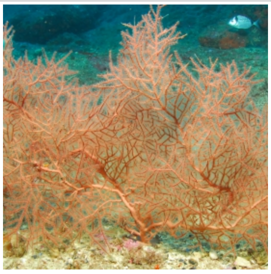

Leptogorgia sarmentosa

0 ☐ + ☐ ++ ☐ +++ ☐

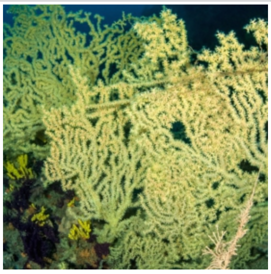

Savalia savaglia

0 ☐ + ☐ ++ ☐ +++ ☐

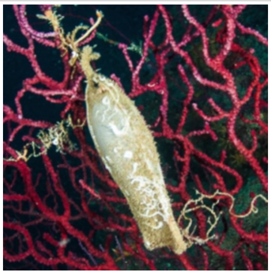

Uova di squalo

0 ☐ + ☐ ++ ☐ +++ ☐

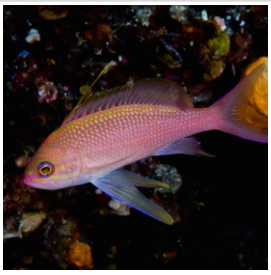

Anthias anthias

0 ☐ + ☐ ++ ☐ +++ ☐

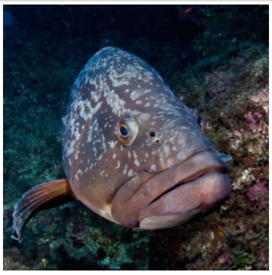

Epinephelus marginatus

0 ☐ + ☐ ++ ☐ +++ ☐

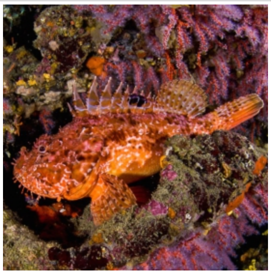

Scorpaena spp.

0 ☐ + ☐ ++ ☐ +++ ☐

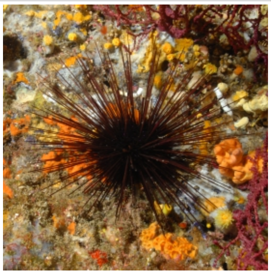

Centrostephanus longispinus

0 ☐ + ☐ ++ ☐ +++ ☐

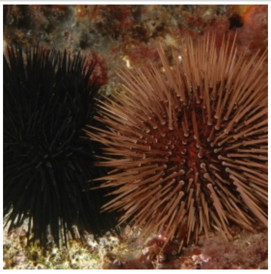

Altri ricci

0 ☐ + ☐ ++ ☐ +++ ☐

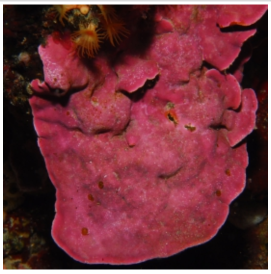

Alghe rosse calcaree

0 ☐ + ☐ ++ ☐ +++ ☐

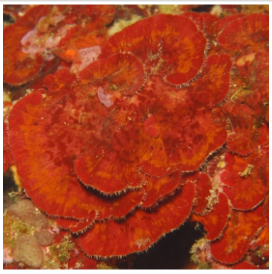

Peyssonnelia spp.

0 ☐ + ☐ ++ ☐ +++ ☐

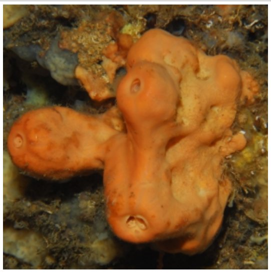

Agelas oroides

0 ☐ + ☐ ++ ☐ +++ ☐

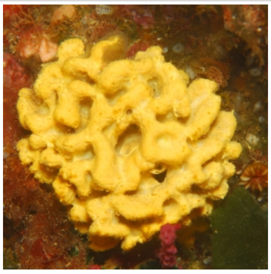

Axinella spp.

0 ☐ + ☐ ++ ☐ +++ ☐

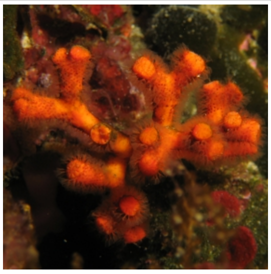

Myriapora truncata

0 ☐ + ☐ ++ ☐ +++ ☐

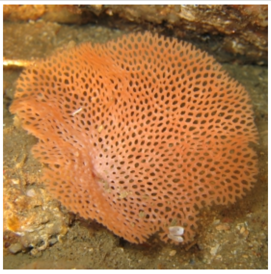

Altri briozoi

0 ☐ + ☐ ++ ☐ +++ ☐

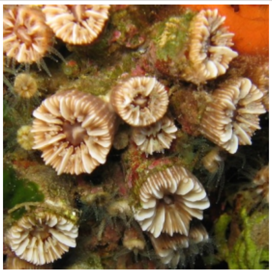

Sclerattinie

0 ☐ + ☐ ++ ☐ +++ ☐

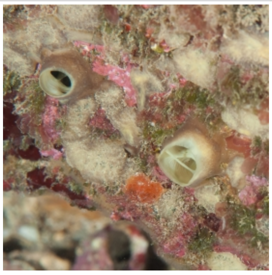

Cliona spp.

0 ☐ + ☐ ++ ☐ +++ ☐

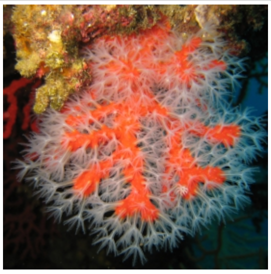

Corallium rubrum

0 ☐ + ☐ ++ ☐ +++ ☐

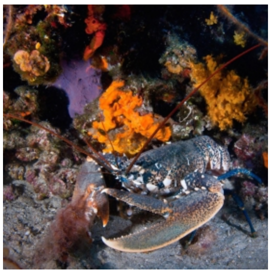

Homarus gammarus

0 ☐ + ☐ ++ ☐ +++ ☐

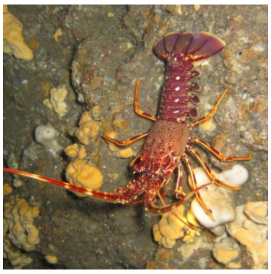

Palinurus elephas

0 ☐ + ☐ ++ ☐ +++ ☐

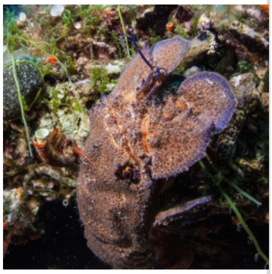

Scyllarides latus

0 ☐ + ☐ ++ ☐ +++ ☐

Temperatura dell'acqua alla profondità dell'osservazione:

Citizen Science 4 GESMED

0 = assente + = scarso ++ = abbondante +++ = molto abbondante
