# Supplementary material for: CIGESMED for divers: Establishing a citizen science initiative for the mapping and monitoring of coralligenous assemblages in the Mediterranean Sea
Source: Biodivers Data J. 2016 Nov 1;(4):e8692. doi: 10.3897/BDJ.4.e8692 (PMC5136673; doi:10.3897/BDJ.4.e8692)
Supplement: Supplementary material 10 — Dalgıçlar için CIGESMED – CIGESMED için Vatandaş Bilimi [file biodiversity_data_journal-4-e8692-s010.pdf]

cs.cigesmed.eu/tr

Gözlemci adı

Bölge

Tarih

Daha soğuk suya hangi derinlikte rastladınız? m / asla

Gözlem derinliği:

Akıntı

Yok ☐ Zayıf ☐ Kuvvetli ☐

Berraklık

Berrak ☐ Az miktarda askı maddesi ☐ Bulanık ☐

Habitatın boyutu

Dikey

Min Derinlik:

Maks Derinlik:

Yatay

<5 m ☐ 5-10 m ☐

10-20 m ☐ >20 m ☐

Habitat sürekliliği

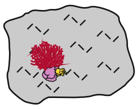 ☐ 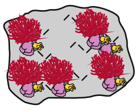 ☐ 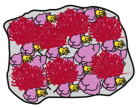 ☐

Eğim

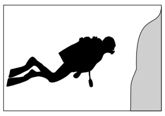 ☐ 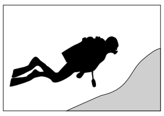 ☐ 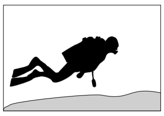 ☐ 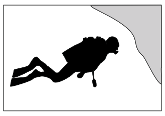 ☐

Pürüzlülük

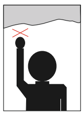 ☐ 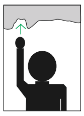 ☐ 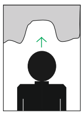 ☐ 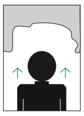 ☐

Yön

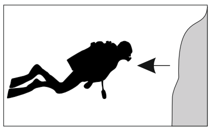

K ☐ G ☐
KD ☐ GB ☐
D ☐ B ☐
GD ☐ KB ☐

Baskılar

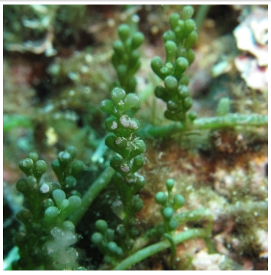

Caulerpa cylindracea

0 ☐ + ☐ ++ ☐

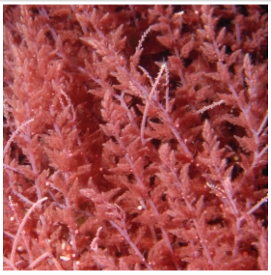

Asparagopsis spp.

0 ☐ + ☐ ++ ☐

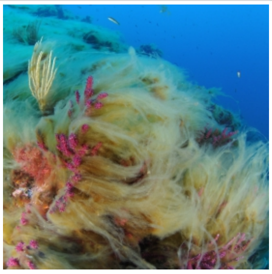

Müsilaj birikimi

0 ☐ + ☐ ++ ☐

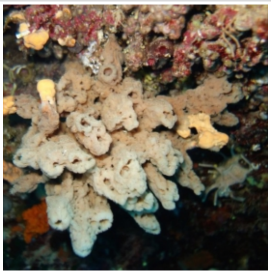

Organizmada doku ölümü / ölüm olayları

0 ☐ + ☐ ++ ☐

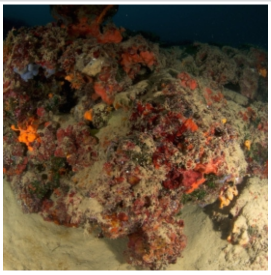

Sediment birikimi

0 ☐ + ☐ ++ ☐

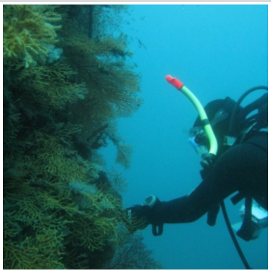

Dalgıç dikkatsizliği

0 ☐ + ☐ ++ ☐

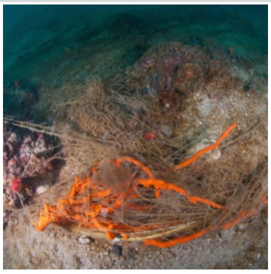

Balık ağı

0 ☐ + ☐ ++ ☐

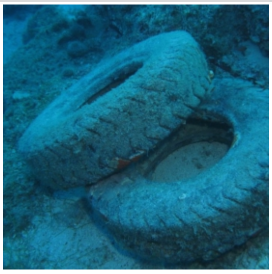

Katı atık

0 ☐ + ☐ ++ ☐

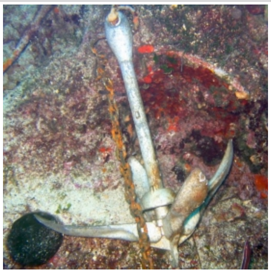

Demirleme

0 ☐ + ☐ ++ ☐

0 = Yok
+ = Sınırlı
++ = Yaygın

Başka birşey gözlemlediniz mi?

Türler

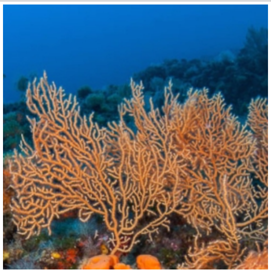

Eunicella cavolini

0 ☐ + ☐ ++ ☐ +++ ☐

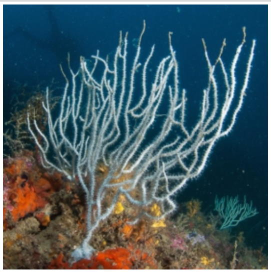

Eunicella singularis

0 ☐ + ☐ ++ ☐ +++ ☐

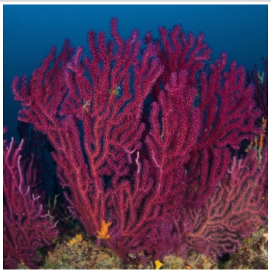

Paramuricea clavata

0 ☐ + ☐ ++ ☐ +++ ☐

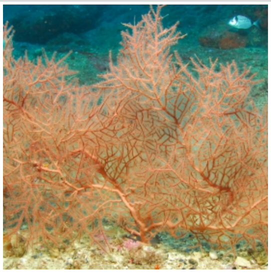

Leptogorgia sarmentosa

0 ☐ + ☐ ++ ☐ +++ ☐

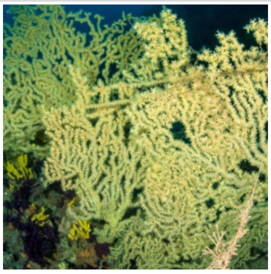

Savalia savaglia

0 ☐ + ☐ ++ ☐ +++ ☐

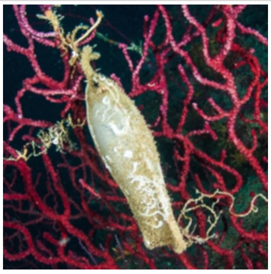

Köpekbalığı yumurtaları

0 ☐ + ☐ ++ ☐ +++ ☐

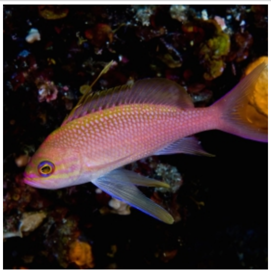

Anthias anthias

0 ☐ + ☐ ++ ☐ +++ ☐

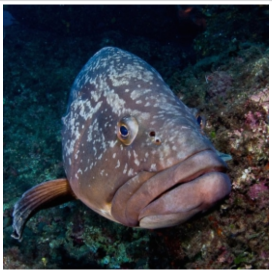

Epinephelus marginatus

0 ☐ + ☐ ++ ☐ +++ ☐

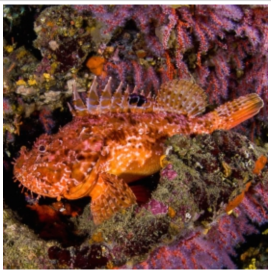

Scorpaena spp.

0 ☐ + ☐ ++ ☐ +++ ☐

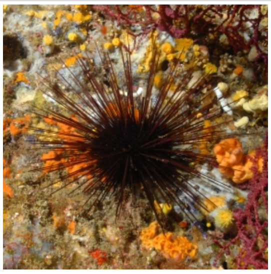

Centrostephanus longispinus

0 ☐ + ☐ ++ ☐ +++ ☐

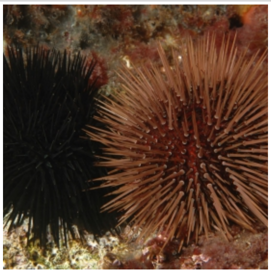

Diğer deniz kestaneleri

0 ☐ + ☐ ++ ☐ +++ ☐

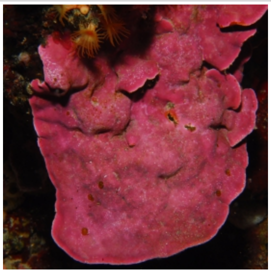

Kalkerli kırmızı algler

0 ☐ + ☐ ++ ☐ +++ ☐

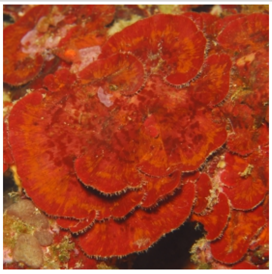

Peyssonnelia spp.

0 ☐ + ☐ ++ ☐ +++ ☐

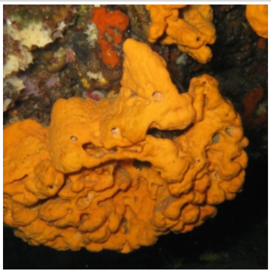

Agelas oroides

0 ☐ + ☐ ++ ☐ +++ ☐

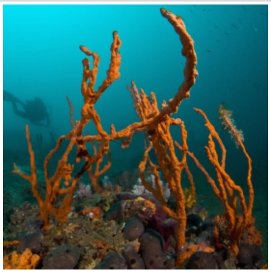

Axinella spp.

0 ☐ + ☐ ++ ☐ +++ ☐

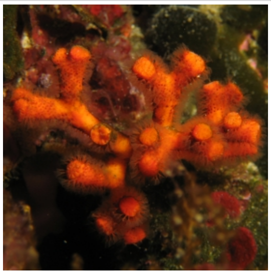

Myriapora truncata

0 ☐ + ☐ ++ ☐ +++ ☐

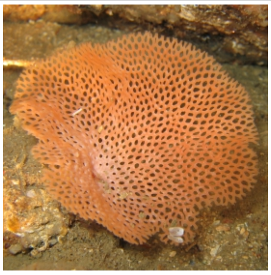

Diğer dikine büyüyen bryozoonlar

0 ☐ + ☐ ++ ☐ +++ ☐

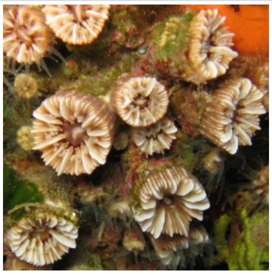

Skleraktinianlar

0 ☐ + ☐ ++ ☐ +++ ☐

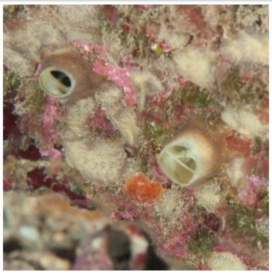

Cliona spp.

0 ☐ + ☐ ++ ☐ +++ ☐

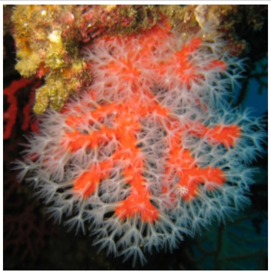

Corallium rubrum

0 ☐ + ☐ ++ ☐ +++ ☐

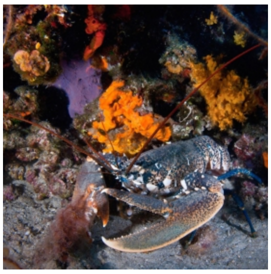

Homarus gammarus

0 ☐ + ☐ ++ ☐ +++ ☐

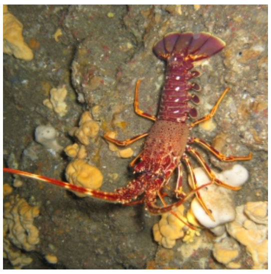

Palinurus elephas

0 ☐ + ☐ ++ ☐ +++ ☐

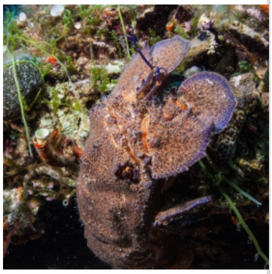

Scyllarides latus

0 ☐ + ☐ ++ ☐ +++ ☐

0 = Yok
+ = Nadir
++ = Bol
+++ = Çok bol

Gözlem derinliğinde su sıcaklığı:

Citizen Science 4 GESMED
